# Tobramycin and bicarbonate synergise to kill planktonic *Pseudomonas aeruginosa*, but antagonise to promote biofilm survival

**DOI:** 10.1038/npjbiofilms.2016.6

**Published:** 2016-05-25

**Authors:** Karishma S Kaushik, Jake Stolhandske, Orrin Shindell, Hugh D Smyth, Vernita D Gordon

**Affiliations:** 1 Department of Molecular Biosciences, University of Texas, Austin, TX, USA; 2 Institute of Cellular and Molecular Biology, University of Texas, Austin, TX, USA; 3 Center for Nonlinear Dynamics and Department of Physics, University of Texas, Austin, TX, USA; 4 Division of Pharmaceutics, College of Pharmacy, University of Texas, Austin, TX, USA

## Abstract

Increasing antibiotic resistance and the declining rate at which new antibiotics come into use create a need to increase the efficacy of existing antibiotics. The aminoglycoside tobramycin is standard-of-care for many types of *Pseudomonas aeruginosa* infections, including those in the lungs of cystic fibrosis (CF) patients. *P. aeruginosa* is a nosocomial and opportunistic pathogen that, in planktonic form, causes acute infections and, in biofilm form, causes chronic infections. Inhaled bicarbonate has recently been proposed as a therapy to improve antimicrobial properties of the CF airway surface liquid and viscosity of CF mucus. Here we measure the effect of combining tobramycin and bicarbonate against *P. aeruginosa*, both lab strains and CF clinical isolates. Bicarbonate synergises with tobramycin to enhance killing of planktonic bacteria. In contrast, bicarbonate antagonises with tobramycin to promote better biofilm growth. This suggests caution when evaluating bicarbonate as a therapy for CF lungs infected with *P. aeruginosa* biofilms. We analyse tobramycin and bicarbonate interactions using an interpolated surface methodology to measure the dose–response function. These surfaces allow more accurate estimation of combinations yielding synergy and antagonism than do standard isobolograms. By incorporating predictions based on Loewe additivity theory, we can consolidate information on a wide range of combinations that produce a complex dose–response surface, into a single number that measures the net effect. This tool thus allows rapid initial estimation of the potential benefit or harm of a therapeutic combination. Software code is freely made available as a resource for the community.

## Introduction

A worldwide increase in antibiotic resistance has prompted the search for newer therapeutic strategies, including adjunct treatment approaches that can extend the lifetime of current antibiotics.^1^ *Pseudomonas aeruginosa* is an opportunistic and nosocomial human pathogen and multi-drug resistant strains are on the rise.^2^ *P. aeruginosa* causes both acute and chronic infections.^3^ Acute *P. aeruginosa* infections are found in burn wounds and surgical sites, and are often initiated by planktonic bacteria. Acute *P. aeruginosa* infections are often resistant to antibiotic treatment and can result in delayed healing, sepsis and death.^4^ For cases where infection transitions from acute to chronic states, planktonic bacteria typically organise to develop biofilm structures.^5^ Chronic *P. aeruginosa* infections are found in chronic wounds in patients with diabetes^6^ and in the lungs of patients with cystic fibrosis (CF)^7,8^ and chronic obstructive pulmonary disease, and are caused by multicellular biofilm aggregates. In CF patients, early *P. aeruginosa* infections are planktonic, intermittent and susceptible to antimicrobial therapy. During long-term infection, it has been suggested both that infecting *P. aeruginosa* converts to the biofilm phenotype and that infecting *P. aeruginosa* persists as a slow-growing, airway-adapted, stationary-phase population; either of these scenarios results in a chronic infection that is notoriously recalcitrant to antibiotic therapy.^9–11^

For both planktonic and biofilm *P. aeruginosa* infections, aminoglycosides are a well-established standard of care.^12^ For CF, the inhaled aminoglycoside tobramycin is widely used as a long-term therapy. Tobramycin has toxic side effects on auditory and kidney function.^13^ Ototoxic side effects include dizziness, tinnitus and irreversible hearing loss.^13^ Nephrotoxic side effects, though reversible, can lead to renal insufficiency, and are exacerbated with prolonged duration of therapy.^13^ Reducing the dose and duration of tobramycin needed for clinical benefit would reduce toxic side effects. It has recently been shown that alkaline pH, mediated by biogenic bases produced by bacteria or exogenous alkalis such as bicarbonate, may enhance the efficacy of aminoglycosides.^14–16^

Independently, the base bicarbonate has an important place in the pathology of CF. In CF patients, a defect in the cystic fibrosis transmembrane conductance regulator (CFTR) impairs bicarbonate transport, leading to acidification of the lung.^17–19^ In a newborn CF pig model,^20^ low pH of the airway surface liquid reduces the activity of innate antimicrobial factors, but antimicrobial efficacy could be restored by aerosolising bicarbonate into the lung. In addition, CF lungs have thick, sticky mucus and impaired mucociliary transport; these promote the growth of bacterial infections.^21^ It was recently proposed that bicarbonate, by chelation of cationic bridges, could help thin mucus, for better clearance.^22^ Therefore, in clinical trials, inhaled bicarbonate is being evaluated as a potential therapeutic approach for CF patients.^22^

Here we evaluate the potential of bicarbonate as an adjunctive therapy to enhance the efficacy of tobramycin against planktonic and biofilm *P. aeruginosa*. We find that the combination of bicarbonate and tobramycin shows a strong synergistic effect against planktonic *P. aeruginosa*, for both laboratory strains and clinical CF isolates. Synergy not only reduces the concentration of tobramycin required to kill *P. aeruginosa* cells but, for some strains, also enhances the rate of killing. However, for biofilms of *P. aeruginosa*, the combination of tobramycin and bicarbonate is markedly antagonistic—i.e., the biofilm survives treatment with the combination better than it survives treatment with the corresponding concentration of tobramycin or bicarbonate alone. Although the synergistic effect against planktonic *P. aeruginosa* cells holds promise, the antagonistic effect against biofilms prompts caution in the development of bicarbonate as a CF therapy.

We quantitatively analyse the synergy and antagonism observed using dose–response surfaces,^23,24^ which examine drug combinations in greater detail than do more traditional methods, and we make our software code for this available for use by the scientific community (Supplementary Material). This dose–response surface analysis allows a more in-depth study of the tobramycin+bicarbonate combinations to more accurately estimate regimes or synergy and antagonism. Furthermore, we can compare measured surfaces with surfaces predicted for additive interactions,^25^ to determine whether the net outcome of a wide range of combinations is additive, synergistic or antagonistic. For systems with complex dose–response curves that show synergy in some parameter spaces and antagonism in others, this kind of combined-surface analysis could be developed into a tool for rapid assessment and making treatment decisions.

## Results

### Combination of tobramycin and bicarbonate produces synergy against planktonic cells of *P. aeruginosa*

**Figure 1:**
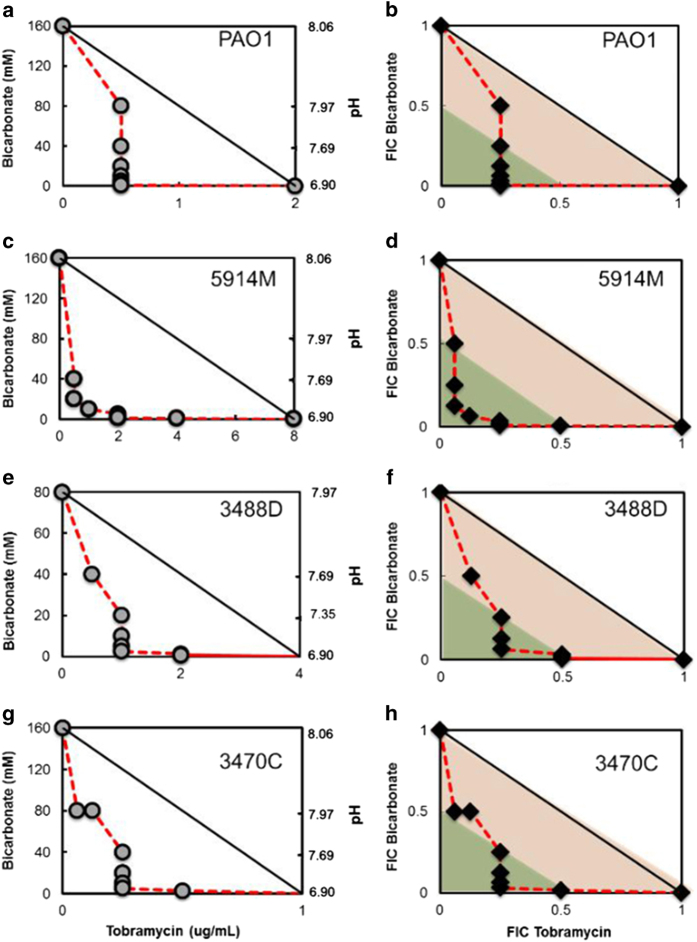
Isobologram analyses for tobramycin–bicarbonate combination against planktonic cells of *P. aeruginosa* (**a**, **b**) laboratory strain PAO1, and *P. aeruginosa* clinical cystic fibrosis isolates (**c**, **d**) 5914M (mucoid), (**e**, **f**) 3488D (dwarf) and (**g**, **h**) 3470C (classic). A strong synergistic-additive effect is observed against all four strains. Points along the isobologram represent the growth–no growth interface. (**b**, **d**, **f**, **h**) The orange shaded area represents the additive region and the green shade area represents the synergistic region.

Using the checkerboard microdilution assays, we test combinations of tobramycin and bicarbonate against planktonic cells of a wide range of *P. aeruginosa* strains: lab strains PAO1 and PA14, four spontaneously generated antibiotic-resistant mutants in the PA14 background, and clinical isolates from CF patients. Cultures were grown overnight so that cells were in the stationary phase, and the next day these stationary-phase cells were added to different combinations of tobramycin and bicarbonate. We create isobolograms by plotting points describing the growth/no-growth interface such that the tobramycin concentration is the abscissa and the bicarbonate concentration is the ordinate. We calculated the ΣFIC (fractional inhibitory concentration) index for each well along the growth/no-growth interface, where the lowest concentration of tobramycin alone or bicarbonate alone that results in no growth is defined as the minimum inhibitory concentration (MIC) for that substance. Addition of bicarbonate to tobramycin produces a synergistic-to-additive effect for all the strains tested (Figure 1 and Supplementary Figures S1–S6). Notably, even though a mild increase in pH from neutral conditions does not inhibit growth in the absence of tobramycin, these relatively small changes in pH in the presence of tobramycin produce a net synergistic effect. Supplementary Table S1 summarises the results for all strains tested. Supplementary Table S2 shows measured pH of LB medium at the relevant bicarbonate concentrations.

**Figure 2:**
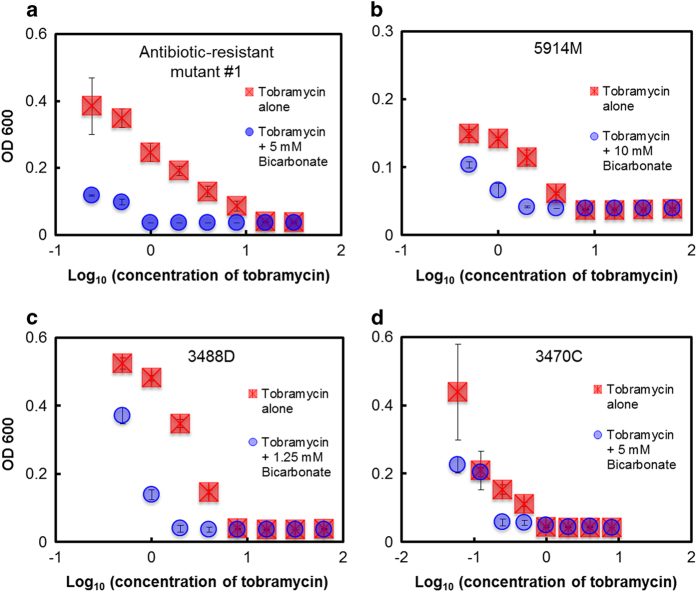
Synergy between fixed combinations of bicarbonate and tobramycin in killing *P. aeruginosa* (**a**) antibiotic-resistant mutant strain #1 and clinical CF isolates (**b**) 5914M, (**c**) 3488D and (**d**) 3470C. Error bars represent s.e.m.; *N*=3. For this, the bicarbonate concentration associated with the lowest ΣFIC value for the select strain was plotted against varying tobramycin concentrations tested. For all four strains thus examined, addition of bicarbonate reduces the concentration of tobramycin required to inhibit planktonic *P. aeruginosa* cells even at tobramycin concentrations much lower than that needed to produce synergy.

Further, for selected antibiotic-resistant and clinical strains, we evaluate combinations using a fixed concentration of bicarbonate. For this, the bicarbonate concentration associated with the lowest ΣFIC value for the select strain was plotted against varying tobramycin concentrations tested. For the strains thus examined, we find that the addition of bicarbonate reduces the concentration of tobramycin required to inhibit planktonic *P. aeruginosa* cells even at tobramycin concentrations much lower than that needed to produce synergy (Figure 2, Supplementary Figures S11–S13).

From these results, we infer that combination with bicarbonate may reduce the concentration of tobramycin needed for clinical benefit against acute infections associated with planktonic *P. aeruginosa* cells and may help treat tobramycin-resistant infections. If so, then this would reduce the dosage and ameliorate toxicity, associated with long-term tobramycin administration. For inhaled tobramycin powder, which is the standard of care for CF lung infections, a standard dose is 112 mg twice daily. Reducing the amount of tobramycin needed for efficacy required would reduce the burden of inhaling so much powder.

### Bicarbonate enhances the rate at which tobramycin kills planktonic *P. aeruginosa* cells

In addition to dosage, a reduction in the duration of tobramycin therapy would also help reduce toxic side effects and antibiotic selection pressure. Furthermore, understanding how the rate of killing is impacted by the addition of bicarbonate to tobramycin will be important for clinical translation. Standard *in vitro* studies are done without drug clearance; so that the concentration of drug is constant over time, and these will under-estimate the actual concentrations required *in vivo*, where drug clearance initiates as soon as the drug is administered. Thus, improving the rate of killing as well as the dose required for killing should give rise to additional benefits *in vivo*.

Therefore, we examine the rate at which the combination of tobramycin and bicarbonate produced inhibition of planktonic *P. aeruginosa* cells. To examine the time-kill kinetics of the tobramycin and bicarbonate combination, we use the lab strain PAO1, antibiotic-resistant strain 1 and the clinical isolate 5913C. Using the concentrations of tobramycin and bicarbonate that show the greatest synergy (i.e., the lowest ΣFIC index) in the checkerboard microdilution assays, we measure the rate of killing of the combination and each agent alone (at combination concentration and MIC) every 2 h for the first 8 h (0, 2, 4, 6, 8 h time points) and then at 24 h.

**Figure 3:**
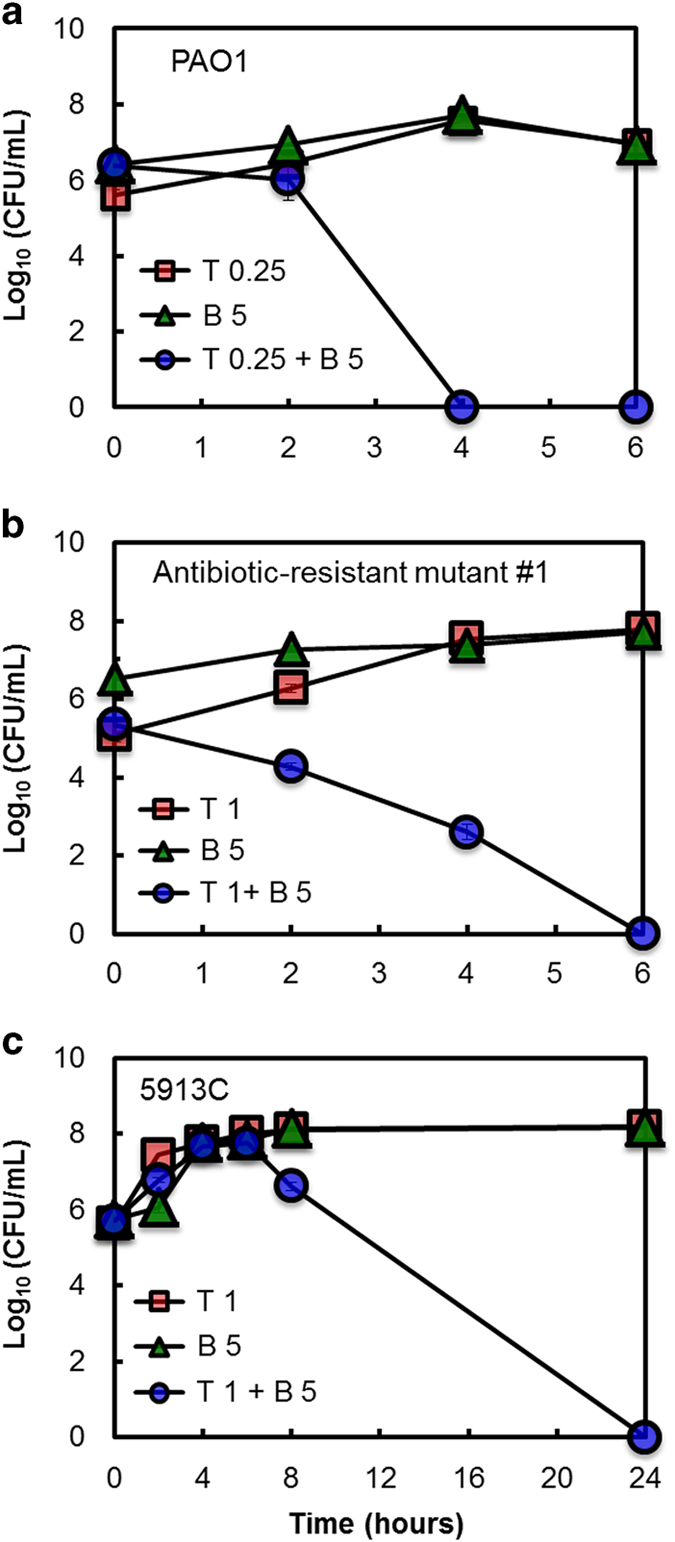
Time-kill assays demonstrating the synergy between bicarbonate and tobramycin against *P. aeruginosa* strains (**a**) PAO1, (**b**) antibiotic-resistant PA14 mutant #1 and (**c**) 5913C. Concentrations of tobramycin and bicarbonate tested represent the combinations that produced the lowest ΣFIC value. For strain PAO1, 0.25 μg/ml tobramycin and 5 mmol bicarbonate; for antibiotic-resistant PA14 mutant #1, 1 μg/ml tobramycin and 5 mmol bicarbonate; and for strain 5913C, 1 μg/ml tobramycin and 5 mmol bicarbonate were tested alone and in combination. Error bars represent s.e.m. (in some cases error bars are smaller than symbols). *N*=3.

For PAO1, treatment with a combination of 0.25 μg/ml tobramycin and 5 mmol bicarbonate eradicates the population by 4 h; in contrast, each agent alone does not kill the population in this time (Figure 3a). Notably, the timescale for killing by the combination is comparable to that for 2 μg/ml tobramycin, the MIC for this strain (Supplementary Figure S14A). This indicates that the combination reduces the concentration of tobramycin needed to eradicate the population by nearly ×10.

For antibiotic-resistant strain #1, treatment with a combination of 1 μg/ml tobramycin and 5 mmol bicarbonate kills the population by 6 h, while each agent alone does not kill the population in this time (Figure 3b). Treatment with 8 μg/ml tobramycin, this strain's MIC, takes >6 h to kill the population (Supplementary Figure S14B). Thus, for this strain bicarbonate not only reduces the necessary concentration of tobramycin by 8×, but also reduces the needed duration of exposure.

For the CF isolate strain 5913C, the synergistic combination of 1 μg/ml tobramycin and 5 mmol bicarbonate begins killing only after 6 h of exposure; the population is entirely killed between 8 and 24 h after exposure to the combination (Figure 3c). At 8 h, the killing curve for the combination lags that of the tobramycin MIC by 7×. (Supplementary Figure S14C). However, both treatments eradicate the population at the end of 24 h, with the combination treatment affording a decrease in the concentration of tobramycin. Thus, for this strain the addition of bicarbonate reduces the concentration of tobramycin needed for killing, but at the cost of increasing the exposure time needed. These results reiterate that the effect of the combination of tobramycin and bicarbonate shows notable strain-to-strain variation.

### For *P. aeruginosa* biofilms, the combination of tobramycin and bicarbonate is antagonistic

**Figure 4:**
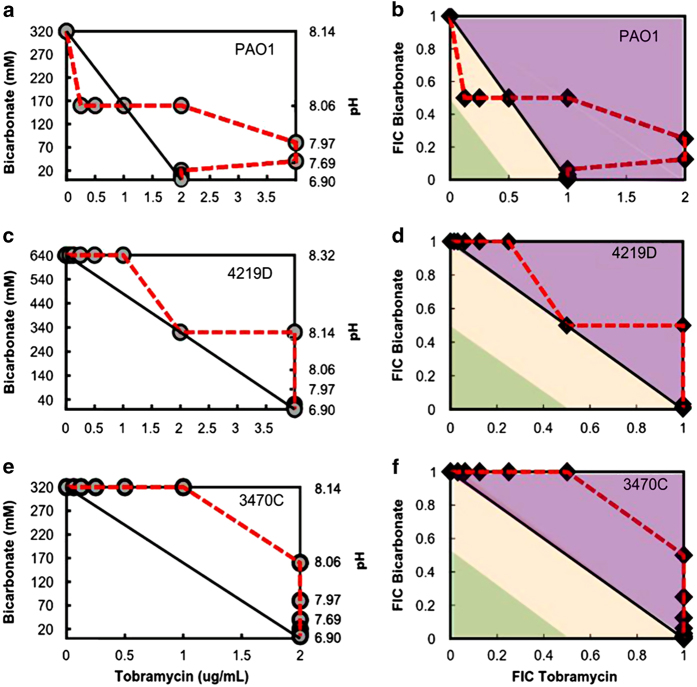
Isobologram analysis for biofilm cells of *P. aeruginosa* (**a**, **b**) PAO1, (**c**, **d**) 4219D and (**e**, **f**) 3470C. An additive-antagonistic effect is observed for the combination of tobramycin and bicarbonate against biofilm cells, in contrast to the strong synergistic effect observed with planktonic cells. Points along the isobologram represent the growth–no growth surface. (**b**, **d**, **f**) The orange shaded area represents the additive region, the purple shaded area represents the antagonistic region, and the green-shaded area represents the region of synergy.

Although we observe notable strain-to-strain variation, combination of tobramycin and bicarbonate show a synergistic effect for planktonic cells of all strains tested. In the planktonic state, *P. aeruginosa* is typically associated with acute infections and sepsis, but chronic *P. aeruginosa* infections are typically biofilm in nature. Therefore, we evaluate the effects of the tobramycin and bicarbonate combination against *in vitro,* laboratory-grown biofilms using the lab strain PAO1 and CF clinical isolates 4219D and 3470C. For all three strains, overnight, 24-h biofilms demonstrate high biomass and metabolic activity (Supplementary Figure S3). Results are summarised in Supplementary Table S3. In striking contrast to the case for their planktonic counterparts, these data show at best an additive effect, and at worst a strongly antagonistic effect. This is shown by FIC values >1 (Supplementary Table S3) and by isobolograms that extend to the right of the line of additivity (Figure 4). We speculate that the difference in the response of biofilms to combination treatment with tobramycin and bicarbonate, compared with the response of planktonic cells to combination treatment, may arise from acidification of the biofilm environment or limited diffusion into the interior of the biofilm.^26,27^ Alternatively, we may be detecting more metabolically active bacteria in the biofilm if the pH change induces otherwise-inactive bacteria into an active state.

### Tobramycin+bicarbonate combinations have an additive effect against stationary-phase cells

To determine the degree to which the antagonism observed for the combination of tobramycin+bicarbonate against *P. aeruginosa* biofilms is a biofilm-specific phenomenon, distinct from an effect arising from the presence of high-density, stationary-phase cells, we performed the checkerboard assay with different combinations of tobramycin+bicarbonate using a high density of stationary-phase PAO1 cells, as described in more detail in the Materials and Methods section.

At this high density of cells, the efficacy of both tobramycin and bicarbonate was reduced. This is likely the result of the inoculum effect, or the per-cell concentration of antibiotic or antibacterial agent being more important for whether or not a cell is killed than is the absolute (Molar) concentration. As a result, we were unable to use a 90% threshold to describe MIC. Therefore, we used ~50% inhibition of bacterial growth as the MIC for all subsequent analysis of planktonic, stationary-phase bacteria. Using isobologram analysis and FIC values (Supplementary Figure S15), we found an additive effect for tobramycin+bicarbonate combinations against stationary-phase PAO1 cells.

### The antagonistic effect on *P. aeruginosa* biofilms is likely due to a combination of biofilm-specific properties and presence of stationary-phase cells

To examine the degree to which planktonic bacteria might be more susceptible to killing because of their exponential growth phase, as opposed to the quasi-stationary-phase state of many biofilm bacteria, we examined the lag phase of PAO1 bacteria introduced into different concentrations of tobramycin alone, bicarbonate alone and combinations of tobramycin and bicarbonate (Supplementary Figures S16–S18). In no case was the lag time shorter than 200 min (this was found for combinations that we had previously found to be well below the inhibitory threshold). Moreover, for combinations just below the growth–no growth interface, bacteria did not re-enter exponential growth over the time of observation (360 min). This suggests that some killing may happen before early exponential phase begins—i.e., some killing happens at some point in the lag phase characterising the dilution from stationary phase into fresh medium.

We recall our finding that stationary-phase bacteria are less susceptible to tobramycin-bicarbonate combinations than are diluted (and subsequently exponentially growing) bacteria, but that stationary-phase bacteria are also more susceptible to tobramycin-bicarbonate combinations than are biofilm bacteria. Taken together, these findings suggest that the antagonism found for bicarbonate+tobramycin combinations against biofilms (Figure 4) is likely multifactorial in origin. In addition to stationary-phase cells, biofilms also have phenotypically specific characteristics such as the extra-bacterial polymer+protein matrix and acidified interiors. These may act to turn additivity, against stationary-phase bacteria, into antagonism, against biofilms.

### Response-surface analysis

The contrast between exponentially growing planktonic, stationary-phase planktonic and biofilm results highlights the complexity of this system and points up the need for methods of analysis that can quantitatively describe variable behaviour over a broad parameter-space. Isobolograms are a standard and widely used approach to examine drug interactions.^28^ Although they offer valuable insights, they do have limitations:

1. The actual MIC value of an agent or combination of agents may be overestimated when testing using serial dilutions. This results in an underestimation of synergy.
2. Plotting isobolograms from the growth–no growth interface implicitly neglects the potential therapeutic benefits of a partial inhibitory effect (less than MIC).

**Figure 5:**
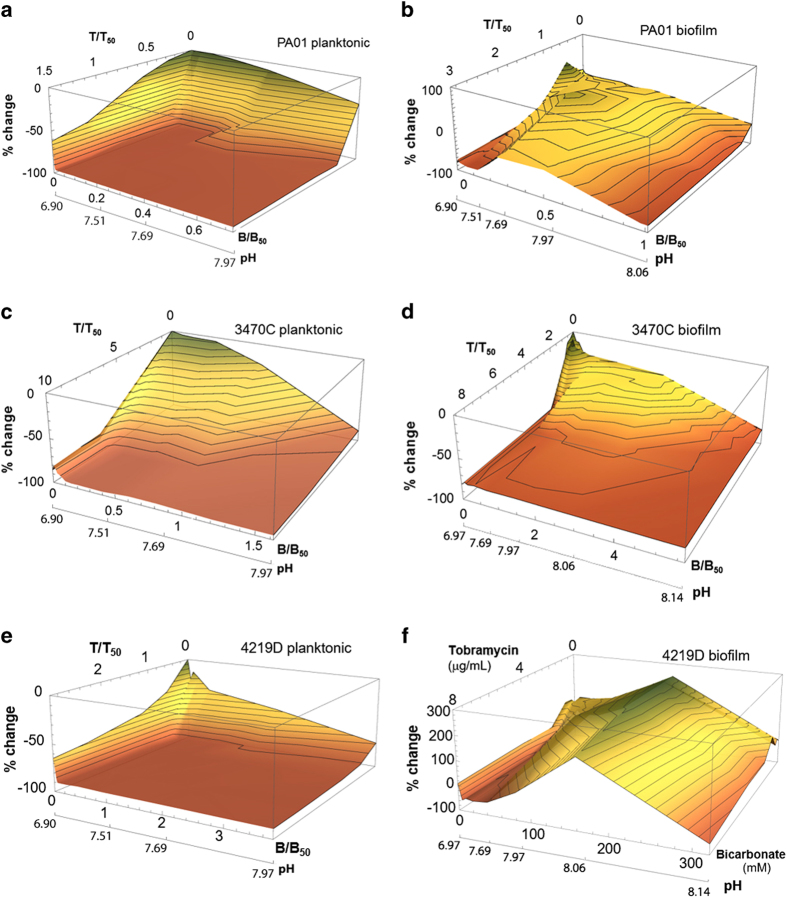
Measured response surfaces for tobramycin and bicarbonate treatments of (**a**, **c**, **e**) planktonic bacteria and (**b**, **d**, **f**) biofilms. Contour lines show increments of 10% change. (**a**–**e**) Tobramycin and bicarbonate concentrations are plotted as the fraction of the concentration that produces 50% growth inhibition. (**f**) For the 4219D biofilm, the one-dimensional curves along the bicarbonate axis (with no tobramycin) and along the tobramycin axis (with no bicarbonate) are non-monotonic. This prevents determination of 50% growth inhibition values and therefore actual concentrations are used instead. (**a**, **e**) For PAO1 and 4219D, the planktonic response surface is steeply concave upward, reflecting synergy between tobramycin and bicarbonate for planktonic bacteria. (**c**) For 3470C, the response surface is only shallowly curved, indicating little overall synergy for planktonic bacteria. (**b**, **d**, **f**) For all strains, the biofilm response surface is concave downward. This reflects synergy between tobramycin and bicarbonate for planktonic bacteria and antagonism between tobramycin and bicarbonate for biofilm bacteria. (**b**, **f**) For PAO1 and 4219D, portions of the biofilm response surface extend above 0% change, indicating that combination of tobramycin and bicarbonate can enhance viability over that of the untreated biofilm.

To examine these drug combinations in greater detail than traditional methods, we develop a method of response-surface analysis. Using second-order polynomial interpolation of the checkerboard assays, we construct analytical dose–response surfaces that approximate the complex nature of nonlinear drug interactions with far greater resolution and nuance than allowed by traditional isobolograms. Measured response surfaces for planktonic and biofilm PAO1, 3470C, 4219D and 3470C are shown in Figure 5. The concave-upward response surfaces characterising the planktonic bacteria indicate synergy between bicarbonate and tobramycin; the concave-downward response surfaces characterising the biofilms indicate antagonism.

Furthermore, we use the measured effects of both tobramycin alone and bicarbonate alone to calculate surfaces describing Loewe additivity, as detailed in Materials and Methods. These surfaces are shown in Supplementary Figures S19–S21. The contrast with the measured response surfaces (Figure 5) is striking and consistent with our finding that neither planktonic nor biofilm bacteria respond additively to a treatment by a combination of tobramycin and bicarbonate.

**Figure 6:**
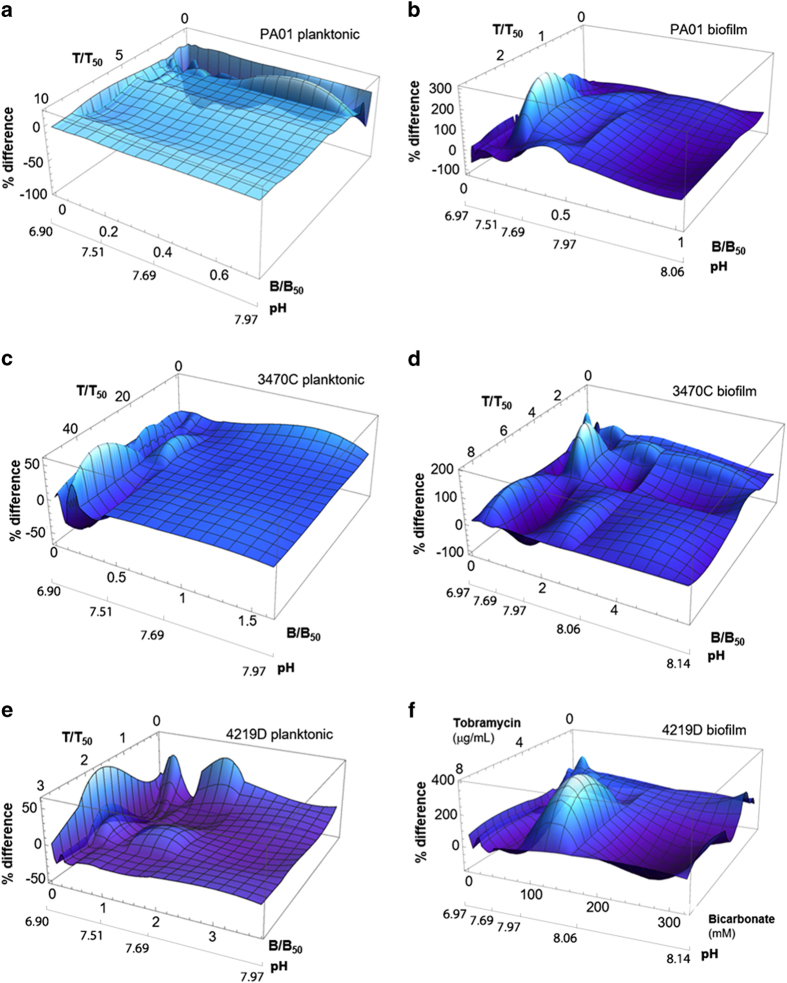
Difference surfaces measure the departure of measured response from that expected from Loewe additivity for (**a**, **c**, **e**) planktonic bacteria and (**b**, **d**) biofilms. (**f**) For 4219D biofilms, we measure the departure from a strictly additive surface. By our construction, a negative value for % difference indicates a synergistic interaction between tobramycin and bicarbonate at the corresponding combination, and a positive height indicates an antagonistic interaction. (**a**) The difference surface for planktonic PAO1 is consistently negative within the 0% difference border, indicating that the interaction between tobramycin and bicarbonate is everywhere synergistic. This surface is rendered semi-transparent for better visualisation. (**b**) The difference surface for biofilm PAO1 is positive at most locations, indicating overall antagonism, and negative at a few locations, indicating that within that limited parameter space the tobramycin-bicarbonate interaction is synergistic against the biofilm. (**c**) The difference surface for planktonic 3470C bacteria ranges between −50 and +50%, and the integral of this surface over the region shown is close to zero. This indicates that the net synergy between tobramycin and bicarbonate is negligible when averaged over the combinations measured. (**d**) The difference surface for biofilm 3470C is positive at most locations, indicating overall antagonism, and negative at a few locations, indicating that within that limited parameter space the tobramycin-bicarbonate interaction is synergistic against the biofilm. (**e**) The difference surface for planktonic 4219D bacteria ranges between −50 and +50%, and the integral of this surface over the region shown is −3.9, indicating overall net synergy between tobramycin and bicarbonate. (**f**) For the biofilm, the difference surface for biofilm 4219D is positive at most locations and the integral over the area shown is +67, indicating that the combination of tobramycin and bicarbonate against this biofilm has the strongest overall antagonism of all the systems we measure.

To quantify the departure from additivity, and thereby quantify the degree of synergy or antagonism with the combination, we calculate difference surfaces, which measure the change in response from that expected from Loewe additivity, taking into account measurement error. By our construction, synergy will be indicated by a negative height and antagonism will be indicated by a positive height. Calculated difference surfaces (Figure 6) show, as expected, mostly negative heights for planktonic bacteria and mostly positive heights for biofilm bacteria. Response surface analysis of stationary-phase planktonic bacteria (Supplementary Figure S22) indicates a combination of synergy, additivity and antagonism.

These surfaces are notably rugged, occasionally extending above the zero plane for planktonic bacteria and below the zero plane for biofilms. This complex response is potentially clinically relevant, since drug levels are not expected to be homogenous throughout the airways and infection site in CF. This is highlighted by studies that have conducted quantitative aerosol deposition studies in patients with lung disease and shown non-uniform deposition.^29^ Thus, it is expected that the concentration of each administered drug will vary both with location in the body and with time. Accordingly, an assessment of the net effect of a combination over a given range of concentrations can simplify the complexity of the response surface and indicate whether a net benefit or a net harm is likely to occur. We provide such an assessment by integrating over the measured range of tobramycin and bicarbonate values—for PAO1 planktonic bacteria, this integral has a value of −4.5±0.6, indicating a net synergy, and for PAO1 biofilm bacteria, this integral has a value of +8.50±5.61, indicating a net antagonism. For stationary-phase PAO1 planktonic bacteria, this integral has a value of −0.4±5.3, so the net effect is nearly purely additive and is well resolved from the biofilm-state value of +8.50±5.61 (Supplementary Figure S22). This is in agreement with our isobologram analysis (Supplementary Figure S15) and with our inference that antagonism against biofilms likely results both from the presence of a large number of stationary-phase cells and characteristics specific to the biofilm phenotype. Notably, for the clinical isolate 3470C, the corresponding values are −0.4±0.2 for planktonic bacteria and 10.28±12.92 for biofilm bacteria. This indicates that overall antagonism is likely stronger for 3470C biofilms than for PAO1 and that the overall synergy against 3470C planktonic bacteria is negligible. Finally, the difference surface for planktonic 4219D bacteria ranges between −50 and +50%, and the integral of this surface over the region shown is −10.5 ±1, indicating synergy between tobramycin and bicarbonate that is greater than that for PAO1 planktonic bacteria. However, for the 4219D biofilm, the difference surface is positive at most locations and the integral of the surface shown is +67±~13 (this uncertainty is from the *ad hoc* interpolation method used to create the response surface for the 4219D biofilm, because this biofilm was very poorly described by the Hill function used for all other systems). This indicates that the combination of tobramycin and bicarbonate against this biofilm has the strongest overall antagonism of all the systems we measure.

It is worth noting that this estimation of antagonism deliberately neglects the increased biofilm viability when bicarbonate is used without tobramycin and bicarbonate concentration is in the 100–300 mmol range. Therefore, this estimation of high antagonism substantially underestimates the net benefit to this biofilm of bicarbonate treatment.

## Discussion

Our findings indicate the need for caution when combining tobramycin and bicarbonate in CF treatment.

If the expected distributions of each active agent in the body are known, the integral-based metric described above could be refined by limiting the range of concentrations over which it is performed and by rescaling the concentration axes to correspond to nonlinear probability distributions. Thus, development of new therapeutic approaches such as these studied here need to determine not just the nominal and administered doses, but also anticipate the effects of the local microenvironment and distributions within the infection site. Coupled with the data analysis described above, appropriate doses of the combination therapies can be determined.

## Conclusions

Our results demonstrate that the combination of tobramycin and bicarbonate shows a strong synergistic effect against planktonic *P. aeruginosa* cells. As expected with a synergistic combination, addition of bicarbonate reduced the concentration of tobramycin needed to eradicate planktonic cells; it also significantly enhanced the rate of killing of cells in comparison with inhibitory concentrations of tobramycin alone. This opens the possibility of an additional role for bicarbonate therapy in CF patients, where it could augment the activity of tobramycin therapy against planktonic cells and early *P. aeruginosa* infections. However, the combination of tobramycin and bicarbonate was at best additive against biofilm cells of *P. aeruginosa*, and was in fact antagonistic in most combinations. Since inhaled tobramycin is standard-of-care for CF patients infected with *P. aeruginosa*, and since chronic CF infections are widely thought to be caused by biofilms, our work indicates the need for caution in development of bicarbonate into a CF therapy.

This approach could also hold potential in the management of non-CF infections such as sepsis, urinary tract infections and meningitis. These approaches could also hold promise in the management of acute burn and post-surgical wounds, but it is clear that more work needs to be done to fully understand the effects of local pH at wound infection sites.^30^

Finally, we present an improved method for analysing drug interactions using analytic dose–response surfaces. These allow more accurate and more nuanced estimation of regimes of synergy and antagonism than do standard isobolograms, which have limited analytical and predictive power. Continuous response surfaces have improved potential to find untested synergistic combinations. By combining measured response surfaces with predictions based on Loewe additivity theory, we can consolidate information on the effect of a wide range of combined concentrations into a single number that measures the net effect, both synergistic and additive. This can simplify complex response surfaces to allow rapid initial estimation of the potential benefit or harm of a therapeutic combination. Further information and annotated code is made available in Supplementary Information as a resource for the community.

## Materials and methods

### Bacterial strains and growth conditions

*P. aeruginosa* strains used include wild-type^31^ laboratory strains PA14 and PAO1 and clinical isolates from patients with CF^32^ (gift from Marvin Whiteley, UT Austin). To generate spontaneous antibiotic-resistant mutants, WT PA14 cultures, were grown overnight in antibiotic-free media, and plated on tobramycin 8 μg/ml agar. Antibiotic-resistant mutants grew colonies and were archived in 20–30% glycerol at −80 °C. Of the four independent antibiotic-resistant mutants (strains 1–4) used in this study, strain 1 was used in our previous work, which examined the effect of population spatial structure and metabolism on antibiotic resistance.^15^ As part of this previous work,^15^ this strain was sequenced (Illumina MiSeq) and sequence information is deposited in the NCBI Short-Read Archive (accession no. SRP042054). Three single-nucleotide polymorphisms (SNPs) unique to this strain were identified, in genes TrkH (potassium uptake membrane protein), EF-Tu (elongation factor) and PhzD (phenazine biosynthesis protein); each of these mutations is a plausible candidate, alone or in combination, to confer aminoglycoside resistance.^15^ Elongation factor Tu (EF-Tu) has a crucial role in the elongation phase of bacterial protein synthesis, in which it delivers aminoacylated tRNAs (aa-tRNAs) to the ribosome.^33^ Aminoglycoside antibiotics inhibit bacterial protein synthesis by targeting the ribosome.^34^ Mutations in the eukaryotic elongation factor 1 (EF-1), analogous to bacterial EF-Tu, and other bacterial elongation factors, such as EF-G, have been associated with aminoglycoside resistance,^35^ indicating the possibility that the EF-Tu mutation. TrkH is a hydrophobic, membrane protein and a constituent of the potassium uptake system, which mediates the symport of potassium and hydrogen ions. Mutations in Trk system have been associated with altered aminoglycoside susceptibility. The amino-acid residue affected by one of these mutations was close to the ion channel, possible leading to increased proton influx and diminished membrane potential.^35^ Aminoglycosides require the membrane potential component of the proton motive force for active cellular uptake^36^ and adaptation to this class of antibiotics frequently proceeds through mutations that diminish the generation of proton motive force. The *phzD2* gene is part of the phenazine biosynthetic cluster, encoded by two redundant operons *phzA1-G1* and *phzA2-G2.*^37^ To the best of our knowledge, there is no direct evidence of *phzD* mutations conferring aminoglycoside resistance, and the redundant nature of this gene makes its role further unlikely.

The clinical CF isolates used in this work, have been used in previous work,^32^ and have been subject to transcriptome analysis. This determined that these strains had been acquired a range of adaptive traits during their evolution in the *in vivo* host. The MIC for tobramycin for the clinical CF isolates was tested and reported in Supplementary Table S1 of our previous work.^15^ These MIC values are (in μg/ml) 0.07 for strain 3640D, 0.3 for strains 4219D, 0476M, 0.6 for strains 3470C, 3639M, 4278M, 4218C, 0324C, 2159M, 4220M, 5912M, 1.2 for strains 2773C, 1913C, 3488D, 2.4 for strains 5623M, 5914M and 4.8 for 5913C. In this nomenclature, the terminal ‘C’ indicates a classic colony phenotype, the terminal ‘M’ indicates a mucoid (alginate overproducing) colony phenotype, and the terminal ‘D’ indicates a dwarf, or small-colony, phenotype. Small-colony phenotypes are frequently associated with increased production of the non-alginate extracellular polysaccharides Psl and/or Pel. Increases in the production of alginate, Psl and Pel have been linked to increased tobramycin resistance for biofilms.^38–40^

All bacterial strains were grown in Luria–Bertani broth or on LB agar^41^ except where otherwise indicated. Overnight cultures were shaken at 180 r.p.m. for 16–18 h at 37 °C.

### Antibiotics

Tobramycin (Indofine Chemical Company, Hillsborough, NJ, USA) and sodium bicarbonate (Thermo Fisher Scientific, Waltham, MA, USA) were obtained as standard powders. A stock solution of 50 mg/ml tobramycin was stored at 4 °C prior to use. The desired concentration of sodium bicarbonate solution was freshly prepared prior to use for each experiment.

### Minimum inhibitory concentration determination for planktonic cells

MICs to tobramycin and bicarbonate were measured using broth microdilution methods. MICs were determined by observing visual turbidity and measuring optical density (OD) at 600 nm. The lowest concentration that inhibited visual growth, which corresponded to ~90% inhibition of bacterial growth (reported as MIC), was used for further analysis, except where stated otherwise for undiluted, dense planktonic suspensions.

### Checkerboard assay to study drug interactions against planktonic *P. aeruginosa* cells

Standard checkerboard microdilution assays^42,43^ were used to test the combined antimicrobial activity of tobramycin and bicarbonate against planktonic cells of different *P. aeruginosa* strains. In brief, an 8×8 array of serial twofold dilutions of the two agents were mixed together in a flat-bottom, 96-well microtiter plate (polystyrene) such that each row (or column) contained a fixed amount of one agent and increasing amounts of the second agent. This resulted in a total of 64 different combinations. For each assay, the serial dilutions of each individual agent were tested alone (to measure the MIC), and control wells containing untreated cells were also grown. Bacteria were grown overnight in LB medium (18–24 h, resulting in stationary phase cultures), and the next day ~10^5^ cells from these cultures were added to each well. Each strain was tested in duplicate. Plates were sealed with parafilm and incubated at 37 °C under static conditions. After overnight incubation (16–18 h), optical density was measured at 600 nm (OD_600_). An example of such measurements is given in Supplementary Figure S1A. The FIC was calculated for each well along the growth–no growth interface (corresponding to ~90% inhibition in the presence of the combination, with each agent below its own MIC). For agents A and B, the FIC of the combination is calculated as^23^

$$\Sigma \;{\mathrm{FIC}}_{A+B}={\mathrm{FIC}}_{A}+{\mathrm{FIC}}_{B},\;\mathrm{where}\;{\mathrm{FIC}}_{A}=\frac{{\mathrm{MIC}}_{A+B}}{{\mathrm{MIC}}_{A}}\;\mathrm{and}\;{\mathrm{FIC}}_{B}=\frac{{\mathrm{MIC}}_{A+B}}{{\mathrm{MIC}}_{B}}$$

An example of calculated ΣFIC indices is given Supplementary Figure S1B. The ΣFIC index was interpreted as follows: ΣFIC⩽0.5 indicates a synergistic interaction; ⩾0.5 and <1 indicates an additive effect; and >1 indicates antagonism.

The results of the checkerboard assays were represented graphically using isobolograms. Combinations that fall along the line connecting the MIC values of the two agents (line of additivity) are additive interactions. If the combination is synergistic, the isobol will be overall concave-up. For combinations that are antagonistic, the isobol will be overall concave-down. Supplementary Figure S2 labels additive, synergistic and antagonistic regions on a sample coordinate system. Further, the magnitude of the overall average curvature indicates the degree of synergy or antagonism, respectively.

### Time-kill assays

To examine the rate of killing of the synergistic combination, time-kill assays were performed. Briefly, 96-well microtiter plates were set up using synergistic combinations (from the checkerboard assays), with concentrations of the individual agents alone, and untreated cells as controls. Bacteria (~10^5^ cells) were added to each well. Plates were incubated at 37 °C for 24 h under static conditions. Colony-forming units per ml (CFU/ml) were counted at 0, 2, 4, 6, 8 and 24 h after beginning treatment. To count the number of colonies, serial dilutions of the bacterial suspension were plated on LB agar. Plates were incubated at 37 °C overnight (16–18 h) and CFUs were counted. For each strain tested, assays were performed in duplicate. Each combination or concentration at a given time point was tested in duplicate.

### Measurement of pH

Different concentrations of bicarbonate were added to sterile LB media and the pH was measured using a pH meter (Thermo Scientific Orion 2 Star). Prior to use, the instrument was calibrated using standard solutions of pH 4, 7 and 10.

### Testing the effect of the tobramycin+bicarbonate combination against preformed biofilms

Biofilms were grown in round-bottom, untreated, 96-well microtiter plates as previously described.^44,45^ Briefly, bacterial (~10^5^ cells) were added to well of the microtiter plate in an 8×8 array to test different combinations of tobramycin and bicarbonate. In addition, wells to test serial dilutions of each agent alone, and control wells for untreated cells were also set up. Each strain was tested in duplicate. Plates were sealed with parafilm and incubated at 37 °C under static conditions for 18–24 h (overnight). The next day, wells were washed twice with sterile LB medium. Different combinations of tobramycin and bicarbonate (dissolved in LB medium) were added to the 8×8 array, such that each row (or column) contained a fixed amount of one agent and increasing amounts of the second agent (total volume 150 μl). To obtain the minimum biofilm inhibitory concentration, serial dilutions of each agent were also tested alone (total volume 150 μl). The control wells were replaced with sterile LB medium without any antimicrobial agent. The plates were sealed with parafilm and incubated at 37 °C static for 18–24 h (overnight). The next day, the wells were washed with sterile LB medium twice and effect of the treatment on biofilm cells was assessed with crystal violet staining and the XTT assay.^45,46^

### Crystal violet assay for biofilm mass

The crystal violet assay was performed as previously described.^45^ Briefly, preformed biofilms (both treated and untreated controls) were washed twice with sterile LB medium. Cells were fixed at the bottom and sides of the wells by treating them with 100% methanol for 15 min. Following removal of methanol, 0.1% filter-sterilised crystal violet solution^47^ was added to the well and allowed to stain the biofilm for 30 min. Wells were washed in water and the dye was solubilised using 100% ethanol. Two-hundred microlitres of well contents were transferred to a new, clear, flat-bottom, 96-well plate and absorbance was read at 600 nm.

### XTT assay for metabolic activity in biofilms

The XTT assay was performed as previously described.^46^ Briefly, preformed biofilms (either treated or untreated) were washed twice with sterile LB medium. XTT was dissolved to make a stock solution of 1 mg/ml. For Menadione, a 7 mg/ml stock solution was prepared in acetone and then diluted 1:100 in distilled water. A solution of LB: XTT: Menadione (79:20:1) was prepared. The solution of LB: XTT: Menadione (200 μl) was added to each well, and the plates were incubated at 37 °C in the dark (sealed with aluminum foil) for 4 h. One-hundred-fifty microlitres of well contents were transferred to a new, clear, flat-bottom, 96-well plate and absorbance was read at 492 nm.

### Screen for biofilm formation by *P. aeruginosa* strains

The laboratory strain PAO1 and 17 clinical CF isolates (mucoid, dwarf and classic phenotype),^32^ were screened for biofilm formation using the microtiter dish biofilm assay and quantified using the crystal violet and XTT methods. Using the crystal violet assay, clinical strains 3639M, 4278M, 3470C, 1913C, 0476M, 4220M, 4219D and laboratory strain PAO1 show robust biofilm formation (Supplementary Figure S3A). Crystal violet is a basic dye that binds to negatively charged surface molecules. It thus stains the extracellular matrix, live and dead cells of the biofilm.

To quantify changes in metabolic activity, we use the XTT assay. In metabolically active cells, XTT is reduced to a water-soluble formazan derivative that can be quantified colorimetrically. Strains 3639M, 4278M, 3470C, 1913C, 0476M, 4220M, 4219D and PAO1 that showed robust biofilm formation in the crystal violet assay also demonstrated the presence of live, metabolically active cells in the XTT assay (Supplementary Figure S3B). On the basis of the results of this screen, three strains, PAO1, 4219D and 3470C, were chosen for testing the effects of the tobramycin–bicarbonate combination on biofilms.

### Measurement of the effect of tobramycin+bicarbonate on biofilms

This testing was done using the XTT assay. The XTT assay is a measure of metabolic activity, and therefore its readout is a proxy measurement for metabolic activity of bacterial cells. Preformed biofilms (treated or untreated with tobramycin and bicarbonate) were treated with XTT and absorbance was measured as the readout. An increase in XTT absorbance can indicate greater metabolic activity and/or more active cells, and a decrease in XTT absorbance can indicates decreased metabolic activity (i.e., bacteria entering a more-inactive state) and/or killing of cells, but cannot differentiate between the two.

### Measurement of the effect of tobramycin+bicarbonate on high density, stationary phase cells

Briefly, a population of *P. aeruginosa* PAO1 cells was grown to an OD=2, to represent stationary phase cells. Different combinations of tobramycin and bicarbonate were made in medium containing salt+water (1% sodium chloride in water) to represent the salinity of standard Luria–Bertani medium, but with no nutrients. This was intended to prevent activation of cell metabolism to planktonic phase as would occur in the presence of medium containing fresh nutrients. Stationary-phase cells (OD=2) were added to these different combinations of tobramycin+bicarbonate in salt water, in a 96-well flat-bottom plate (as used previously). Plates were incubated at 37 °C shaking overnight and OD_600_ values were read after 16–18 h. Owing to the high density of cells, the lowest concentration that inhibited growth corresponded to ~50% inhibition of bacterial growth (reported as MIC_50_). Therefore, MIC_50_ was used for further analysis including isobolograms, FIC plots and response surface.

### Response surface analysis

To construct and analyse response surfaces, we used Mathematica and Google Sheets. Data from checkerboard assays were arranged into arrays (‘PercentChange[T,B]’) giving the measured percent change in optical density (for planktonic cells) or XTT metabolic readout (for biofilms), compared with untreated bacteria. Estimated measurement errors were also arranged into arrays (‘Error[T,B]’) for each checkerboard assay. For each two-dimensional array, the tobramycin concentration was mapped to the first, ‘x’ position index and the bicarbonate concentration was mapped to the second, ‘y’ position index. Arrays were imported into Mathematica.

#### Calculating the surface describing loewe additivity

Loewe additivity is a common reference model to define drug interactions.^25^ According to this model, the combined effect of two drugs is predicted from the sum of the effects of the individual components. For both tobramycin alone and bicarbonate alone, we assumed Hill kinetics of a variable ligand concentration, [L], binding randomly to a finite number of receptors with disassociation constant *K*_d_. The resulting Hill equation has the form

(1)H([L])=[L]mKdm+[L]m

where *m* measures the self-cooperativity of the ligand and the slope of the linear regime of the sigmoidal curve *H*([*L*]). By normalising ligand concentration to the concentration that produces 50% growth inhibition, we remove $K_{d}^{m}$ from the expression, which we can also write in terms of the maximum measured value, *H*_max_, and the minimum measured value, *H*_0_:

(2)H([A])=(Hmax−H0)([A]/A50)m[1+([A]/A50)m]+H0

Here, *H*_0_ corresponds to the absence of any treatment, and *H*_max_ corresponds to the greatest reduction in growth or viability.

We used Mathematica to fit this function to each of the one-dimensional data sets describing the response to increasing concentrations of tobramycin (without bicarbonate) and to increasing concentrations of bicarbonate (without tobramycin), thus:

These fits (Supplementary Figure S4 for planktonic cells and Supplementary Figure S5 for biofilms) were used to determine the concentration that produces 50% growth inhibition for tobramycin alone (*T*_50_) and for bicarbonate alone (*B*_50_), and the *m* values for tobramycin alone (*m*_T_) and for bicarbonate alone Using these parameters, we calculated and plotted the surface-describing Loewe additivity as defined in Meletidias *et al*.^25^ In brief, weighting coefficients are functions of tobramycin concentration [*T*] and bicarbonate concentration [*B*], thus:

(3)WeightTob=([T]T50)/([T]T50+[B]B50)

and

(4)WeightBicarb=([B]B50)/([B]B50+[T]T50).

These weights are used to calculate the local slope of the surface,

(5)mlocal=(WeightTob)mT+(WeightBicarb)(mB),

and to weight the local contributions of each 50% growth inhibition value value,

(6)U0=((T50)(WeightTob))((B50)(WeightBicarb))

The total number of 50% growth inhibition units, also called ‘potency units,’ from both tobramycin and bicarbonate, is given by

(7)U=[T]T50+[B]B50

The Loewe additivity surface is a function of the number of potency units, the local slope, and the weighted local contributions to 50% growth inhibition, thus:

(8)Loewe=(Lower\ Bound−Upper\ Bound)(U/U0)mlocal1+(U/U0)mlocal+Lower\ Bound

where LowerBound is the lowest value measured for growth or viability, and UpperBound is the growth or viability value measured without treatment. Thus, Equation (8) has the same form as the Hill function in Equation (2), but has now been generalised to describe a two-dimensional parameter space.

Our step-by-step approach to calculating the Loewe surface is shown in the annotated Mathematica notebook in Supplementary Material.

The Loewe surface presented a challenge for numerical analysis, as its limits are not well defined as they approach zero. We handled this by redefining our zero values to 0.0001 for Bicarbonate and Tobramycin. The resulting Loewe function was used to populate an array (‘Loewe[T,B]’) describing the response predicted by Loewe additivity.

#### Calculating the surface describing the degree of synergy or antagonism

The departure of the measured response (‘PercentChange’) from that calculated from Loewe additivity theory (‘Loewe’) indicates that the effect of a combination is synergistic or antagonistic, rather than additive. To this difference, we added the measurement errors (‘Error’) to establish an upper bound on the difference surface—i.e., to create a surface describing the upper bound. We then created a surface describing the lower bound by subtracting the measurement errors (‘Error’) from the difference. The calculated difference surfaces were thus given by

Upper\ Difference[T,B]=Loewe[T,B]−PercentChange[T,B]+Error[T,B].

Lower\ Difference[T,B]=Loewe[T,B]−PercentChange[T,B]−Error[T,B].

We interpolated Upper and Lower Difference[T,B] in Mathematica to get an integrable form.

For 4219D biofilms, the response to increasing concentrations of tobramycin alone and of bicarbonate alone was non-monotonic. This prevented fitting of a Hill function, so the response surface was instead estimated as a strictly additive effect of tobramycin and bicarbonate at their respective concentrations with 20% uncertainty at all points.

#### Calculating net synergy or antagonism of a wide range of combinations

We numerically integrated the upper and lower difference surfaces and divided by the projected, planar area under the surfaces to give a quantitative upper and lower bound of synergy, additivity, or antagonism. We took the average of the two area-normalised integrals to give the net measure of tobramycin+bicarbonate interaction, and the difference between the mean and the bounding values as the uncertainty. Our definition of the difference surface means that a value of zero corresponds to no net departure from Loewe additivity, a negative value corresponds to a net synergistic effect, with the magnitude indicating the overall strength of the synergy, and a positive value corresponds to a net antagonistic effect, with the magnitude indicating the overall strength of the antagonism.

A Mathematica notebook giving the code, with explanatory comments, that was used for all parts of this work is included in Supplementary Material.
