# Supplementary material for: Tobramycin and bicarbonate synergise to kill planktonic Pseudomonas aeruginosa, but antagonise to promote biofilm survival
Source: NPJ Biofilms Microbiomes. 2016 May 25;2:16006–. doi: 10.1038/npjbiofilms.2016.6 (PMC5515257; doi:10.1038/npjbiofilms.2016.6)
Supplement: Supplementary Information [file npjbiofilms20166-s1.pdf]

Supplemental Material

Figure Legends

Supplementary Figures

FIG S1 Representative data showing the (A) percent change values and (B) ΣFIC values for the combination of tobramycin and bicarbonate for antibiotic-resistant strain #1. Different concentrations of tobramycin and bicarbonate result in 64 test combinations for each strain. MIC values for each agent were also tested alone (not shown here). In this dataset, MIC values for tobramycin and bicarbonate alone against antibiotic-resistant strain #1 are highlighted in blue. The growth-no growth interface (red boxes) was plotted along lowest combinations producing a 90% growth reduction (A). Below and to the left of this interface, test combination wells showed growth (green boxes). Corresponding ΣFIC values along the growth-no growth interface (B) were calculated using the formula $\Sigma\ \mathrm{FIC_{A+B}} = \mathrm{FIC_A} + \mathrm{FIC_B}$, where $\mathrm{FIC_A} = \frac{\mathrm{MIC_{A+B}}}{\mathrm{MIC_A}}$ and $\mathrm{FIC_B} = \frac{\mathrm{MIC_{A+B}}}{\mathrm{MIC_B}}$, where A and B are the test agents. The ΣFIC index was interpreted as follows: $\Sigma\mathrm{FIC} \leq 0.5$ indicates a synergistic interaction; ≥ 0.5 and < 1 indicates an additive effect; and > 1 indicates antagonism.

FIG S2 Representative graphs showing synergistic (green), additive (orange), and antagonistic (purple) regimes based on (A) concentrations of tobramycin and bicarbonate and (B) ΣFIC values. The ΣFIC index was interpreted as follows: $\Sigma\mathrm{FIC} \leq 0.5$ indicates a synergistic interaction; ≥ 0.5 and < 1 indicates an additive effect; and > 1 indicates antagonism.

FIG S3 Screen for biofilm formation and metabolic activity of biofilm cells for different *P. aeruginosa* strains. (A) Crystal violet assay for biofilm formation by staining biofilm mass (live cells, dead, cells, and extracellular matrix) and (B) XTT assay for metabolic activity in biofilms (proxy for live cells). Error bars represent SEM; N = 3.

FIG S4 Response of planktonic bacteria to treatment by (A,C, E) increasing concentrations of tobramycin alone and (B, D, F) increasing concentrations of bicarbonate alone. The response from (A, B) the lab strain PAO1 and (C-E) clinical isolates from cystic fibrosis patients is monotonically decreasing and well-fit by the Hill function described in Materials and Methods.

FIG S5 Response of biofilm bacteria to treatment by (A,C) increasing concentrations of tobramycin alone and (B,D) increasing concentrations of bicarbonate alone. The response from (A, B) the lab strain PAO1 and (C,D) a clinical isolate from a cystic fibrosis patient is monotonically decreasing and well-fit by the Hill function described in Materials and Methods. Data from clinical isolate strain 4219D is not included in this figure because these trends were not

monotonic and were not well-fit by a Hill function.

FIG S6 Isobologram analyses for tobramycin-bicarbonate combination against planktonic cells of *P. aeruginosa* laboratory strains PA14, and spontaneously generated antibiotic-resistant PA14 mutants #3 and 4. A strong synergistic-additive effect is observed against all three strains. Points along the isobologram represent the growth-no growth interface. The orange shaded area represents the additive region and the green shade area represents the synergistic region.

FIG S7 Isobologram analyses for tobramycin-bicarbonate combination against planktonic cells of spontaneously generated antibiotic-resistant PA14 mutants #1 and 2. A strong synergistic-additive effect is observed against all three strains. Points along the isobologram represent the growth-no growth interface. The orange shaded area represents the additive region and the green shade area represents the synergistic region.

FIG S8 Isobologram analyses for tobramycin-bicarbonate combination against planktonic cells of *P. aeruginosa* clinical cystic fibrosis (CF) mucoid isolates. A strong synergistic-additive effect is observed against all three strains. Points along the isobologram represent the growth-no growth interface. The orange shaded area represents the additive region and the green shade area represents the synergistic region.

FIG S9 Isobologram analyses for tobramycin-bicarbonate combination against planktonic cells of *P. aeruginosa* clinical cystic fibrosis (CF) mucoid isolates. A strong synergistic-additive effect is observed against all three strains. Points along the isobologram represent the growth-no growth interface. The orange shaded area represents the additive region and the green shade area represents the synergistic region.

FIG S10 Isobologram analyses for tobramycin-bicarbonate combination against planktonic cells of *P. aeruginosa* clinical cystic fibrosis (CF) classic and dwarf isolates. A strong synergistic-additive effect is observed against all three strains. Points along the isobologram represent the growth-no growth interface. The orange shaded area represents the additive region and the green shade area represents the synergistic region.

FIG S11 Synergy between fixed combinations of bicarbonate and tobramycin in killing *P. aeruginosa* antibiotic-resistant mutant strains #2-4. Error bars represent SEM; N = 3. For this, the bicarbonate concentration associated with the lowest ΣFIC value for the select strain was plotted against varying tobramycin concentrations tested. For all four strains thus examined, addition of bicarbonate reduces the concentration of tobramycin required to inhibit planktonic *P. aeruginosa* cells even at tobramycin concentrations much lower than that needed to produce synergy.

FIG S12 Synergy between fixed combinations of bicarbonate and tobramycin in killing *P. aeruginosa* clinical cystic fibrosis (CF) mucoid isolates. Error bars represent SEM; N = 3. For this, the bicarbonate concentration associated with the lowest ΣFIC value for the select strain was plotted against varying tobramycin concentrations tested. For all four strains thus examined, addition of bicarbonate reduces the concentration of tobramycin required to inhibit planktonic *P. aeruginosa* cells even at tobramycin concentrations much lower than that needed to produce synergy.

FIG S13 Synergy between fixed combinations of bicarbonate and tobramycin in killing *P. aeruginosa* clinical cystic fibrosis (CF) classic and dwarf isolates. Error bars represent SEM; N = 3. For this, the bicarbonate concentration associated with the lowest ΣFIC value for the select strain was plotted against varying tobramycin concentrations tested. For all four strains thus examined, addition of bicarbonate reduces the concentration of tobramycin required to inhibit planktonic *P. aeruginosa* cells even at tobramycin concentrations much lower than that needed to produce synergy.

FIG S14 Time-kill assays demonstrating the synergy between bicarbonate and tobramycin against *P. aeruginosa* strains PAO1, antibiotic-resistant PA14 mutant #1, and 5913C over 24 hours. Shown here are concentrations of tobramycin and bicarbonate tested alone and in combination, as well as concentration of each agent tested alone at MIC. Concentrations of tobramycin and bicarbonate tested represent the combinations that produced the lowest ΣFIC value. For strain PAO1, 0.25 µg/mL tobramycin and 5 mM bicarbonate; for antibiotic-resistant PA14 mutant #1, 1 µg/mL tobramycin and 5 mM bicarbonate; and for strain 5913C, 1 µg/mL tobramycin and 5 mM bicarbonate were tested alone and in combination. In addition, MIC tobramycin concentrations of 2 µg/mL for PAO1, 8 µg/mL for antibiotic-resistant PA14 mutant #1, and 8 µg/mL for 5913C, and MIC bicarbonate concentrations of 160 mM for PAO1, 160 mM for antibiotic-resistant PA14 mutant #1, and 80 mM for 5913C. Error bars represent SEM. N = 3.

FIG S15 Isobologram analyses for tobramycin-bicarbonate combination against high density, stationary phase cells of *P. aeruginosa* PAO1. An additive effect is observed. MIC50 values were used for analysis. Points along the isobologram represent the growth-no growth interface. The orange shaded area represents the additive region and the green shaded area represents the synergistic region.

FIG S16 Measurements of optical density for PAO1 introduced from stationary-phase, overnight cultures, into different tobramycin + bicarbonate combinations. These growth curves were used to measure the lag time associated with transitioning to exponential growth.

FIG S17 Measurements of optical density for PAO1 introduced from stationary-phase, overnight cultures, into different tobramycin concentrations (with no

bicarbonate present). These growth curves were used to measure the lag time associated with transitioning to exponential growth.

FIG S18 Measurements of optical density for PAO1 introduced from stationary-phase, overnight cultures, into different bicarbonate concentrations (with no tobramycin present). These growth curves were used to measure the lag time associated with transitioning to exponential growth.

FIG S19 Calculated response surfaces assuming Loewe additivity for tobramycin and bicarbonate treatments of (A) PAO1 planktonic bacteria and (B) PAO1 biofilms. Contour lines show increments of 10% change. Tobramycin and bicarbonate concentrations are plotted as the fraction of the concentration that produced 50% inhibition.

FIG S20 Calculated response surfaces assuming Loewe additivity for tobramycin and bicarbonate treatments of (A) 3470C planktonic bacteria and (B) 3470C biofilms. Contour lines show increments of 10% change. Tobramycin and bicarbonate concentrations are plotted as the fraction of the concentration that produced 50% inhibition.

FIG S17. Calculated response surfaces for (A) 4219D planktonic bacteria and (B) 4219D biofilms. (A) For planktonic bacteria, response surfaces were calculated assuming Loewe additivity for tobramycin and bicarbonate treatments. Tobramycin and bicarbonate concentrations are plotted as the fraction of the concentration that produced 50% inhibition. (B) For biofilm bacteria, the non-monotonic response curves on the tobramycin and bicarbonate axes prevented fitting of a Hill function, so the response surface was instead estimated as a strictly-additive effect of tobramycin and bicarbonate at their respective concentrations. Contour lines show increments of 10% change.

FIG S22. Response-surface analyses for tobramycin-bicarbonate combination against high density, stationary-phase cells of *P. aeruginosa* PAO1. A net additive effect is observed. (A) The measured response surface is nearly flat, and very slightly concave-down. This case is intermediate between the strongly concave-up measured response for PAO1 planktonic bacteria (Fig. 5A) and the strongly concave-down measured response for PAO1 biofilm bacteria (Fig. 5B). (B) Ideally-additive surface calculated using the Hill function, as described in the Methods section. (C) The difference surface shows both positive and negative regions. These regions essentially cancel out, as the integral of the difference surface gives a value of -0.353 ± 5.310, so the net effect is nearly purely additive.

TABLE S1 Summary of the efficacy of the Tobramycin-Bicarbonate combination against planktonic cells of *P. aeruginosa.* ARM = antibiotic-resistant mutant.

Strain	MIC Tobramycin (µg/mL)	MIC Bicarbonate (mM)	ΣFIC index (average)	FIC index (range)	Effect
PAO1	2	160	0.37	0.25 – 0.75	Synergistic/Additive
PA14	2	160	0.37	0.25 – 0.75	Synergistic/Additive
ARM #1	8	160	0.49	0.27 - 1	Synergistic/Additive
ARM #2	8	160	0.35	0.15 – 0.63	Synergistic/Additive
ARM #3	8	160	0.45	0.28 – 0.75	Synergistic/Additive
ARM #4	8	160	0.45	0.28 – 0.75	Synergistic/Additive
3639M	1	160	0.48	0.28 – 0.75	Synergistic/Additive
4278M	1	160	0.48	0.28 – 0.75	Synergistic/Additive
5623M	8	80	0.52	0.31 – 0.75	Synergistic/Additive
5914M	8	80	0.28	0.18 – 0.5	Synergistic/Additive
0476M	1	80	0.48	0.31 – 0.63	Synergistic/Additive
4220M	2	160	0.49	0.28 – 0.75	Synergistic/Additive
2159M	2	160	0.47	0.28 – 0.63	Synergistic/Additive
5913C	8	80	0.67	0.3 – 1	Synergistic/Additive
3470C	1	160	0.45	0.28 – 0.63	Synergistic/Additive
1913C	2	160	0.57	0.53 - 0.63	Additive
3488D	4	80	0.51	0.31 – 0.75	Synergistic/Additive
4219D	2	160	0.21	0.04 - 0.53	Synergistic/Additive

TABLE S2 Corresponding pH values for different concentrations of bicarbonate in Luria-Bertani (LB) medium. SEM = standard error of the mean. LB = Luria-Bertani

Concentration of Bicarbonate (mM)	Corresponding pH values (± SEM) measured in LB medium
640	8.32 ± 0.02
320	8.14 ± 0.01
160	8.06 ± 0.02
80	7.97 ± 0.03
40	7.69 ± 0.01
20	7.51 ± 0.01
10	7.35 ± 0.02
5	7.12 ± 0.07
2.5	7.03 ± 0.03
1.25	6.97 ± 0.03
0.625	6.94 ± 0.03
0	6.9 ± 0.08

TABLE S3 Efficacy of the Tobramycin-Bicarbonate combination against biofilm cells of *P. aeruginosa*.

**Strains**	***MBIC Tobramycin (μg/mL)**	**#MBIC Bicarbonate (mM)**	**ΣFIC index (average)**	**ΣFIC index (range)**	**Effect**
PAO1	2	320	0.65	0.56 – 0.75	Additive
3470C	2	320	1.2	1 – 1.5	Additive/Antagonistic
4219D	2	320	1.1	1 – 1.5	Additive/Antagonistic

* For strains PAO1 and 3470, a maximum of 80-fold reduction, and for strain 4219D, a maximum of 55-fold reduction was achievable at this concentration.

# For strains PAO1 and 3470, a maximum of 80-fold reduction, and for strain 4219D, a maximum of 60-fold reduction was achievable at this concentration.

Figure S1

A

Percent change values — **Tobramycin (µg/mL)**

Bicarbonate (mM)	0.25	0.5	1	2	4	8	16	32
**160**	-93	-93	-93	-93	-93	-93	-93	-93
**80**	-87	-89	-93	-93	-93	-93	-93	-93
**40**	-86	-89	-91	-93	-93	-93	-93	-93
**20**	-87	-90	-93	-93	-93	-93	-93	-93
**10**	-85	-90	-93	-93	-93	-91	-93	-92
**5**	-78	-82	-93	-93	-93	-93	-93	-93
**2.5**	-70	-76	-82	-93	-93	-93	-93	-93
**1.25**	-67	-75	-78	-90	-93	-93	-93	-93

B

ΣFIC values — **Tobramycin (µg/mL)**

Bicarbonate (mM)	0.25	0.5	1	2	4	8	16	32
**160**	1.03	1.06	1.13	1.25	1.50	2.00	3.00	5.00
**80**	0.53	0.56	0.63	0.75	1.00	1.50	2.50	4.50
**40**	0.28	0.31	0.38	0.50	0.75	1.25	2.25	4.25
**20**		0.19	0.25	0.38	0.63	1.13	2.13	4.13
**10**		0.13	0.19	0.31	0.56	1.06	2.06	4.06
**5**			0.16	0.28	0.53	1.03	2.03	4.03
**2.5**				0.27	0.52	1.02	2.02	4.02
**1.25**				0.26	0.51	1.01	2.01	4.01

FIG S1 Representative data showing the (A) percent change values and (B) ΣFIC values for the combination of tobramycin and bicarbonate for antibiotic-resistant strain #1. Different concentrations of tobramycin and bicarbonate result in 64 test combinations for each strain. MIC_{90} values for each agent were also tested alone (not shown here). In this dataset, MIC_{90} values for tobramycin and bicarbonate alone against antibiotic-resistant strain #1 are highlighted in blue. The growth-no growth interface (red boxes) was plotted along lowest combinations producing a 90% growth reduction (A). Below and to the left of this interface, test combination wells showed growth (green boxes). Corresponding ΣFIC values along the growth-no growth interface (B) were calculated using the formula $\Sigma\ FIC_{A+B}=FIC_A+FIC_B$, where $FIC_A = MIC_{A+B}/MIC_A$ and $FIC_B=MIC_{A+B}/MIC_B$, where A and B are the test agents. The ΣFIC index was interpreted as follows: ΣFIC ≤ 0.5 indicates a synergistic interaction; ≥ 0.5 and < 1 indicates an additive effect; and > 1 indicates antagonism.

Figure S2

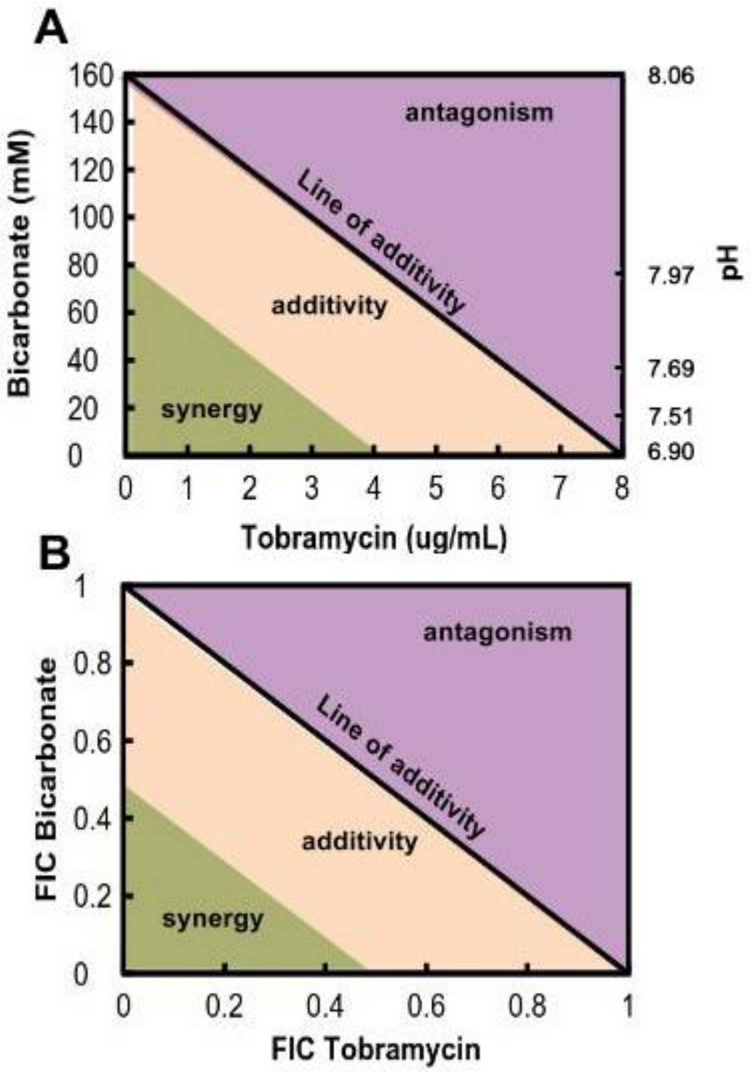

FIG S2 Representative graphs showing synergistic (green), additive (orange), and antagonistic (purple) regimes based on (A) concentrations of tobramycin and bicarbonate and (B) ΣFIC values.

Figure S3

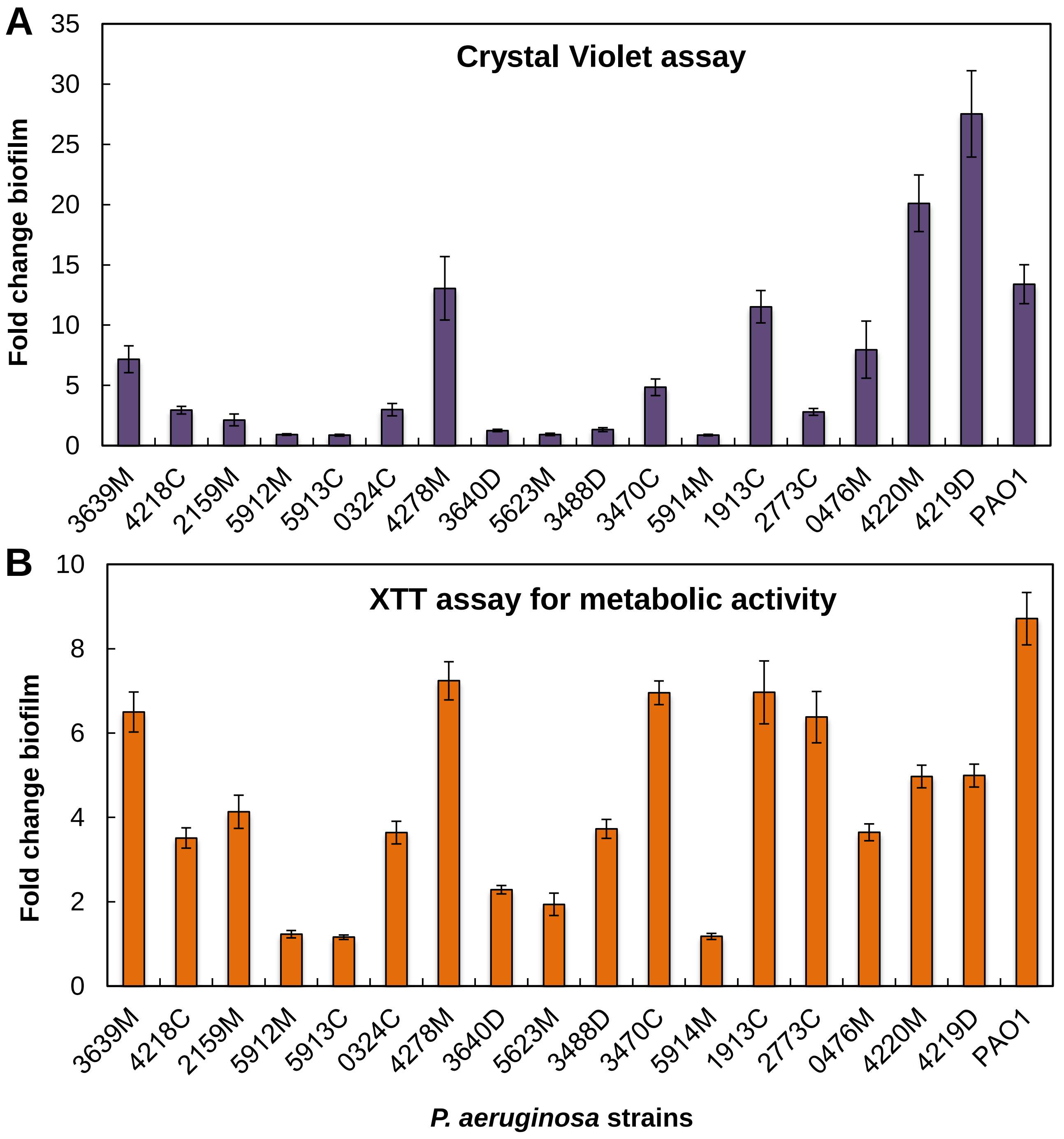

FIG S3 Screen for biofilm formation and metabolic activity of biofilm cells for different *P. aeruginosa* strains. (A) Crystal violet assay for biofilm formation by staining biofilm mass (live cells, dead, cells, and extracellular matrix) and (B) XTT assay for metabolic activity in biofilms (proxy for live cells). Error bars represent SEM; N = 3.

Figure S4

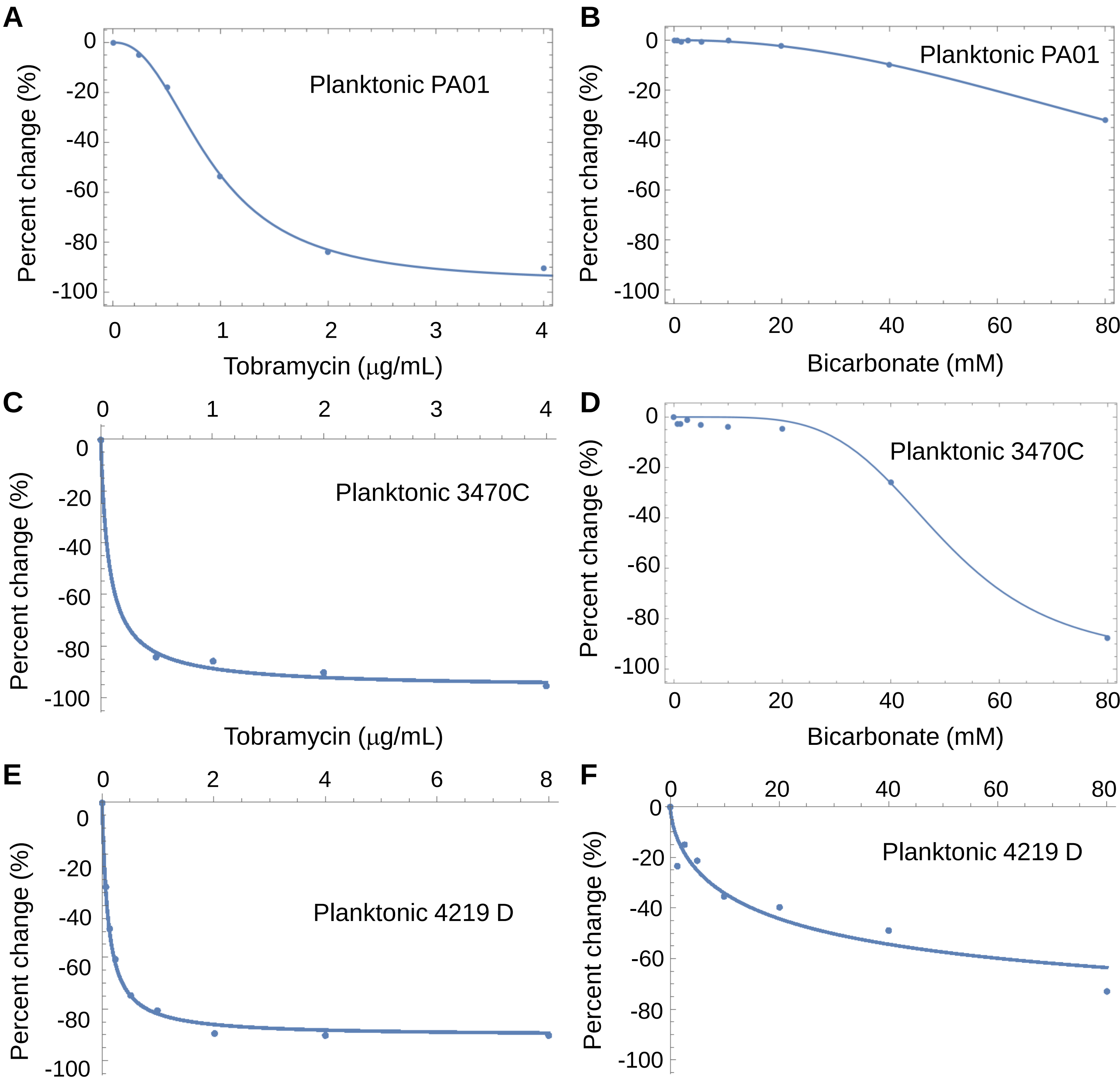

FIG S4 Response of planktonic bacteria to treatment by (A,C, E) increasing concentrations of tobramycin alone and (B, D, F) increasing concentrations of bicarbonate alone. The response from (A, B) the lab strain PA01 and (C-E) clinical isolates from cystic fibrosis patients is monotonically decreasing and well-fit by the Hill function described in Materials and Methods.

Figure S5

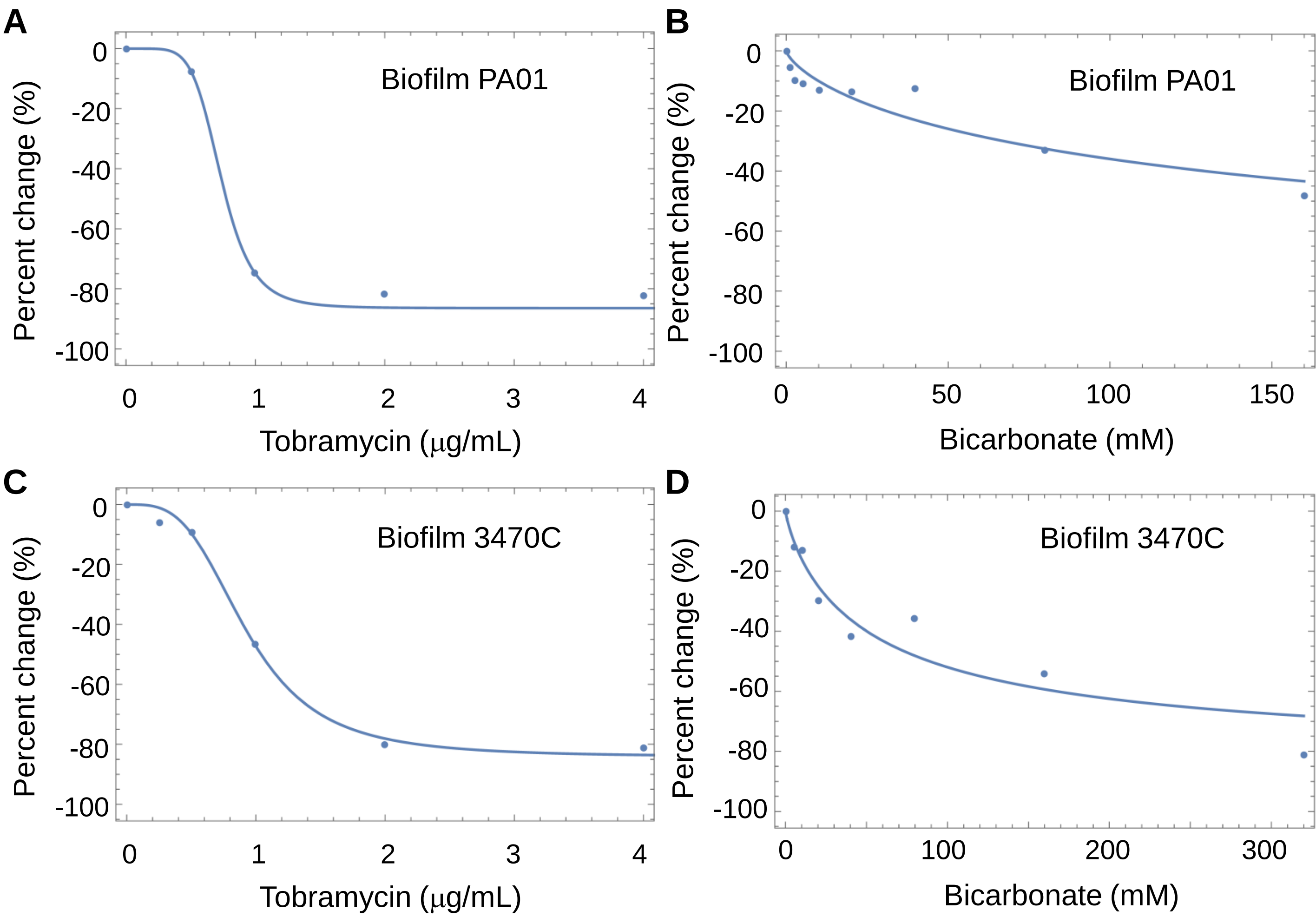

FIG S5 Response of biofilm bacteria to treatment by (A,C) increasing concentrations of tobramycin alone and (B,D) increasing concentrations of bicarbonate alone. The response from (A, B) the lab strain PA01 and (C,D) a clinical isolate from a cystic fibrosis patient is monotonically decreasing and well-fit by the Hill function described in Materials and Methods. Data from clinical isolate strain 4219D is not included in this figure because these trends were not monotonic and were not well-fit by a Hill function.

Figure S6

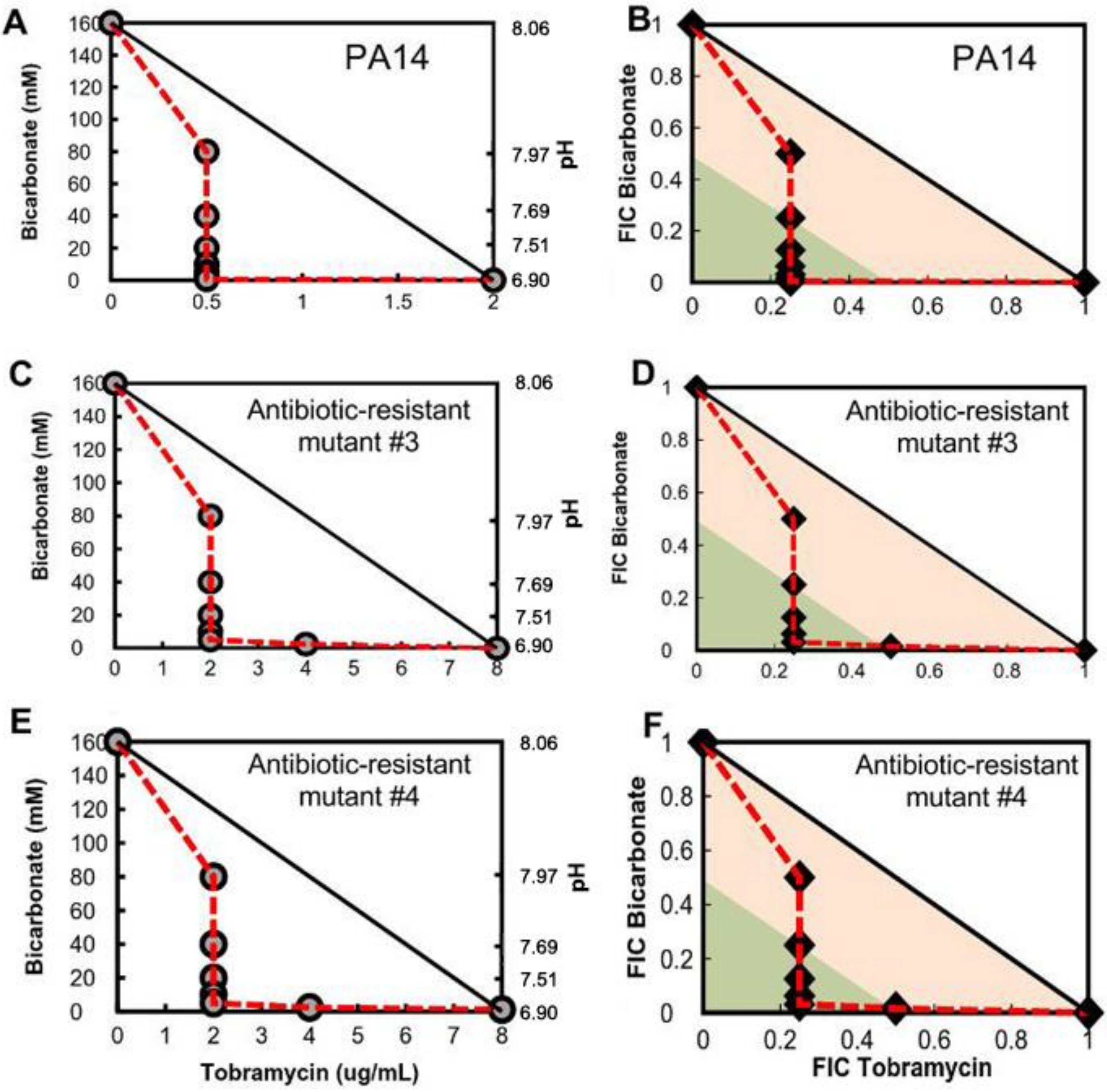

FIG S6 Isobologram analyses for tobramycin-bicarbonate combination against planktonic cells of *P. aeruginosa* laboratory strains PA14, and spontaneously generated antibiotic-resistant PA14 mutants #3 and 4. A strong synergistic-additive effect is observed against all three strains. Points along the isobologram represent the growth-no growth interface. The orange shaded area represents the additive region and the green shade area represents the synergistic region.

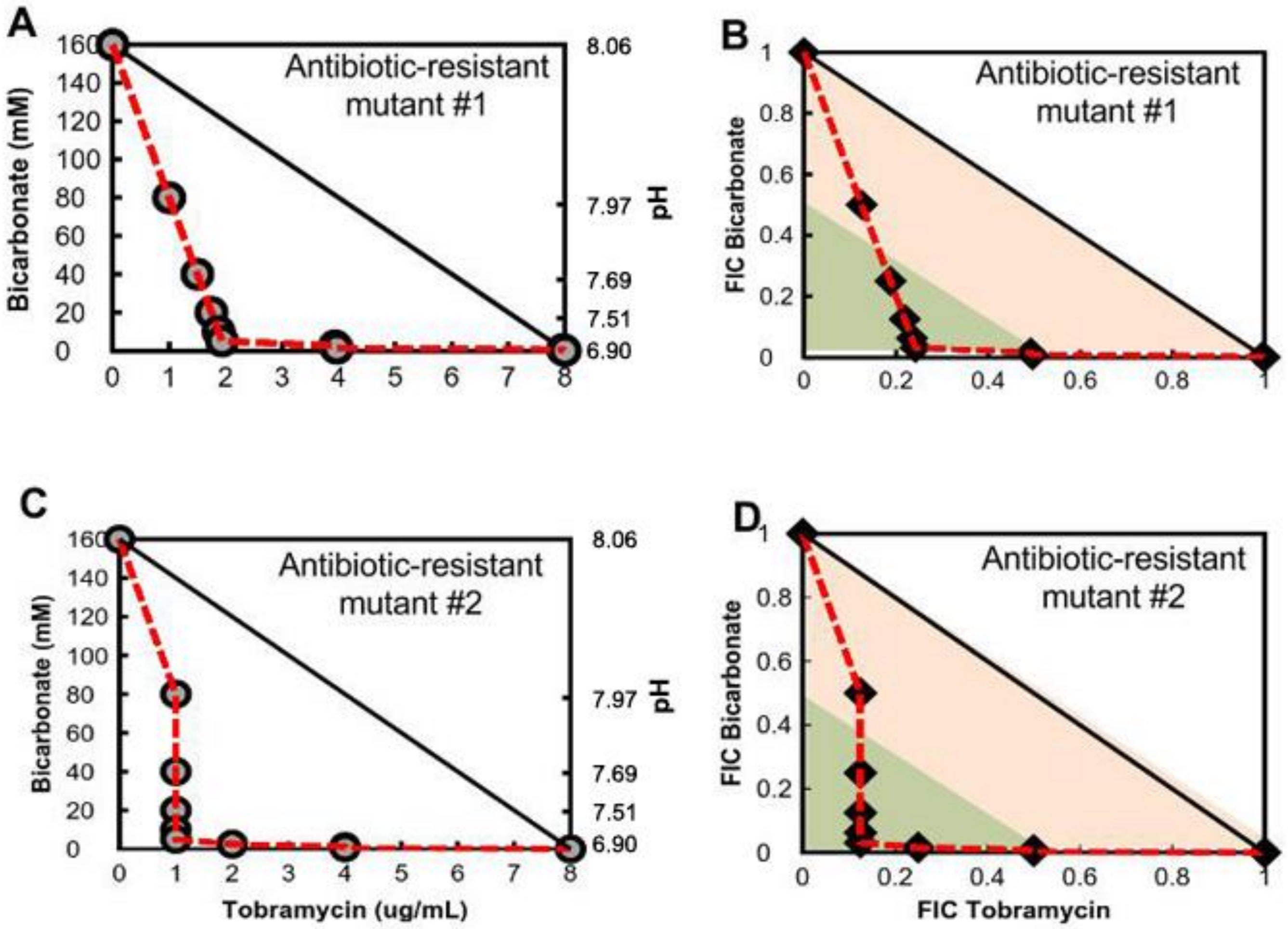

FIG S7 Isobologram analyses for tobramycin-bicarbonate combination against planktonic cells of *P*spontaneously generated antibiotic-resistant PA14 mutants #1 and 2. A strong synergistic-additive effect is observed against all three strains. Points along the isobologram represent the growth-no growth interface. The orange shaded area represents the additive region and the green shade area represents the synergistic region.

Figure S8

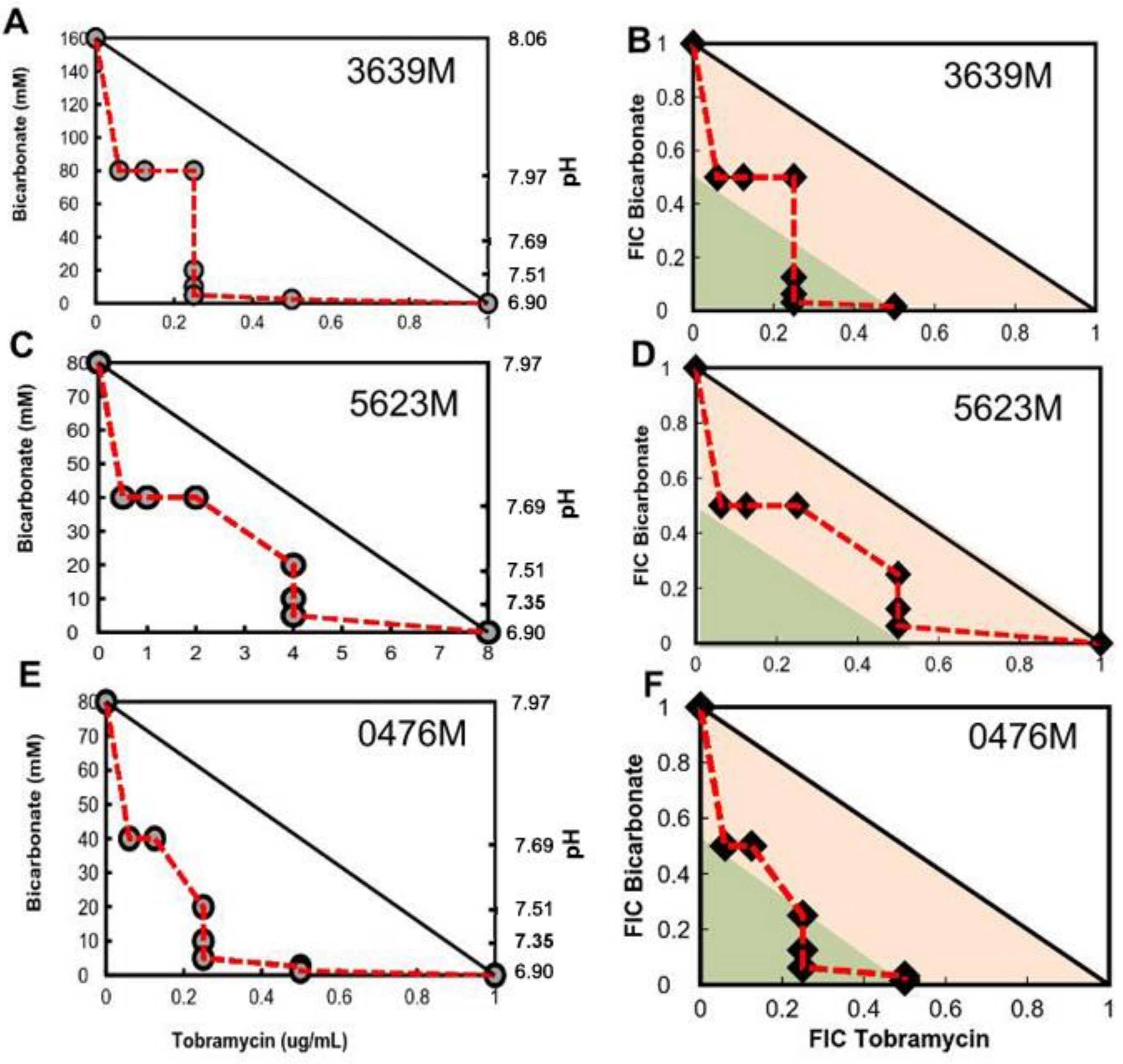

FIG S8 Isobologram analyses for tobramycin-bicarbonate combination against planktonic cells of *P. aeruginosa* clinical cystic fibrosis (CF) mucoid isolates. A strong synergistic-additive effect is observed against all three strains. Points along the isobologram represent the growth-no growth interface. The orange shaded area represents the additive region and the green shade area represents the synergistic region.

Figure S9

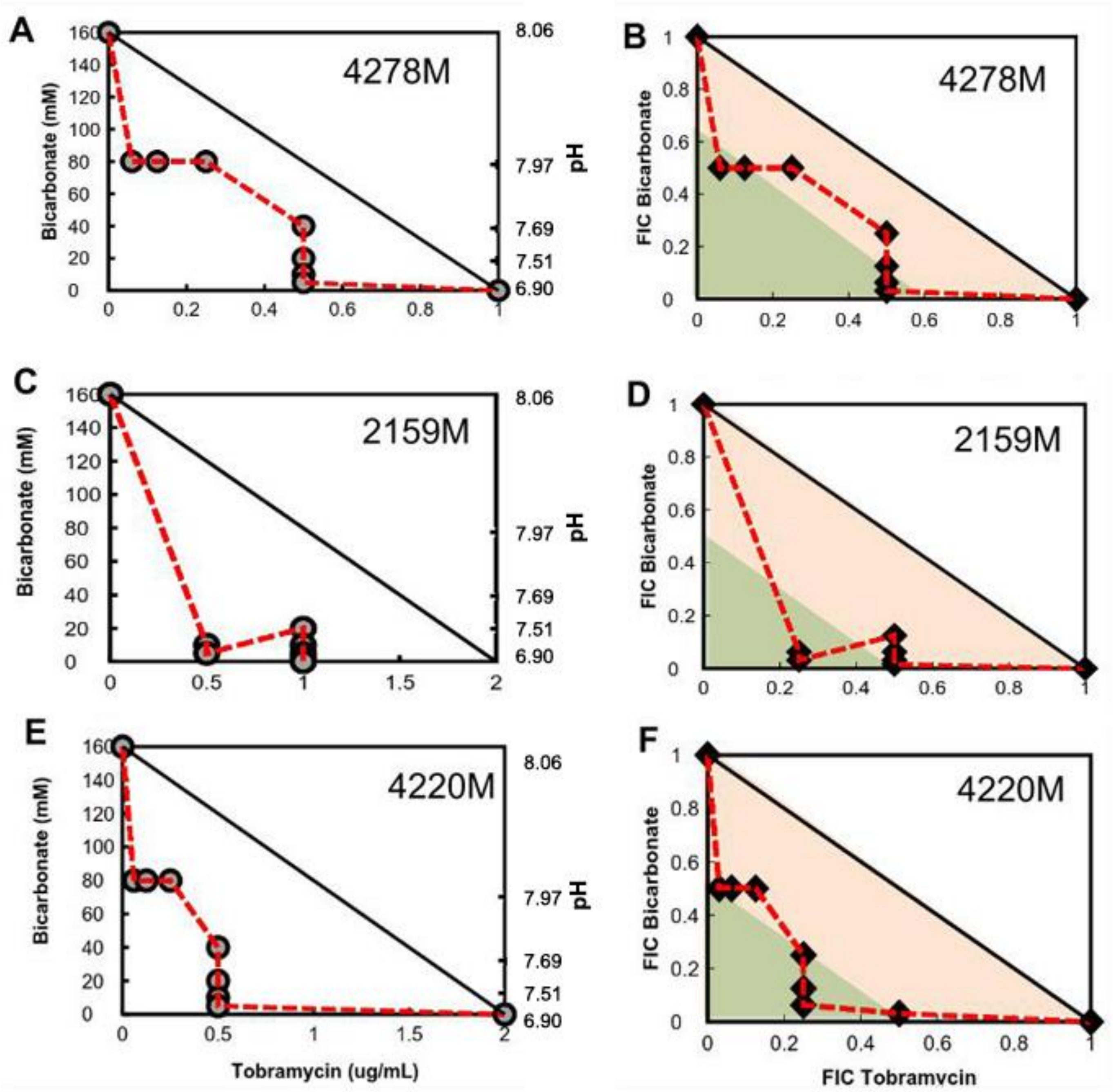

FIG S9 Isobologram analyses for tobramycin-bicarbonate combination against planktonic cells of *P. aeruginosa* clinical cystic fibrosis (CF) mucoid isolates. A strong synergistic-additive effect is observed against all three strains. Points along the isobologram represent the growth-no growth interface. The orange shaded area represents the additive region and the green shade area represents the synergistic region.

Figure S10

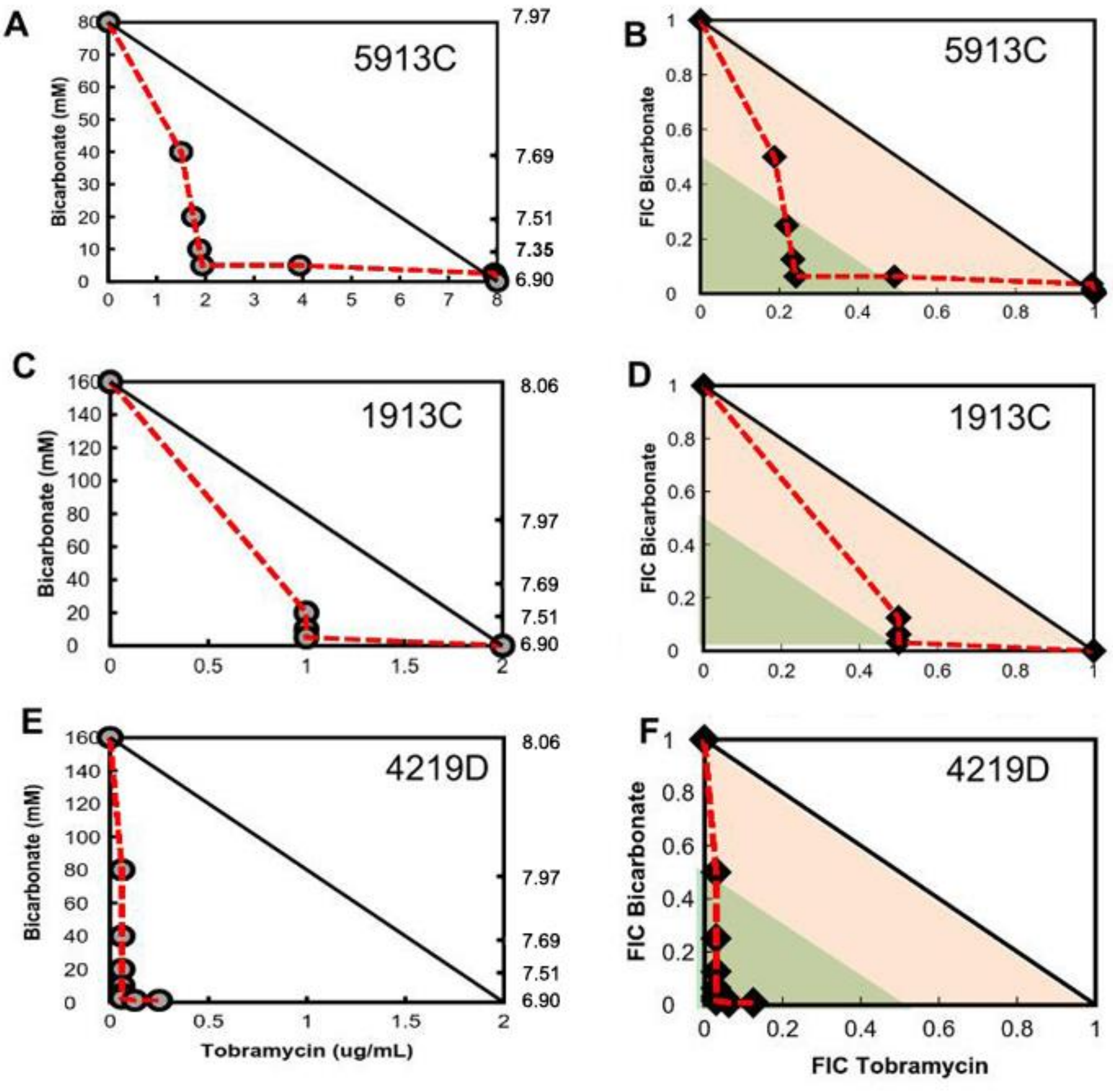

FIG S10 Isobologram analyses for tobramycin-bicarbonate combination against planktonic cells of *P. aeruginosa* clinical cystic fibrosis (CF) classic and dwarf isolates. A strong synergistic-additive effect is observed against all three strains. Points along the isobologram represent the growth-no growth interface. The orange shaded area represents the additive region and the green shade area represents the synergistic region.

Figure S11

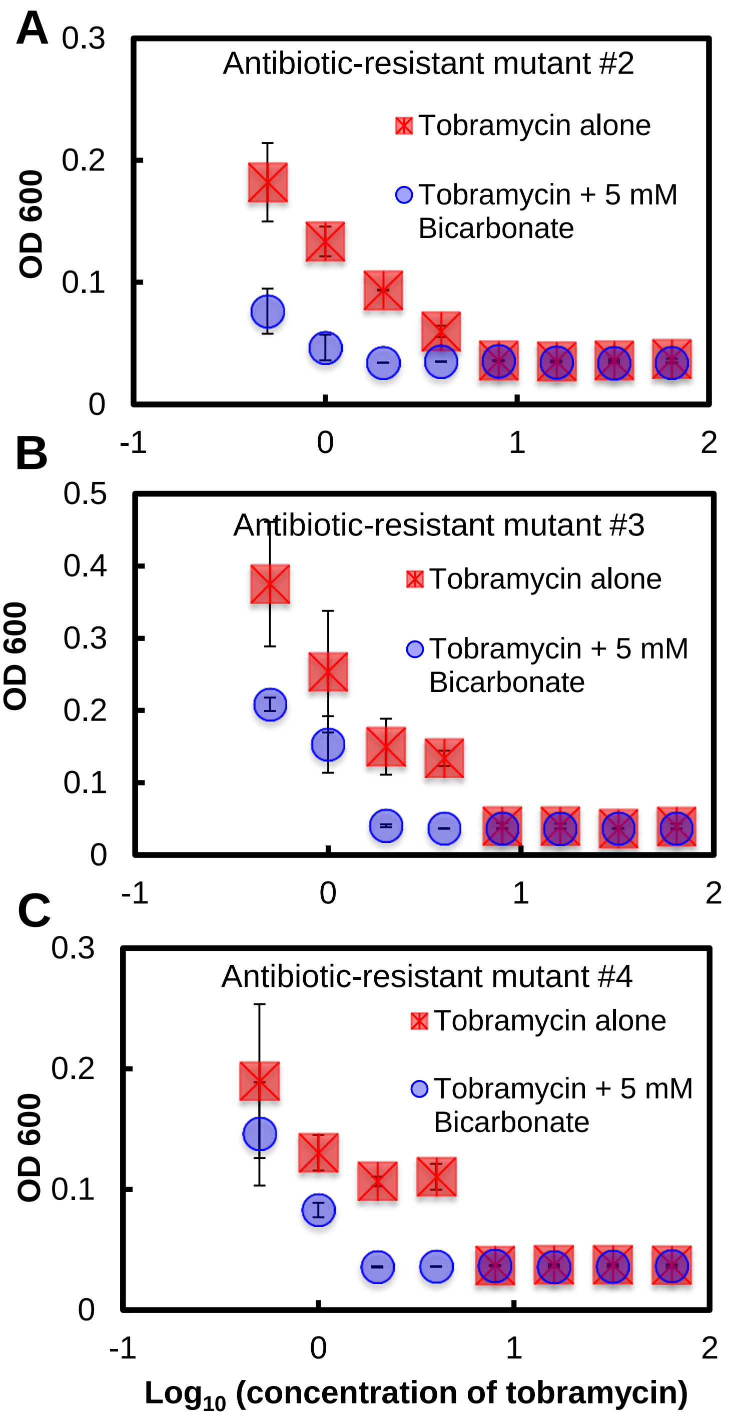

FIG S11 Synergy between fixed combinations of bicarbonate and tobramycin in killing *P. aeruginosa* antibiotic-resistant mutant strains #2-4. Error bars represent SEM; N = 3. For this, the bicarbonate concentration associated with the lowest ΣFIC value for the select strain was plotted against varying tobramycin concentrations tested. For all four strains thus examined, addition of bicarbonate reduces the concentration of tobramycin required to inhibit planktonic *P. aeruginosa* cells even at tobramycin concentrations much lower than that needed to produce synergy.

Figure S12

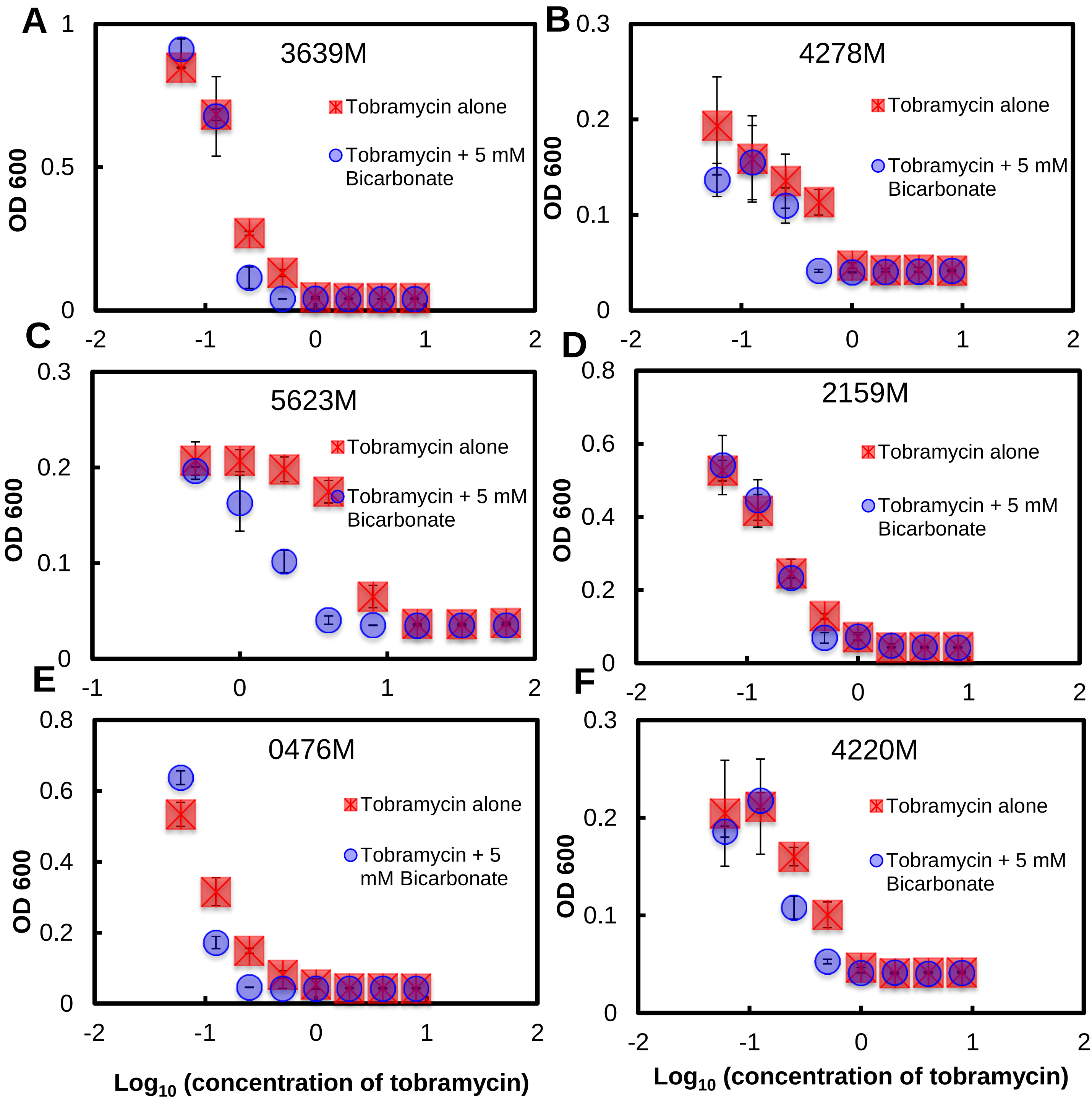

FIG S12 Synergy between fixed combinations of bicarbonate and tobramycin in killing *P. aeruginosa* clinical cystic fibrosis (CF) mucoid isolates. Error bars represent SEM; N = 3. For this, the bicarbonate concentration associated with the lowest ΣFIC value for the select strain was plotted against varying tobramycin concentrations tested. For all four strains thus examined, addition of bicarbonate reduces the concentration of tobramycin required to inhibit planktonic *P. aeruginosa* cells even at tobramycin concentrations much lower than that needed to produce synergy.

Figure S13

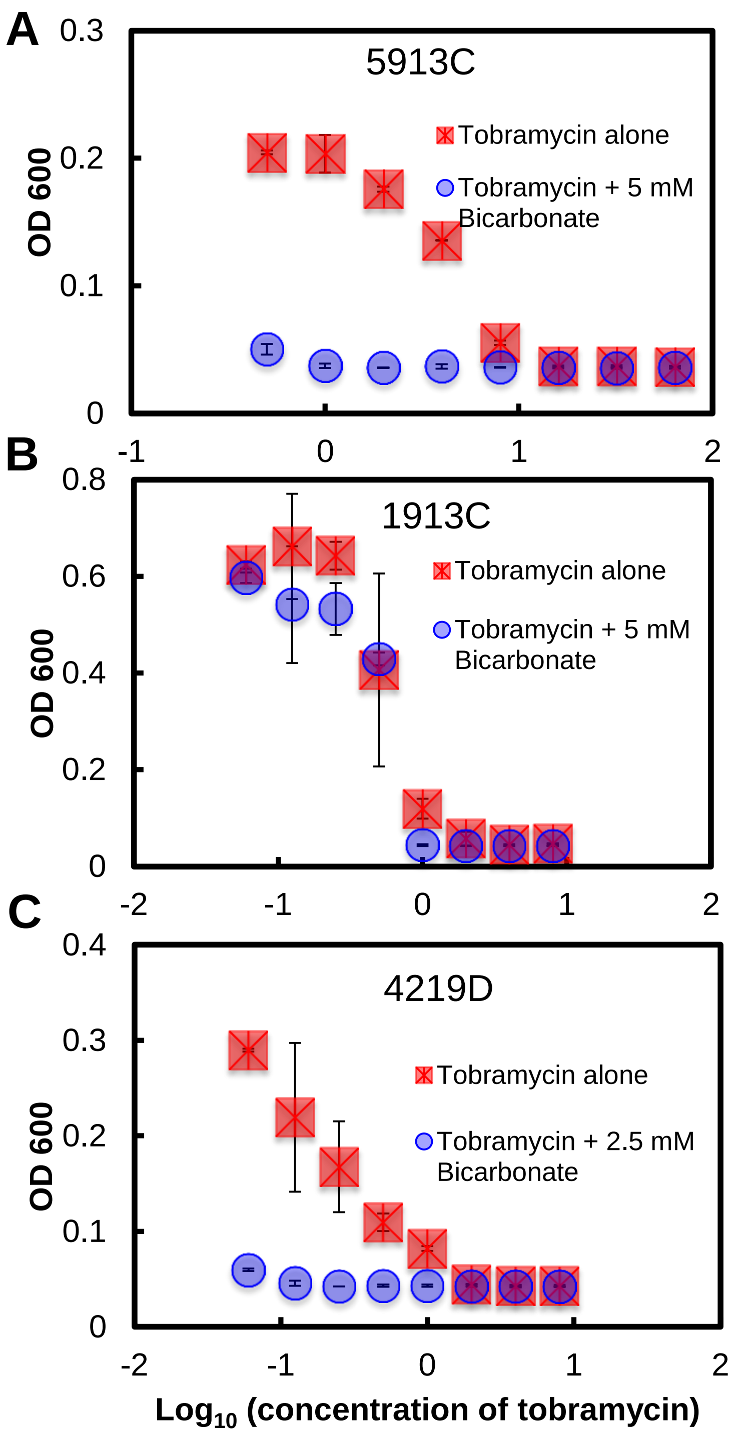

FIG S13 Synergy between fixed combinations of bicarbonate and tobramycin in killing *P. aeruginosa* clinical cystic fibrosis (CF) classic and dwarf isolates. Error bars represent SEM; N = 3. For this, the bicarbonate concentration associated with the lowest ΣFIC value for the select strain was plotted against varying tobramycin concentrations tested. For all four strains thus examined, addition of bicarbonate reduces the concentration of tobramycin required to inhibit planktonic *P. aeruginosa* cells even at tobramycin concentrations much lower than that needed to produce synergy.

Figure S14

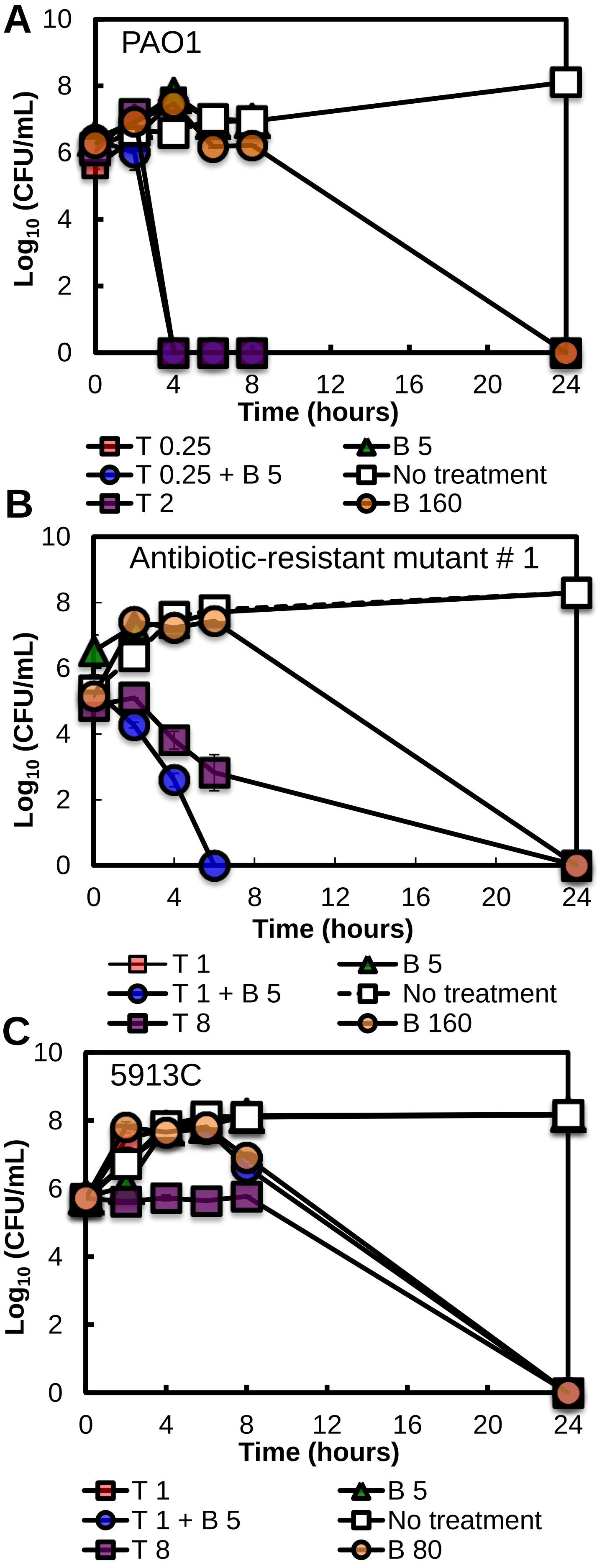

FIG S14 Time-kill assays demonstrating the synergy between bicarbonate and tobramycin against *P. aeruginosa* strains PAO1, antibiotic-resistant PA14 mutant #1, and 5913C over 24 hours. Shown here are concentrations of tobramycin and bicarbonate tested alone and in combination, as well as concentration of each agent tested alone at MIC_{90}. Concentrations of tobramycin and bicarbonate tested represent the combinations that produced the lowest ΣFIC value. For strain PAO1, 0.25 μg/mL tobramycin and 5 mM bicarbonate; for antibiotic-resistant PA14 mutant #1, 1 μg/mL tobramycin and 5 mM bicarbonate; and for strain 5913C, 1 μg/mL tobramycin and 5 mM bicarbonate were tested alone and in combination. In addition, MIC_{90} tobramycin concentrations of 2 μg/mL for PAO1, 8 μg/mL for antibiotic-resistant PA14 mutant #1, and 8 μg/mL for 5913C, and MIC_{90} bicarbonate concentrations of 160 mM for PAO1, 160 mM for antibiotic-resistant PA14 mutant #1, and 80 mM for 5913C. Error bars represent SEM. N = 3.

Figure S15

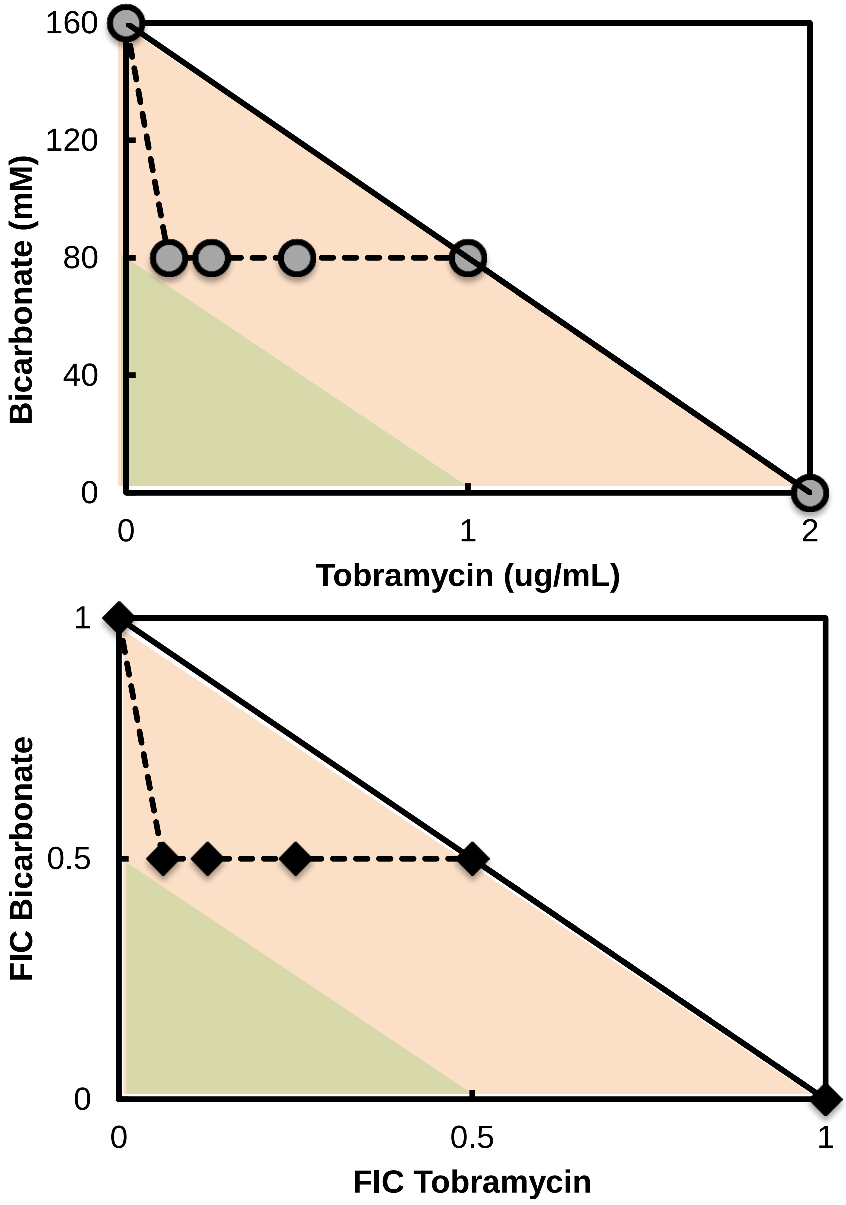

FIG S15 Isobologram analyses for tobramycin-bicarbonate combination against high density, stationary phase cells of *P. aeruginosa* PAO1. An additive effect is observed. MIC50 values were used for analysis. Points along the isobologram represent the growth-no growth interface. The orange shaded area represents the additive region and the green shaded area represents the synergistic region.

Figure S16

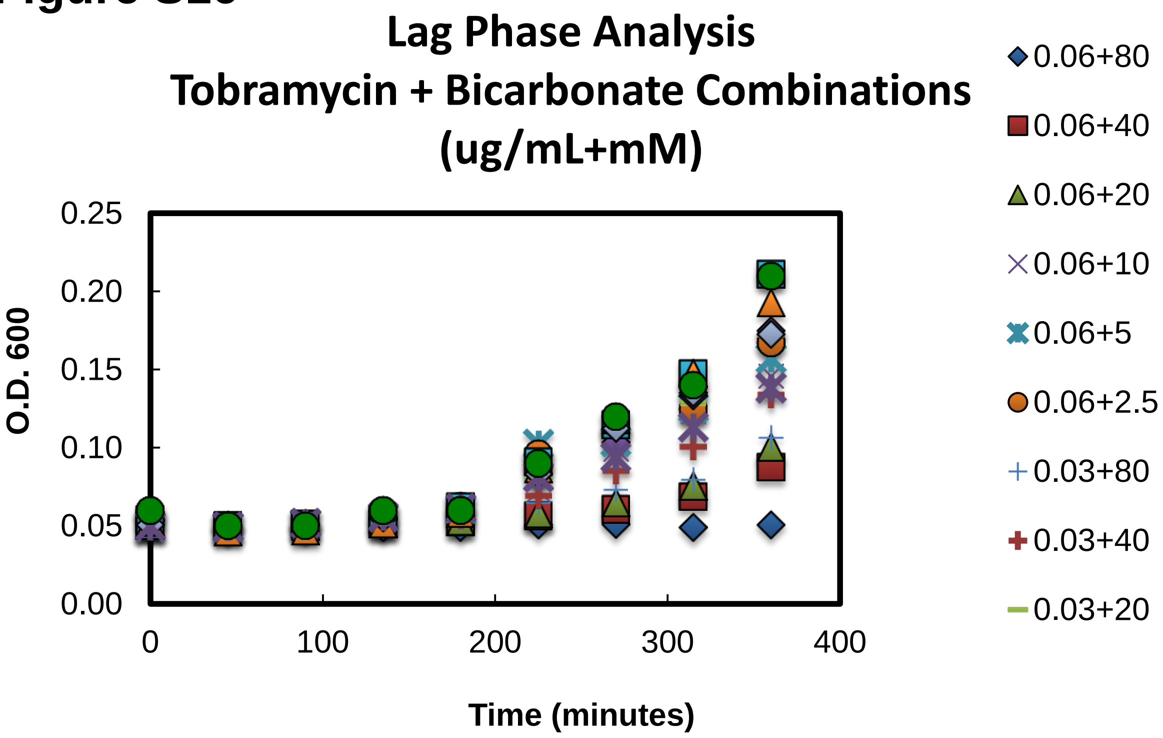

FIG S16. Measurements of optical density for PAO1 introduced from stationary-phase, overnight cultures, into different tobramycin + bicarbonate combinations. These growth curves were used to measure the lag time associated with transitioning to exponential growth.

Figure S17

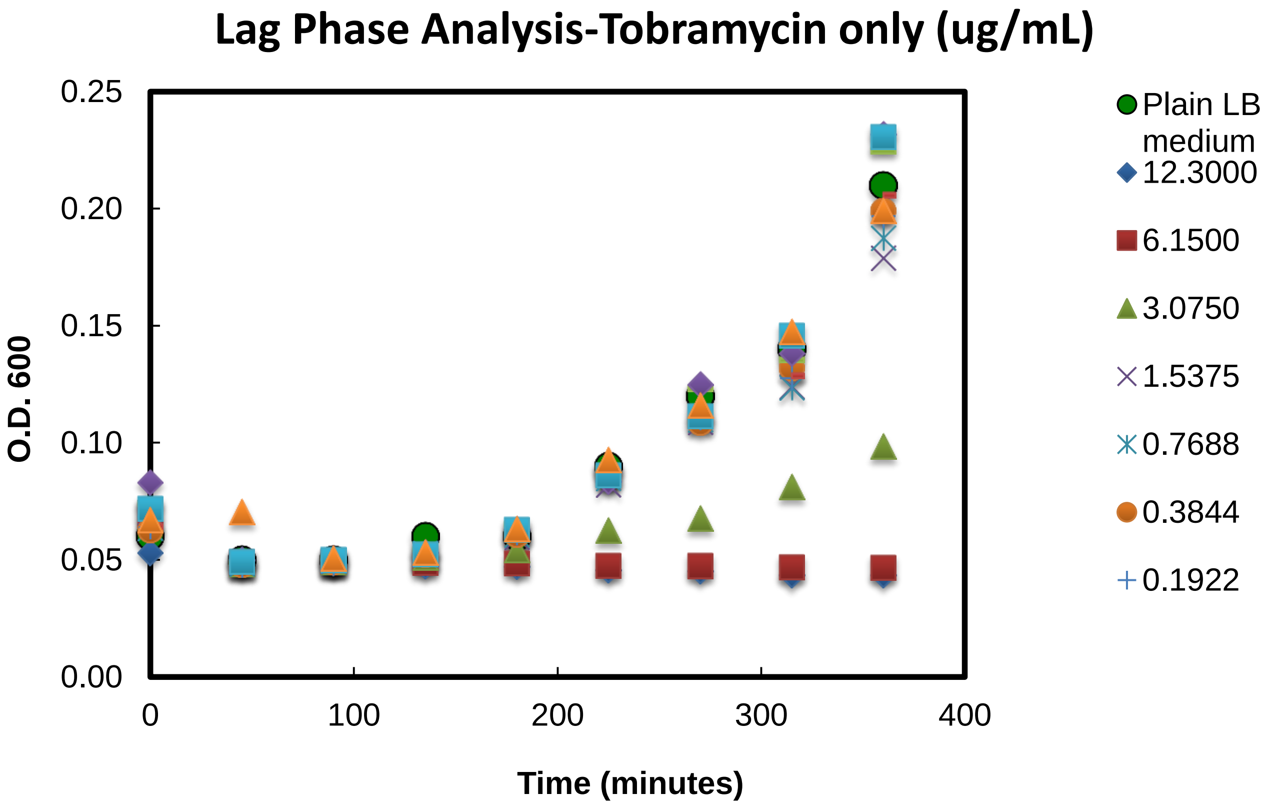

FIG S17. Measurements of optical density for PAO1 introduced from stationary-phase, overnight cultures, into different tobramycin concentrations (with no bicarbonate present). These growth curves were used to measure the lag time associated with transitioning to exponential growth.

Figure S18

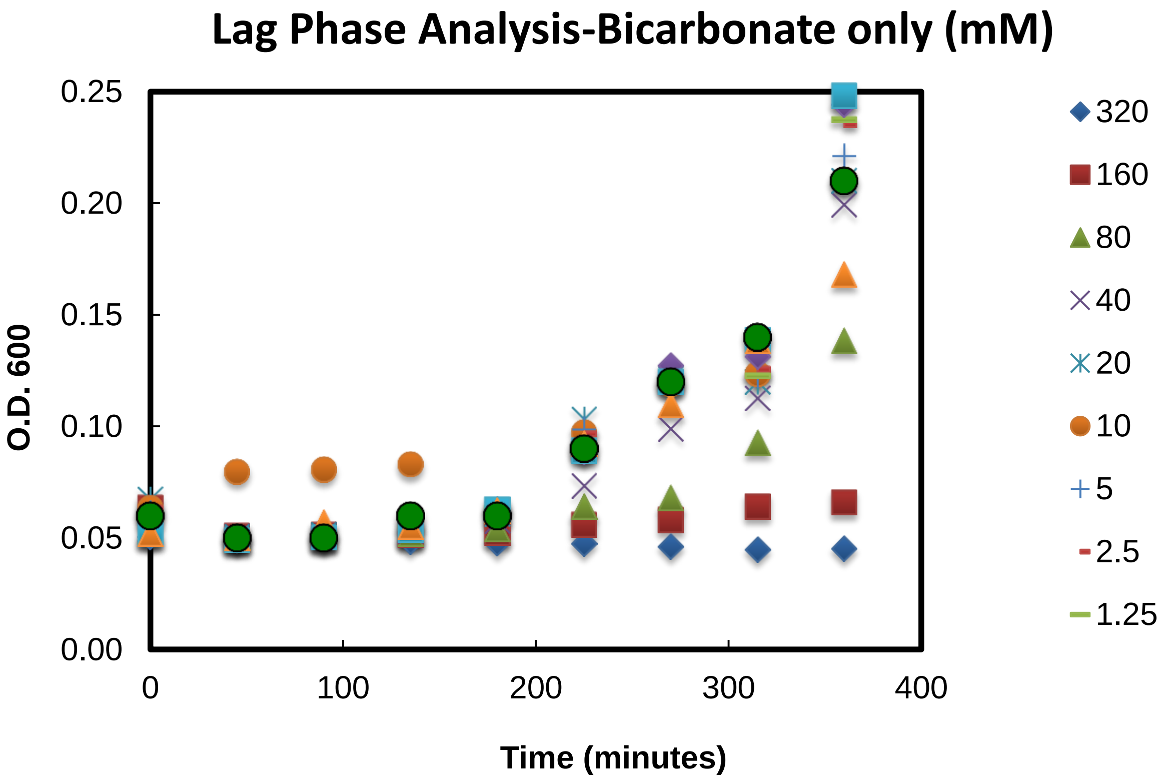

FIG S18. Measurements of optical density for PAO1 introduced from stationary-phase, overnight cultures, into different bicarbonate concentrations (with no tobramycin present). These growth curves were used to measure the lag time associated with transitioning to exponential growth.

Figure S19

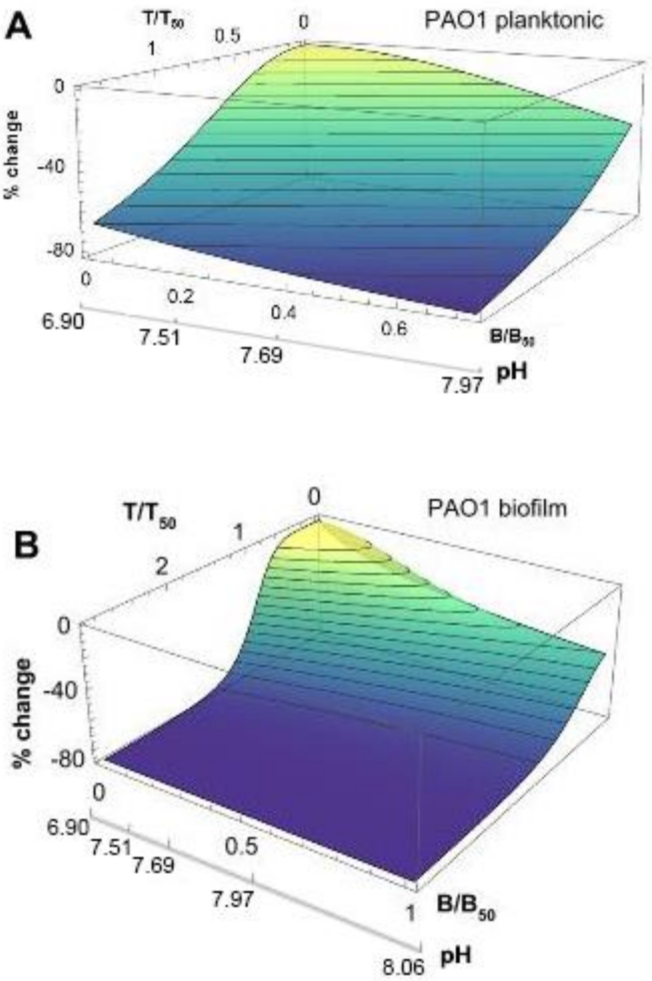

FIG S19. Calculated response surfaces assuming Loewe additivity for tobramycin and bicarbonate treatments of (A) PA01 planktonic bacteria and (B) PA01 biofilms. Contour lines show increments of 10% change. Tobramycin and bicarbonate concentrations are plotted as the fraction of the MIC_{50}.

Figure S20

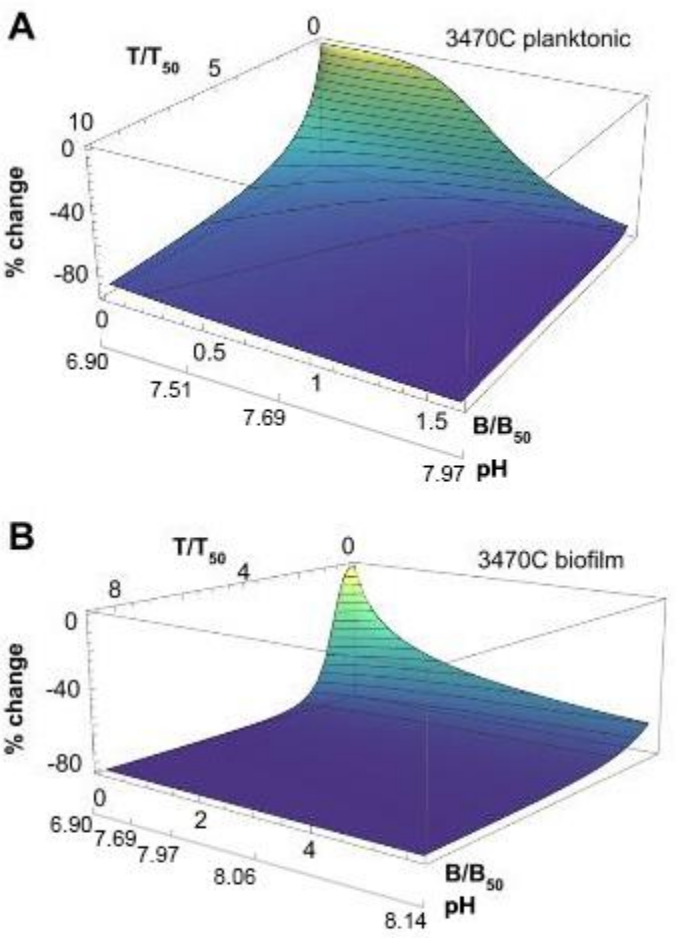

FIG S20. Calculated response surfaces assuming Loewe additivity for tobramycin and bicarbonate treatments of (A) 3470C planktonic bacteria and (B) 3470C biofilms. Contour lines show increments of 10% change. Tobramycin and bicarbonate concentrations are plotted as the fraction of the MIC_{50}.

Figure S21

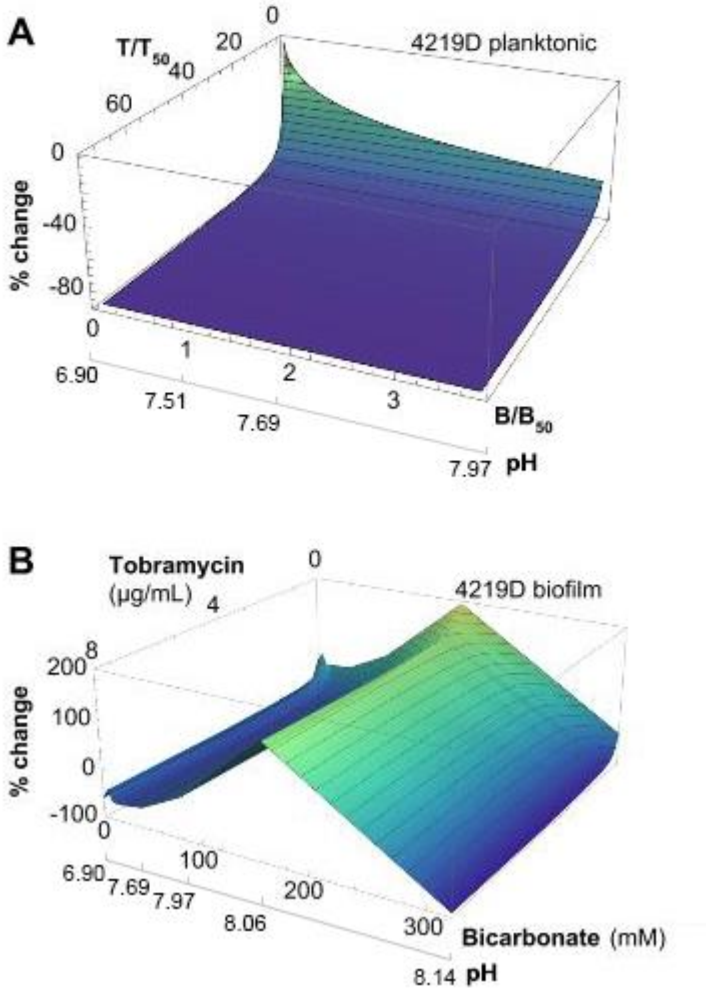

FIG S21. Calculated response surfaces for (A) 4219D planktonic bacteria and (B) 4219D biofilms. (A) For planktonic bacteria, response surfaces were calculated assuming Loewe additivity for tobramycin and bicarbonate treatments. Tobramycin and bicarbonate concentrations are plotted as the fraction of the MIC_{50}. (B) For biofilm bacteria, the non-monotonic response curves on the tobramycin and bicarbonate axes prevented fitting of a Hill function, so the response surface was instead estimated as a strictly-additive effect of tobramycin and bicarbonate at their respective concentrations. Contour lines show increments of 10% change.

Figure S22

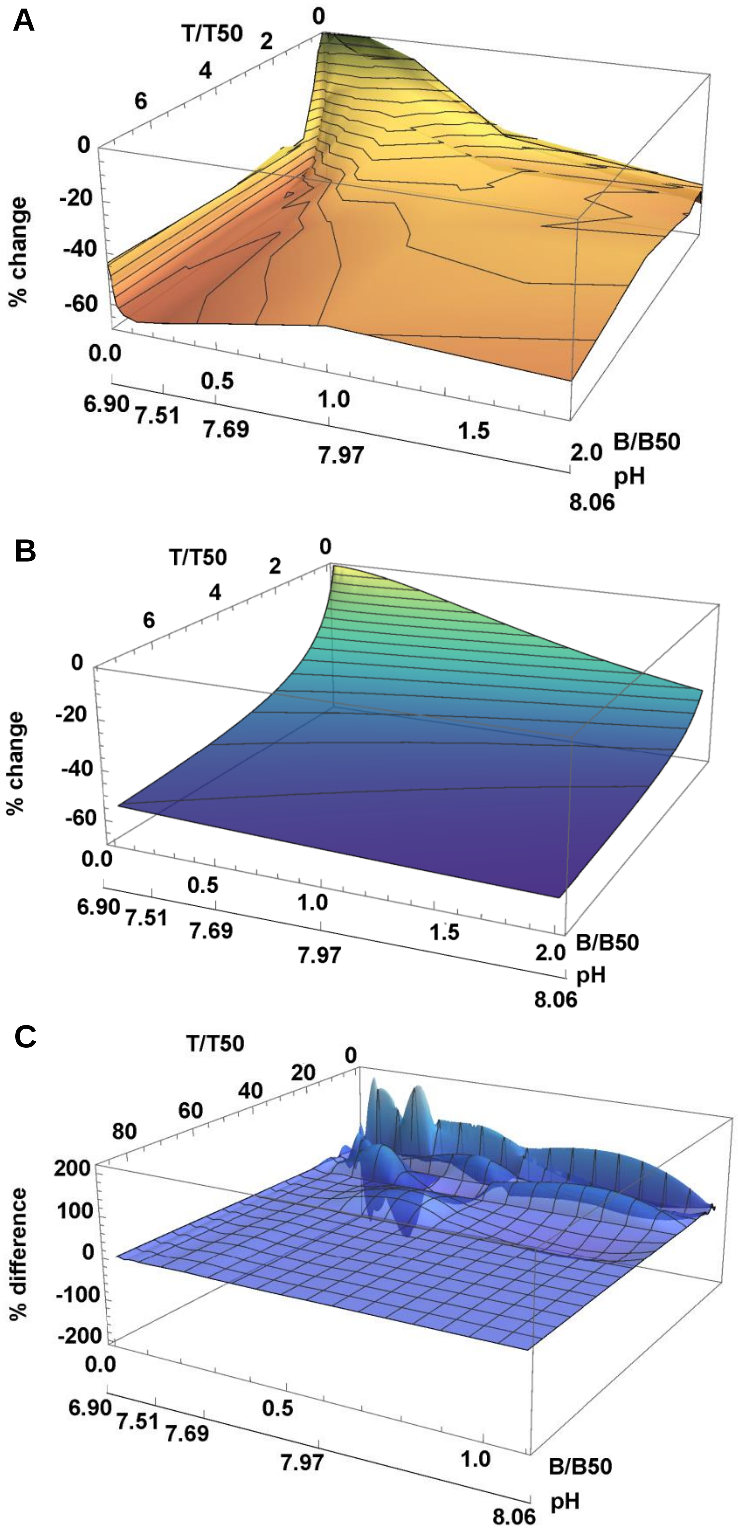

FIG S22 Response-surface analyses for tobramycin-bicarbonate combination against high density, stationary-phase cells of *P. aeruginosa* PAO1. A net additive effect is observed. (A) The measured response surface is nearly flat, and very slightly concave-down. This case is intermediate between the strongly concave-up measured response for PAO1 planktonic bacteria (Fig. 5A) and the strongly concave-down measured response for PAO1 biofilm bacteria (Fig. 5B). (B) Ideally-additive surface calculated using the Hill function, as described in the Methods section. (C) The difference surface shows both positive and negative regions. These regions essentially cancel out, as the integral of the difference surface gives a value of -0.353 ± 5.310, so the net effect is nearly purely additive.

```
HillTo[t_] := (Z) * (t / t50) ^mt / (1 + (t / t50) ^mt);

HillBo[b_] := (Z) * (b / b50) ^mb / (1 + (b / b50) ^mb);

X[x_, y_] := (x / x50) / ((x / x50 + y / y50));

Y[x_, y_] := (y / y50) / ((x / x50 + y / y50));

M[x_, y_] := mx * X[x, y] + my * Y[x, y];

Uo[x_, y_] := x50^X[x, y] * my^Y[x, y];

U[x_, y_] := x / x50 + y / y50;

L[x_, y_] :=
  ((Lf - Lo)) (U[x, y] / Uo[x, y]) ^M[x, y] / (1 + (U[x, y] / Uo[x, y]) ^M[x, y]) + Lo;

Rawdata = Import[
    "\Users\Jake\Desktop\Revision Experiment\PAO1 160 mM Bicarbonate.xlsx"][[2]];

R = 10;

Q = 10;

ODdata = ToExpression[Rawdata[[2 ;; R, 2 ;; Q]]];

Errdata = ToExpression[Rawdata[[(R + 2) ;; (2 * R), 2 ;; Q]]];

TobRaw = ToExpression[Rawdata[[1, 2 ;; Q]]];

BicRaw = ToExpression[Rawdata[[2 ;; R, 1]]];

TobAlone = ToExpression[Rawdata[[2, 2 ;; Q]]];

BicAlone = ToExpression[Rawdata[[2 ;; R, 2]]];

TobHillData = Transpose[{TobRaw, TobAlone}];

BicHillData = Transpose[{BicRaw, BicAlone}];
```

```
Z = Min[ODdata];
```

```
TobHillnlm =
 NonlinearModelFit[ToExpression[TobHillData], {HillTo[x]}, {{t50, .2}, {mt, 1}}, x]
```

FittedModel[$-\frac{61.3046\, x^{\ll 19 \gg}}{1 + 0.939894\, x^{\ll 19 \gg}}$]

```
TobHillnlm["ParameterTable"]
```

	Estimate	Standard Error	t-Statistic	P-Value
t50	1.07657	0.276654	3.89141	0.00596451
mt	0.840136	0.187067	4.4911	0.00282835

```
TobHillnlm["AdjustedRSquared"]
```

0.943624

```
ListPlot[ToExpression[TobHillData], AxesLabel → {"Tobramycin(μg/mL)", "%Change"},
 PlotRange → {{0, (Max[TobRaw])}, {-100, 0}}]
```

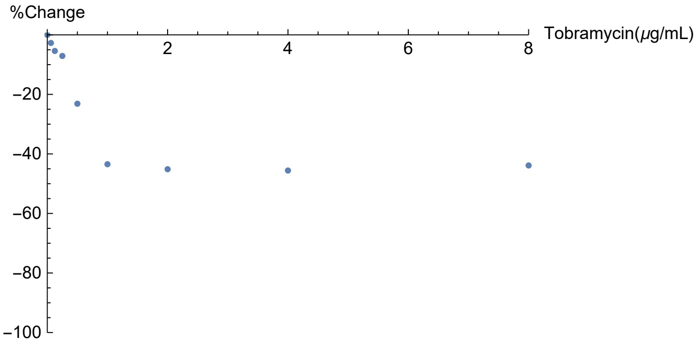


```
Show[Plot[TobHillnlm[x], {x, 0, Max[TobRaw]}, PlotRange → {0, -100}],
 ListPlot[ToExpression[TobHillData]], AxesLabel → {"Tobramycin(μg/mL)", "%Change"},
 PlotRange → {{0, (Max[TobRaw])}, {-100, 0}}]
```

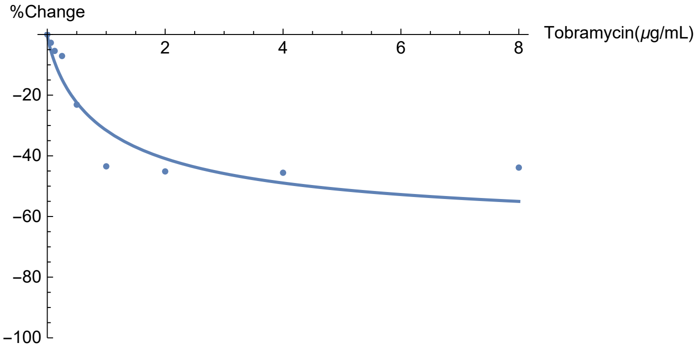


```
TobHillnlm["AdjustedRSquared"]
```

0.943624

```
TobHillnlm["ParameterTable"]
```

	Estimate	Standard Error	t-Statistic	P-Value
t50	1.07657	0.276654	3.89141	0.00596451
mt	0.840136	0.187067	4.4911	0.00282835

```
TobHillVars = TobHillnlm["BestFitParameters"]
{t50 → 1.07657, mt → 0.840136}
```

```
BicHillnlm =
 NonlinearModelFit[ToExpression[BicHillData], {HillBo[x]}, {{b50, 100}, {mb, 1}}, x]
```

FittedModel[$-\frac{0.0382949\, x^{\ll 18 \gg}}{1 + 0.000587121\, x^{\ll 18 \gg}}$]

```
ListPlot[ToExpression[BicHillData], AxesLabel → {"Bicarbonate(mM)", "%Change"},
 PlotRange → {{0, (Max[BicRaw])}, {-100, 0}}]
```

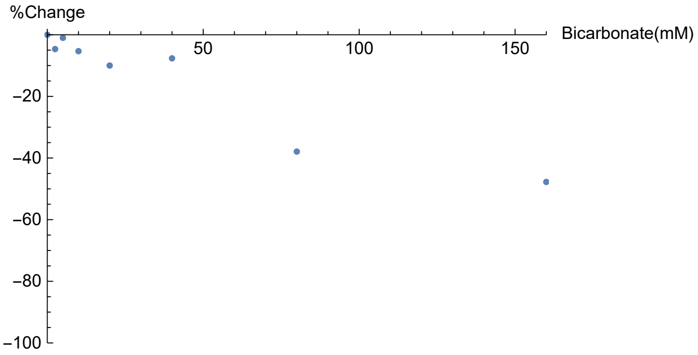


```
Show[Plot[BicHillnlm[x], {x, 0, Max[BicRaw]}, PlotRange → {0, -100}],
 ListPlot[ToExpression[BicHillData]], AxesLabel → {"Bicarbonate(mM)", "%Change"},
 PlotRange → {{0, (Max[BicRaw])}, {-100, 0}}]
```

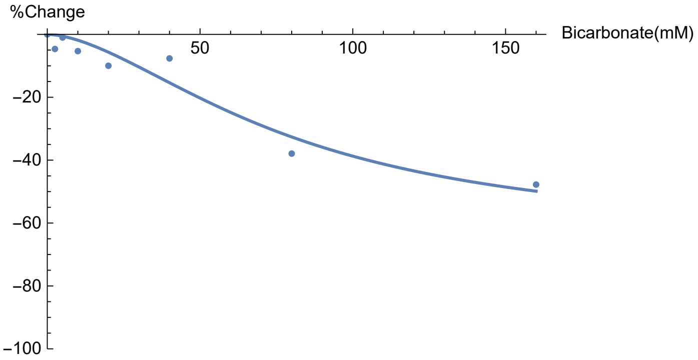


```
BicHillnlm["AdjustedRSquared"]
0.936751
```

```
BicHillnlm["ParameterTable"]
```

	Estimate	Standard Error	t-Statistic	P-Value
b50	79.9652	10.5035	7.61323	0.0001249
mb	1.69808	0.376327	4.51224	0.00275763

```
BicHillVars = BicHillnlm["BestFitParameters"]
```

{b50 → 79.9652, mb → 1.69808}

```
TobNorm = TobRaw / t50 /. TobHillVars
```

{0.0000928873, 0.0557324, 0.116109, 0.232218, 0.464437, 0.928873, 1.85775, 3.71549, 7.43099}

```
BicNorm = BicRaw / b50 /. BicHillVars
```

{1.25054×10^{-6}, 0.0156318, 0.0312636, 0.0625272, 0.125054, 0.250109, 0.500218, 1.00044, 2.00087}

```
LoeweAsum = Flatten[{TobHillVars, BicHillVars}]
```

{t50 → 1.07657, mt → 0.840136, b50 → 79.9652, mb → 1.69808}

```
LoewePars = {x50 → 1, mx → mt, y50 → 1, my → mb, Lo → 0, Lf → Z} /. LoeweAsum
```

{x50 → 1, mx → 0.840136, y50 → 1, my → 1.69808, Lo → 0, Lf → -65.225}

```
LowS = Plot3D[(L[x, y] /. LoewePars), {x, 0, (Max[TobNorm])},
  {y, 0, (Max[BicNorm])}, Mesh → Automatic, MeshFunctions → {#3 &},
  MaxRecursion → 3, ColorFunction → "BlueGreenYellow", PlotStyle → {Opacity[0.8]},
  PlotRange → {-70, 0}, AxesLabel → {"T/T50", "B/B50", "% Change"}]
```

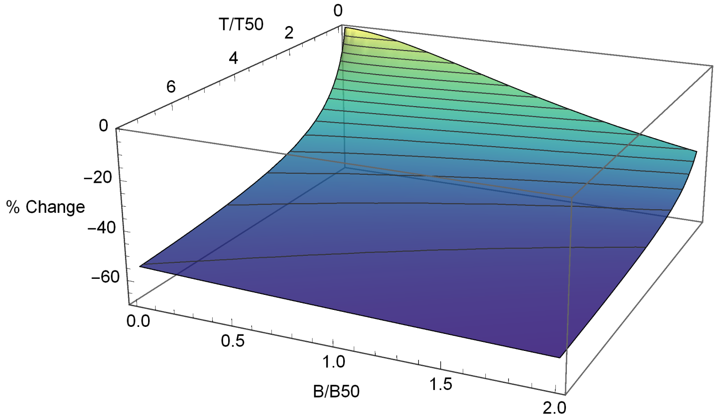


```
ODObs = Flatten[ToExpression[Table[{TobNorm[[i]], BicNorm[[j]], ODdata[[j, i]]},
    {i, 1, Length[TobNorm]}, {j, 1, Length[BicNorm]}]], 1];
```

```
ObsS =
 ListPlot3D[ODObs, PlotRange → {{0, (Max[TobNorm])}, {0, Max[BicNorm]}, {0, -70}},
  Mesh → Automatic, ColorFunction → "SandyTerrain", MeshFunctions → {#3 &},
  PlotStyle → {Opacity[0.9]}, AxesLabel → {"T/T50", "B/B50", "% Change"}]
```

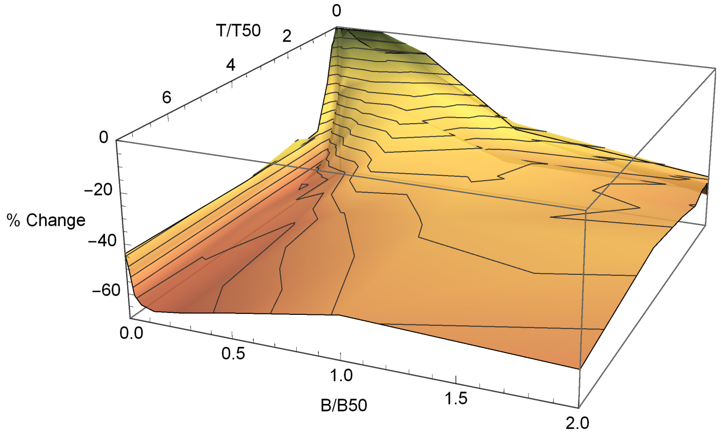


```
Show[LowS, ObsS, PlotRange → {{0, Max[TobNorm]}, {0, Max[BicNorm]}, {0, -70}},
 AxesLabel → {"T/T50", "B/B50", "% Change"}]
```

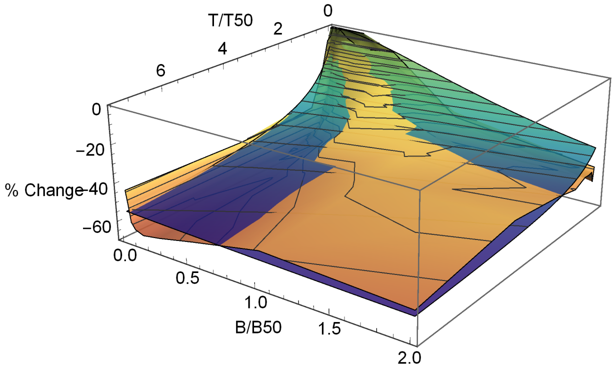


```
LPredict = Table[Table[(L[t, b] /. LoewePars), {t, ToExpression[TobNorm]}],
   {b, ToExpression[BicNorm]}];

Diff = ToExpression[ODdata] - ToExpression[LPredict];

IDataRawUp = ToExpression[
   Table[{{TobNorm[[i]], BicNorm[[j]]}, (Diff[[j, i]] + Errdata[[j, i]])},
    {i, 1, Length[TobNorm]}, {j, 1, Length[BicNorm]}]];

IDataUp = Flatten[IDataRawUp, 1];
```

```
IntpUp = Interpolation[ToExpression[IDataUp]]
```

```
InterpolatingFunction[ Domain: {{0.0000929, 7.43}, {1.25 × 10^-6, 2.}}
                       Output: scalar ]
```

```
Plot3D[IntpUp[x, y], {x, Min[TobNorm], Max[TobNorm] / 8},
 {y, Min[TobNorm], Max[BicNorm]}, PlotRange → Full, MaxRecursion → 5,
 PlotStyle → {Opacity[0.8]}, ColorFunction → "DeepSeaColors",
 AxesLabel → {"T/T50", "B/B50", "% Difference"}]
```

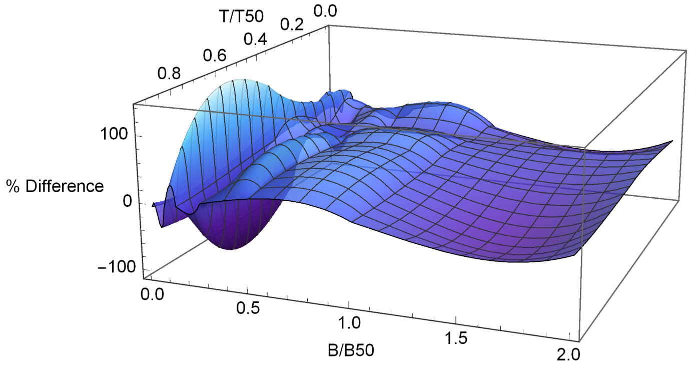


```
VUp = NIntegrate[IntpUp[x, y],
  {x, Min[TobNorm], Max[TobNorm]}, {y, Min[BicNorm], Max[BicNorm]}]
```

```
73.7082
```

```
A = ((Min[TobNorm] - Max[TobNorm]) (Min[BicNorm] - Max[BicNorm]))
```

```
14.8683
```

```
KUp = VUp / A
```

```
4.95742
```

```
IDataRawDwn = ToExpression[
   Table[{{TobNorm[[i]], BicNorm[[j]]}, (Diff[[j, i]] - Errdata[[j, i]])},
    {i, 1, Length[TobNorm]}, {j, 1, Length[BicNorm]}]];
```

```
IDataDwn = Flatten[IDataRawDwn, 1];
```

```
IntpDwn = Interpolation[ToExpression[IDataDwn]]
```

```
InterpolatingFunction[ Domain: {{0.0000929, 7.43}, {1.25 × 10^-6, 2.}}
                       Output: scalar ]
```

In[145]:=
```
Plot3D[IntpDwn[x, y], {x, Min[TobNorm], Max[TobNorm]},
 {y, Min[BicNorm], Max[BicNorm]}, PlotRange → Full, MaxRecursion → 6,
 PlotStyle → {Opacity[0.7]}, ColorFunction → "DeepSeaColors",
 AxesLabel → {"T/T50", "B/B50", "% Difference"}]
```

Out[145]=

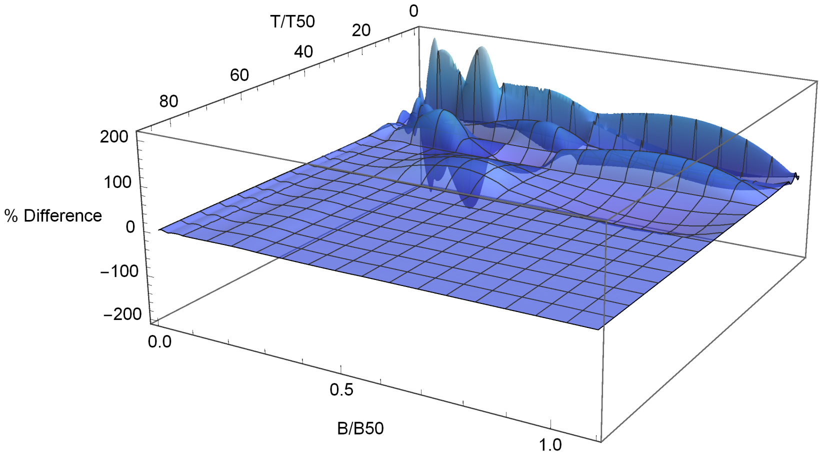


```
VDwn = NIntegrate[IntpDwn[x, y],
  {x, Min[TobNorm], Max[TobNorm]}, {y, Min[BicNorm], Max[BicNorm]}]
```

-84.2007

```
A = ((Min[TobNorm] - Max[TobNorm]) (Min[BicNorm] - Max[BicNorm]))
```

14.8683

```
KDwn = VDwn / A
```

-5.66312

```
K = (KDwn + KUp) / 2
```

-0.352848

```
SD = Abs[KUp - K]
```

5.31027
