# Supplementary material for: Tobramycin and bicarbonate synergise to kill planktonic Pseudomonas aeruginosa, but antagonise to promote biofilm survival
Source: NPJ Biofilms Microbiomes. 2016 May 25;2:16006–. doi: 10.1038/npjbiofilms.2016.6 (PMC5515257; doi:10.1038/npjbiofilms.2016.6)
Supplement: Supplementary Information [file npjbiofilms20166-s2.doc]

**TABLE S1** Summary of the efficacy of the Tobramycin-Bicarbonate combination against planktonic cells of *P. aeruginosa.* ARM = antibiotic-resistant mutant.

| **Strain** | **MIC**  **Tobramycin**  **(μg/mL)** | **MIC**  **Bicarbonate**  **(mM)** | **ΣFIC index**  **(average)** | **FIC index**  **(range)** | **Effect** |
| --- | --- | --- | --- | --- | --- |
| PAO1 | 2 | 160 | 0.37 | 0.25 – 0.75 | Synergistic/Additive |
| PA14 | 2 | 160 | 0.37 | 0.25 – 0.75 | Synergistic/Additive |
| ARM #1 | 8 | 160 | 0.49 | 0.27 - 1 | Synergistic/Additive |
| ARM #2 | 8 | 160 | 0.35 | 0.15 – 0.63 | Synergistic/Additive |
| ARM #3 | 8 | 160 | 0.45 | 0.28 – 0.75 | Synergistic/Additive |
| ARM #4 | 8 | 160 | 0.45 | 0.28 – 0.75 | Synergistic/Additive |
| 3639M | 1 | 160 | 0.48 | 0.28 – 0.75 | Synergistic/Additive |
| 4278M | 1 | 160 | 0.48 | 0.28 – 0.75 | Synergistic/Additive |
| 5623M | 8 | 80 | 0.52 | 0.31 – 0.75 | Synergistic/Additive |
| 5914M | 8 | 80 | 0.28 | 0.18 – 0.5 | Synergistic/Additive |
| 0476M | 1 | 80 | 0.48 | 0.31 – 0.63 | Synergistic/Additive |
| 4220M | 2 | 160 | 0.49 | 0.28 – 0.75 | Synergistic/Additive |
| 2159M | 2 | 160 | 0.47 | 0.28 – 0.63 | Synergistic/Additive |
| 5913C | 8 | 80 | 0.67 | 0.3 – 1 | Synergistic/Additive |
| 3470C | 1 | 160 | 0.45 | 0.28 – 0.63 | Synergistic/Additive |
| 1913C | 2 | 160 | 0.57 | 0.53 - 0.63 | Additive |
| 3488D | 4 | 80 | 0.51 | 0.31 – 0.75 | Synergistic/Additive |
| 4219D | 2 | 160 | 0.21 | 0.04 - 0.53 | Synergistic/Additive |

**TABLE S2** Corresponding pH values for different concentrations of bicarbonate in Luria-Bertani (LB) medium. SEM = standard error of the mean. LB = Luria-Bertani

| Concentration of Bicarbonate (mM) | Corresponding pH values (± SEM) measured in LB medium |
| --- | --- |
| 640 | 8.32 ± 0.02 |
| 320 | 8.14 ± 0.01 |
| 160 | 8.06 ± 0.02 |
| 80 | 7.97 ± 0.03 |
| 40 | 7.69 ± 0.01 |
| 20 | 7.51 ± 0.01 |
| 10 | 7.35 ± 0.02 |
| 5 | 7.12 ± 0.07 |
| 2.5 | 7.03 ± 0.03 |
| 1.25 | 6.97 ± 0.03 |
| 0.625 | 6.94 ± 0.03 |
| 0 | 6.9 ± 0.08 |

**TABLE S3** Efficacy of the Tobramycin-Bicarbonate combination against biofilm cells of *P. aeruginosa*.

| **Strains** | ***MBIC**  **Tobramycin**  **(μg/mL)** | **^#^MBIC**  **Bicarbonate**  **(mM)** | **ΣFIC index**  **(average)** | **ΣFIC index**  **(range)** | **Effect** |
| --- | --- | --- | --- | --- | --- |
| PAO1 | 2 | 320 | 0.65 | 0.56 – 0.75 | Additive |
| 3470C | 2 | 320 | 1.2 | 1 – 1.5 | Additive/Antagonistic |
| 4219D | 2 | 320 | 1.1 | 1 – 1.5 | Additive/Antagonistic |

* For strains PAO1 and 3470, a maximum of 80-fold reduction, and for strain 4219D, a maximum of 55-fold reduction was achievable at this concentration.

# For strains PAO1 and 3470, a maximum of 80-fold reduction, and for strain 4219D, a maximum of 60-fold reduction was achievable at this concentration.
