# Supplementary material for: Tobramycin and bicarbonate synergise to kill planktonic Pseudomonas aeruginosa, but antagonise to promote biofilm survival
Source: NPJ Biofilms Microbiomes. 2016 May 25;2:16006–. doi: 10.1038/npjbiofilms.2016.6 (PMC5515257; doi:10.1038/npjbiofilms.2016.6)
Supplement: Supplementary Figures [file npjbiofilms20166-s3.doc]

**Supplemental Material**

**Figure Legends**

**Supplementary Figures**

**FIG S1** Representative data showing the (A) percent change values and (B) ΣFIC values for the combination of tobramycin and bicarbonate for antibiotic-resistant strain #1. Different concentrations of tobramycin and bicarbonate result in 64 test combinations for each strain. MIC values for each agent were also tested alone (not shown here). In this dataset, MIC values for tobramycin and bicarbonate alone against antibiotic-resistant strain #1 are highlighted in blue. The growth-no growth interface (red boxes) was plotted along lowest combinations producing a 90% growth reduction (A). Below and to the left of this interface, test combination wells showed growth (green boxes). Corresponding ΣFIC values along the growth-no growth interface (B) were calculated using the formula


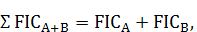
where
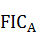
 =
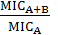
and
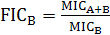
, where A and B are the test agents. The ΣFIC index was interpreted as follows: ΣFIC ≤ 0.5 indicates a synergistic interaction; ≥ 0.5 and < 1 indicates an additive effect; and > 1 indicates antagonism.

**FIG S2** Representative graphs showing synergistic (green), additive (orange), and antagonistic (purple) regimes based on (A) concentrations of tobramycin and bicarbonate and (B) ΣFIC values. The ΣFIC index was interpreted as follows: ΣFIC ≤ 0.5 indicates a synergistic interaction; ≥ 0.5 and < 1 indicates an additive effect; and > 1 indicates antagonism.

**FIG S3** Screen for biofilm formation and metabolic activity of biofilm cells for different *P. aeruginosa* strains. (A) Crystal violet assay for biofilm formation by staining biofilm mass (live cells, dead, cells, and extracellular matrix) and (B) XTT assay for metabolic activity in biofilms (proxy for live cells). Error bars represent SEM; N = 3.

**FIG S4** Response of planktonic bacteria to treatment by (A,C, E) increasing concentrations of tobramycin alone and (B, D, F) increasing concentrations of bicarbonate alone. The response from (A, B) the lab strain PAO1 and (C-E) clinical isolates from cystic fibrosis patients is monotonically decreasing and well-fit by the Hill function described in Materials and Methods.

**FIG S5** Response of biofilm bacteria to treatment by (A,C) increasing concentrations of tobramycin alone and (B,D) increasing concentrations of bicarbonate alone. The response from (A, B) the lab strain PAO1 and (C,D) a clinical isolate from a cystic fibrosis patient is monotonically decreasing and well-fit by the Hill function described in Materials and Methods. Data from clinical isolate strain 4219D is not included in this figure because these trends were not monotonic and were not well-fit by a Hill function.

**FIG S6** Isobologram analyses for tobramycin-bicarbonate combination against planktonic cells of *P. aeruginosa* laboratory strains PA14, and spontaneously generated antibiotic-resistant PA14 mutants #3 and 4. A strong synergistic-additive effect is observed against all three strains. Points along the isobologram represent the growth-no growth interface. The orange shaded area represents the additive region and the green shade area represents the synergistic region.

**FIG S7** Isobologram analyses for tobramycin-bicarbonate combination against planktonic cells of spontaneously generated antibiotic-resistant PA14 mutants #1 and 2. A strong synergistic-additive effect is observed against all three strains. Points along the isobologram represent the growth-no growth interface. The orange shaded area represents the additive region and the green shade area represents the synergistic region.

**FIG S8** Isobologram analyses for tobramycin-bicarbonate combination against planktonic cells of *P. aeruginosa* clinical cystic fibrosis (CF) mucoid isolates. A strong synergistic-additive effect is observed against all three strains. Points along the isobologram represent the growth-no growth interface. The orange shaded area represents the additive region and the green shade area represents the synergistic region.

**FIG S9** Isobologram analyses for tobramycin-bicarbonate combination against planktonic cells of *P. aeruginosa* clinical cystic fibrosis (CF) mucoid isolates. A strong synergistic-additive effect is observed against all three strains. Points along the isobologram represent the growth-no growth interface. The orange shaded area represents the additive region and the green shade area represents the synergistic region.

**FIG S10** Isobologram analyses for tobramycin-bicarbonate combination against planktonic cells of *P. aeruginosa* clinical cystic fibrosis (CF) classic and dwarf isolates. A strong synergistic-additive effect is observed against all three strains. Points along the isobologram represent the growth-no growth interface. The orange shaded area represents the additive region and the green shade area represents the synergistic region.

**FIG S11** Synergy between fixed combinations of bicarbonate and tobramycin in killing *P. aeruginosa* antibiotic-resistant mutant strains #2-4. Error bars represent SEM; N = 3. For this, the bicarbonate concentration associated with the lowest ΣFIC value for the select strain was plotted against varying tobramycin concentrations tested. For all four strains thus examined, addition of bicarbonate reduces the concentration of tobramycin required to inhibit planktonic *P. aeruginosa* cells even at tobramycin concentrations much lower than that needed to produce synergy.

**FIG S12** Synergy between fixed combinations of bicarbonate and tobramycin in killing *P. aeruginosa* clinical cystic fibrosis (CF) mucoid isolates. Error bars represent SEM; N = 3. For this, the bicarbonate concentration associated with the lowest ΣFIC value for the select strain was plotted against varying tobramycin concentrations tested. For all four strains thus examined, addition of bicarbonate reduces the concentration of tobramycin required to inhibit planktonic *P. aeruginosa* cells even at tobramycin concentrations much lower than that needed to produce synergy.

**FIG S13** Synergy between fixed combinations of bicarbonate and tobramycin in killing *P. aeruginosa* clinical cystic fibrosis (CF) classic and dwarf isolates. Error bars represent SEM; N = 3. For this, the bicarbonate concentration associated with the lowest ΣFIC value for the select strain was plotted against varying tobramycin concentrations tested. For all four strains thus examined, addition of bicarbonate reduces the concentration of tobramycin required to inhibit planktonic *P. aeruginosa* cells even at tobramycin concentrations much lower than that needed to produce synergy.

**FIG S14** Time-kill assays demonstrating the synergy between bicarbonate and tobramycin against *P. aeruginosa* strains PAO1, antibiotic-resistant PA14 mutant #1, and 5913C over 24 hours. Shown here are concentrations of tobramycin and bicarbonate tested alone and in combination, as well as concentration of each agent tested alone at MIC. Concentrations of tobramycin and bicarbonate tested represent the combinations that produced the lowest ΣFIC value. For strain PAO1, 0.25 μg/mL tobramycin and 5 mM bicarbonate; for antibiotic-resistant PA14 mutant #1, 1 μg/mL tobramycin and 5 mM bicarbonate; and for strain 5913C, 1 μg/mL tobramycin and 5 mM bicarbonate were tested alone and in combination. In addition, MIC tobramycin concentrations of 2 μg/mL for PAO1, 8 μg/mL for antibiotic-resistant PA14 mutant #1, and 8 μg/mL for 5913C, and MIC bicarbonate concentrations of 160 mM for PAO1, 160 mM for antibiotic-resistant PA14 mutant #1, and 80 mM for 5913C. Error bars represent SEM. N = 3.

**FIG S15** Isobologram analyses for tobramycin-bicarbonate combination against high density, stationary phase cells of *P. aeruginosa* PAO1. An additive effect is observed. MIC50 values were used for analysis. Points along the isobologram represent the growth-no growth interface. The orange shaded area represents the additive region and the green shaded area represents the synergistic region.

**FIG S16** Measurements of optical density for PAO1 introduced from stationary-phase, overnight cultures, into different tobramycin + bicarbonate combinations. These growth curves were used to measure the lag time associated with transitioning to exponential growth.

**FIG S17** Measurements of optical density for PAO1 introduced from stationary-phase, overnight cultures, into different tobramycin concentrations (with no bicarbonate present). These growth curves were used to measure the lag time associated with transitioning to exponential growth.

**FIG S18** Measurements of optical density for PAO1 introduced from stationary-phase, overnight cultures, into different bicarbonate concentrations (with no tobramycin present). These growth curves were used to measure the lag time associated with transitioning to exponential growth.

**FIG S19** Calculated response surfaces assuming Loewe additivity for tobramycin and bicarbonate treatments of (A) PAO1 planktonic bacteria and (B) PAO1 biofilms. Contour lines show increments of 10% change. Tobramycin and bicarbonate concentrations are plotted as the fraction of the concentration that produced 50% inhibition.

**FIG S20** Calculated response surfaces assuming Loewe additivity for tobramycin and bicarbonate treatments of (A) 3470C planktonic bacteria and (B) 3470C biofilms. Contour lines show increments of 10% change. Tobramycin and bicarbonate concentrations are plotted as the fraction of the concentration that produced 50% inhibition.

**FIG S17.** Calculated response surfaces for (A) 4219D planktonic bacteria and (B) 4219D biofilms. (A) For planktonic bacteria, response surfaces were calculated assuming Loewe additivity for tobramycin and bicarbonate treatments. Tobramycin and bicarbonate concentrations are plotted as the fraction of the concentration that produced 50% inhibition. (B) For biofilm bacteria, the non- monotonic response curves on the tobramycin and bicarbonate axes prevented fitting of a Hill function, so the response surface was instead estimated as a strictly-additive effect of tobramycin and bicarbonate at their respective concentrations. Contour lines show increments of 10% change.

**FIG S22.** Response-surface analyses for tobramycin-bicarbonate combination against high density, stationary-phase cells of *P. aeruginosa* PAO1. A net additive effect is observed. (A) The measured response surface is nearly flat, and very slightly concave-down. This case is intermediate between the strongly concave-up measured response for PAO1 planktonic bacteria (Fig. 5A) and the strongly concave-down measured response for PAO1 biofilm bacteria (Fig. 5B). (B) Ideally-additive surface calculated using the Hill function, as described in the Methods section. (C) The difference surface shows both positive and negative regions. These regions essentially cancel out, as the integral of the difference surface gives a value of -0.353 ± 5.310, so the net effect is nearly purely additive.
