# Supplementary figures and images for: Tobramycin and bicarbonate synergise to kill planktonic Pseudomonas aeruginosa, but antagonise to promote biofilm survival
Source: NPJ Biofilms Microbiomes. 2016 May 25;2:16006–. doi: 10.1038/npjbiofilms.2016.6 (PMC5515257; doi:10.1038/npjbiofilms.2016.6)

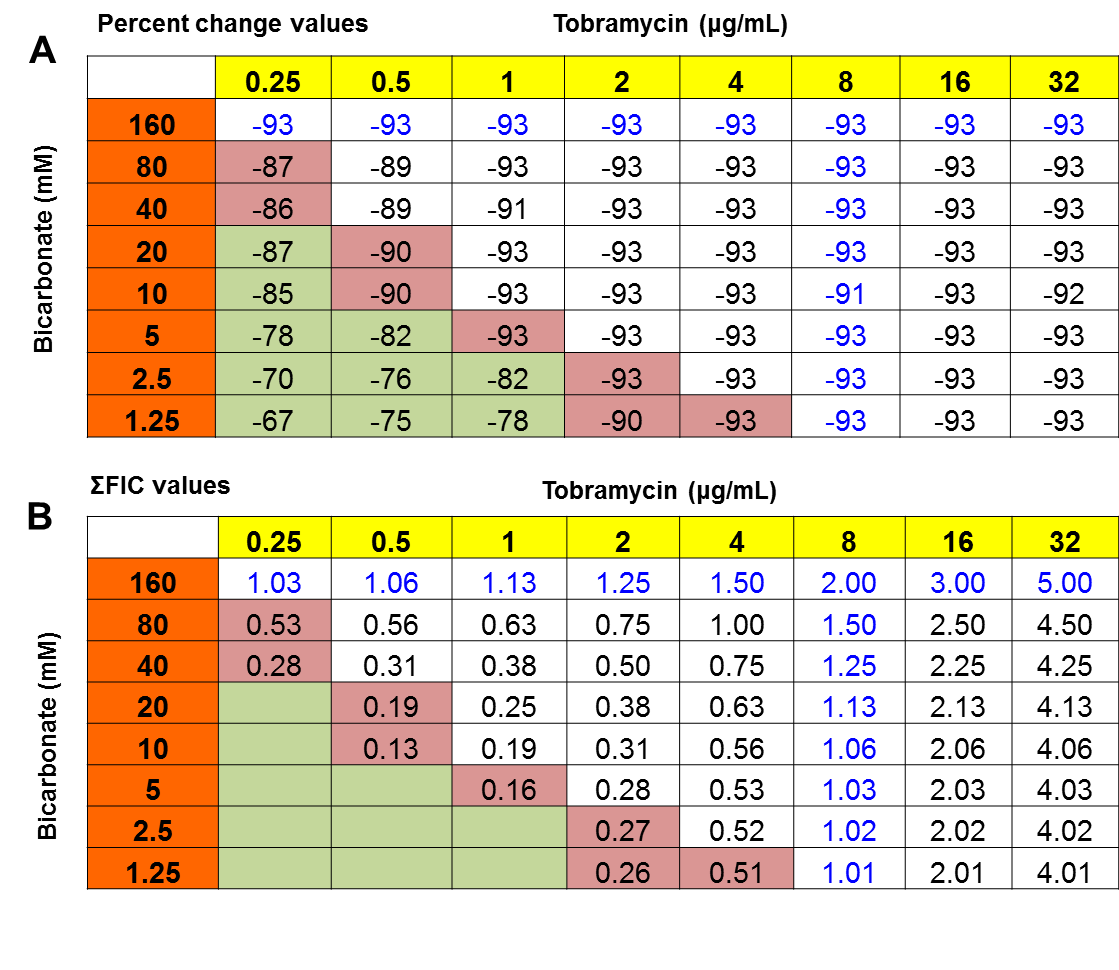

Supplement: Supplementary Figure S1 [file npjbiofilms20166-s5.tiff]

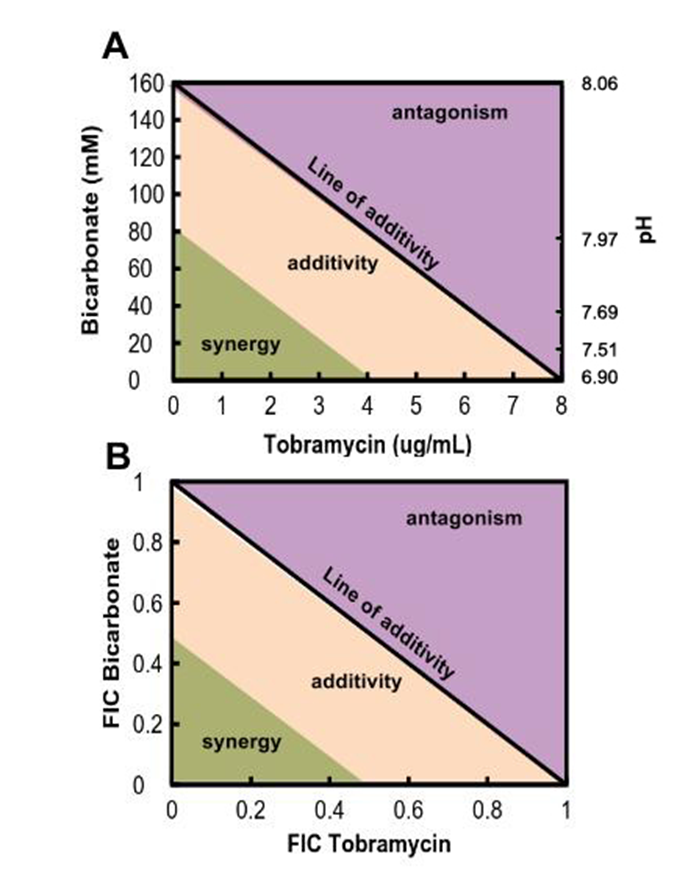

Supplement: Supplementary Figure S2 [file npjbiofilms20166-s6.tiff]

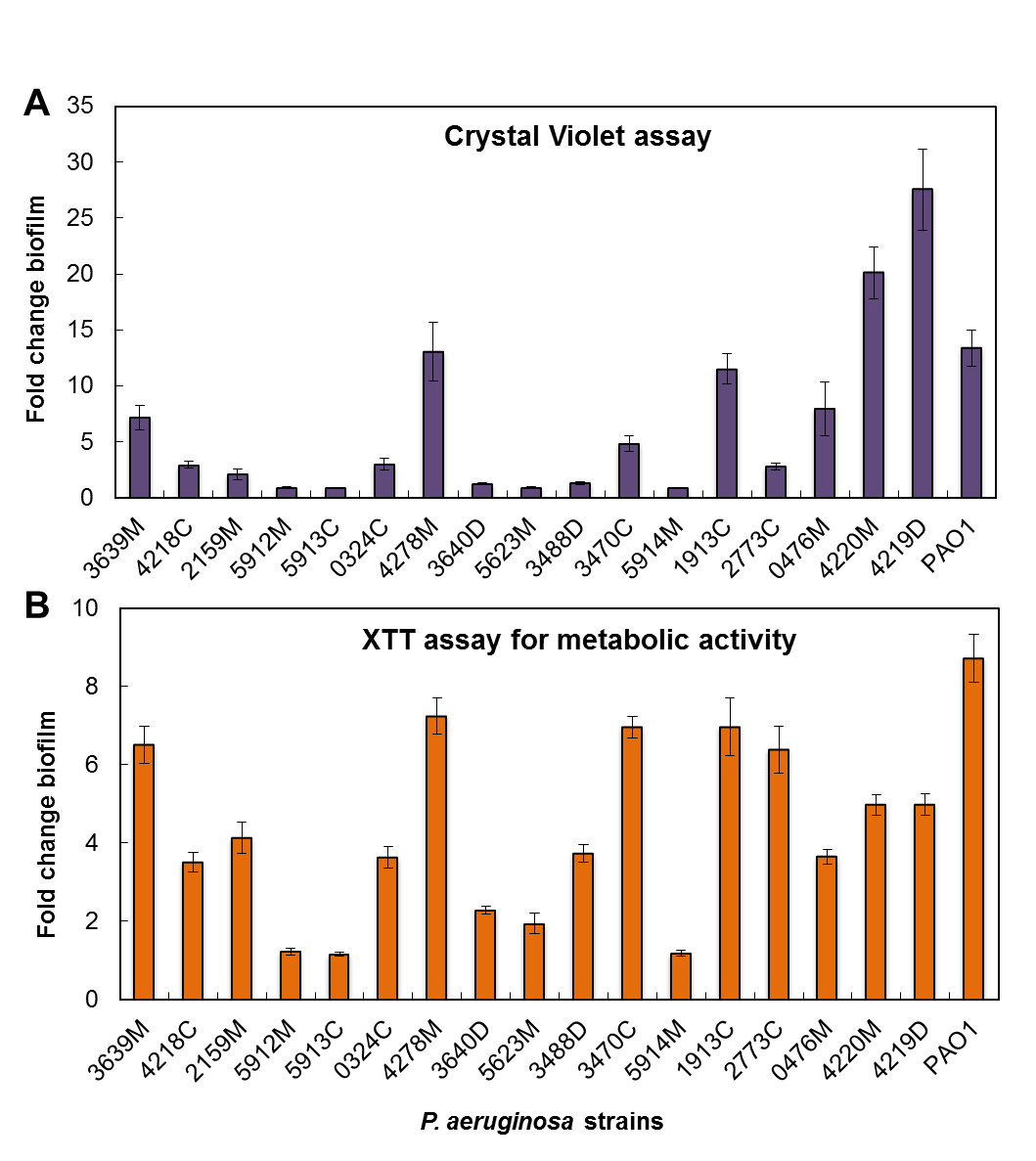

Supplement: Supplementary Figure S3 [file npjbiofilms20166-s7.tiff]

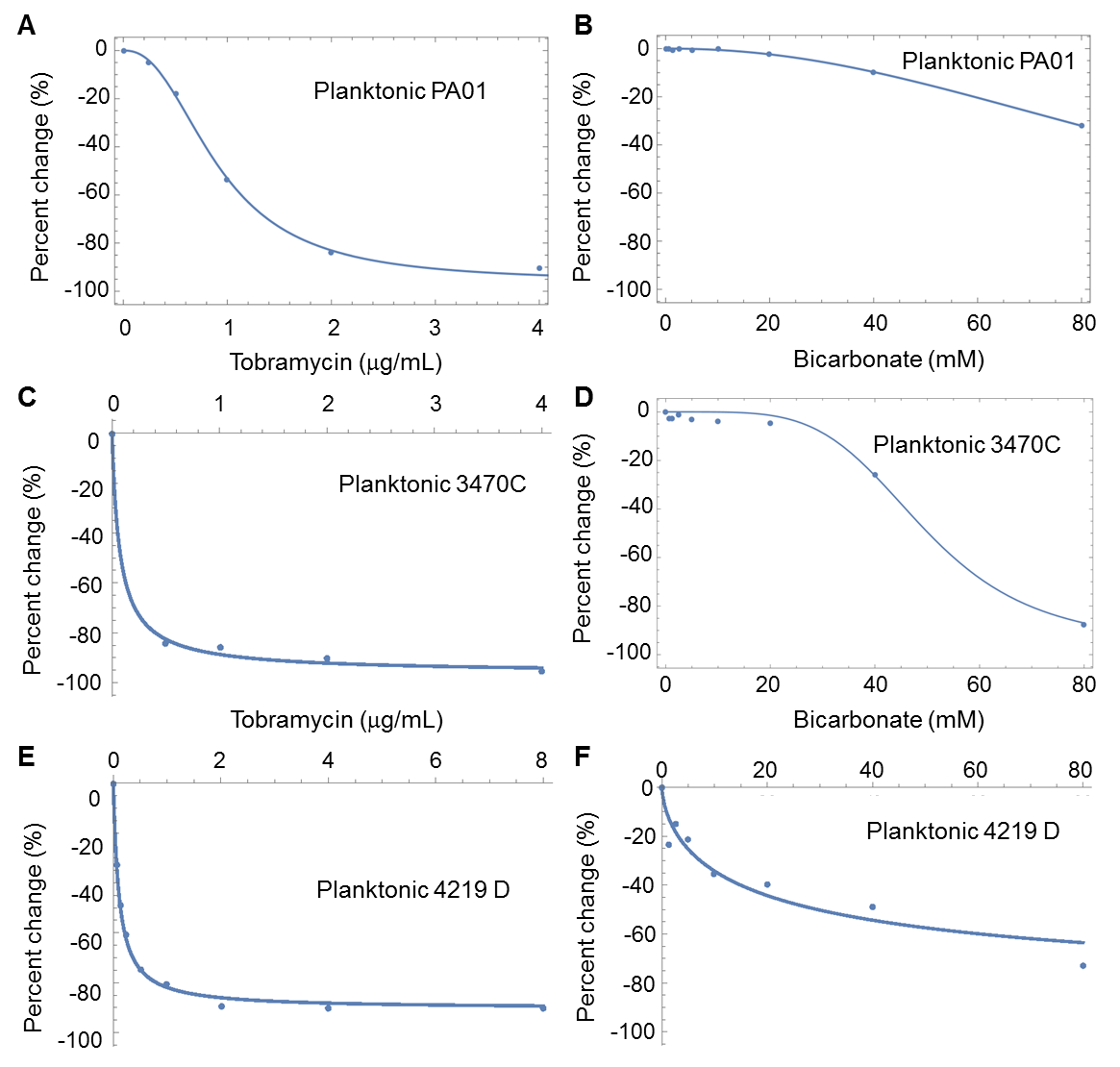

Supplement: Supplementary Figure S4 [file npjbiofilms20166-s8.tiff]

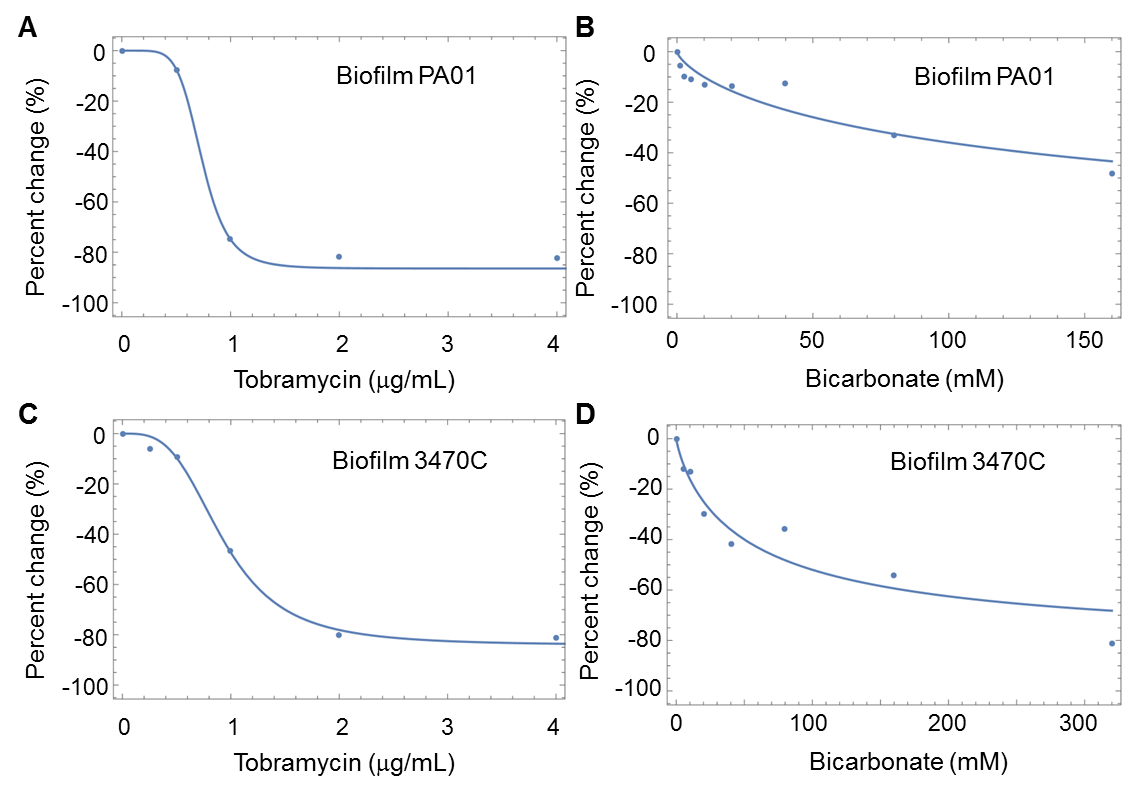

Supplement: Supplementary Figure S5 [file npjbiofilms20166-s9.tiff]

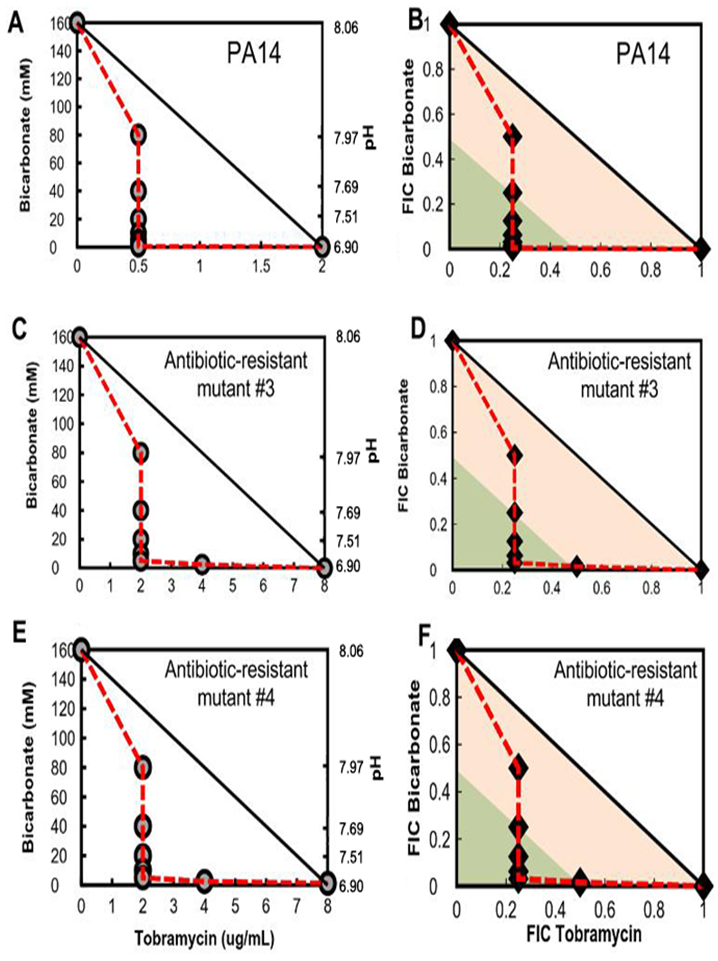

Supplement: Supplementary Figure S6 [file npjbiofilms20166-s10.tiff]

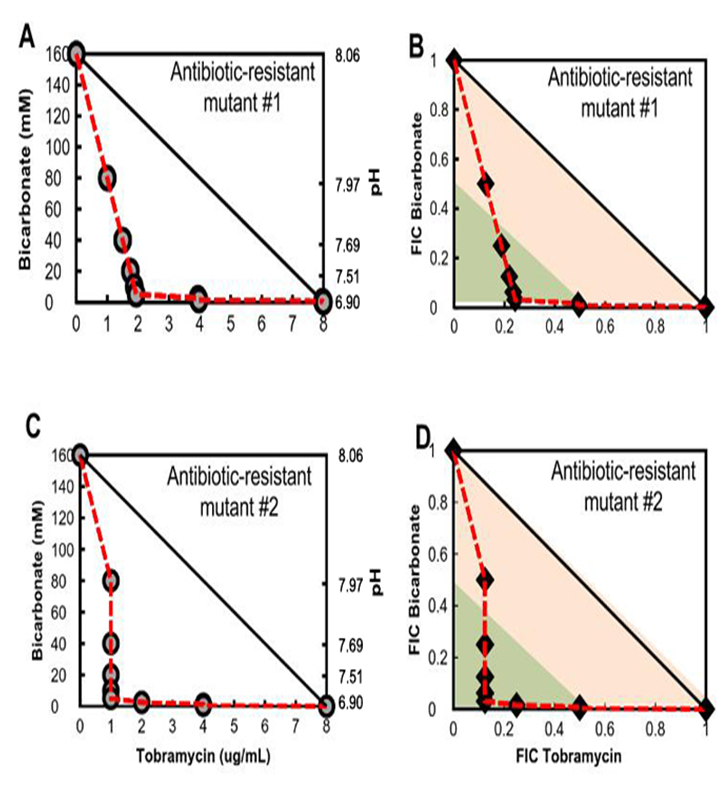

Supplement: Supplementary Figure S7 [file npjbiofilms20166-s11.tiff]

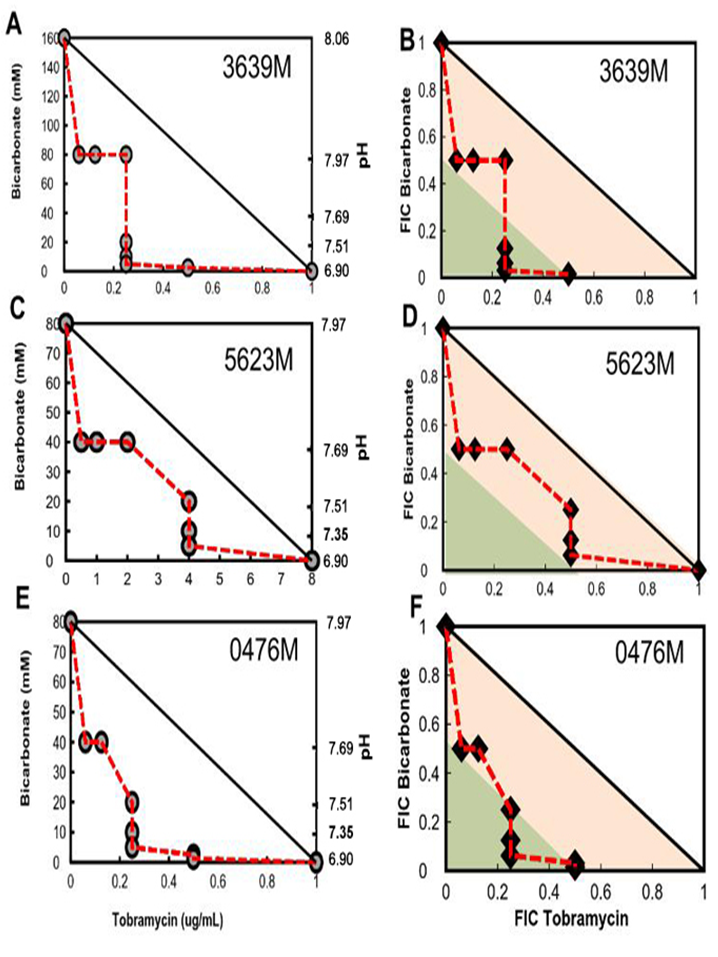

Supplement: Supplementary Figure S8 [file npjbiofilms20166-s12.tiff]

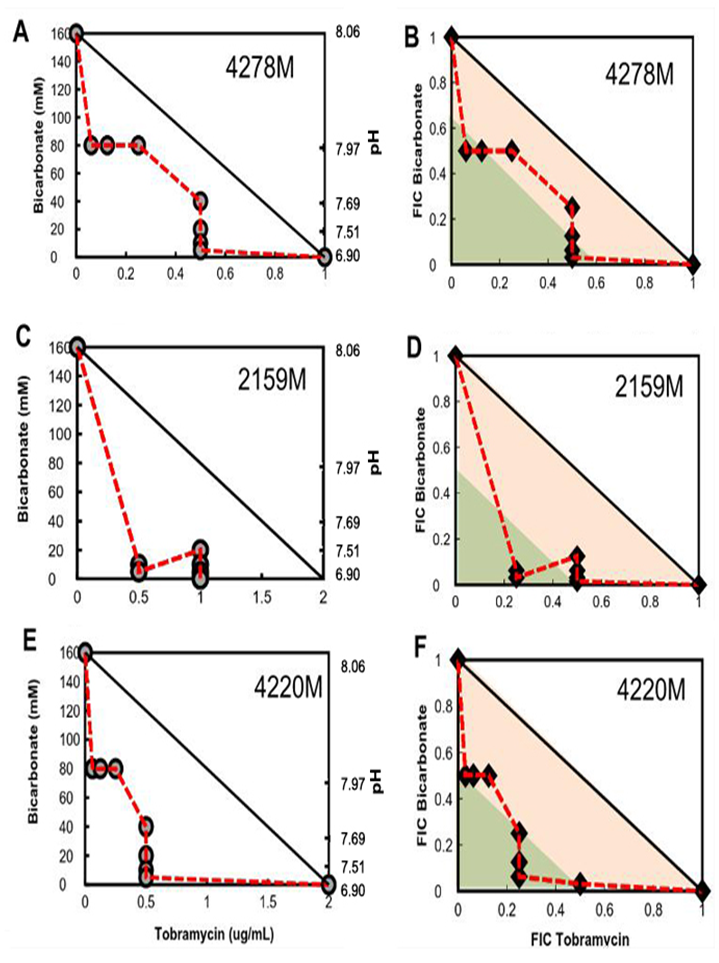

Supplement: Supplementary Figure S9 [file npjbiofilms20166-s13.tiff]

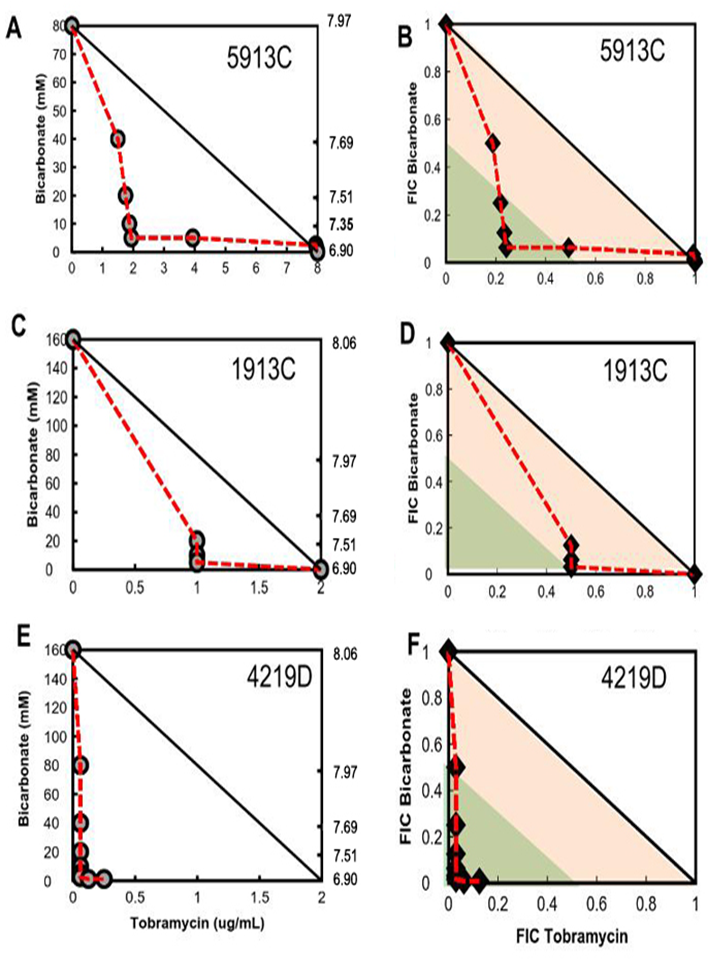

Supplement: Supplementary Figure S10 [file npjbiofilms20166-s14.tiff]

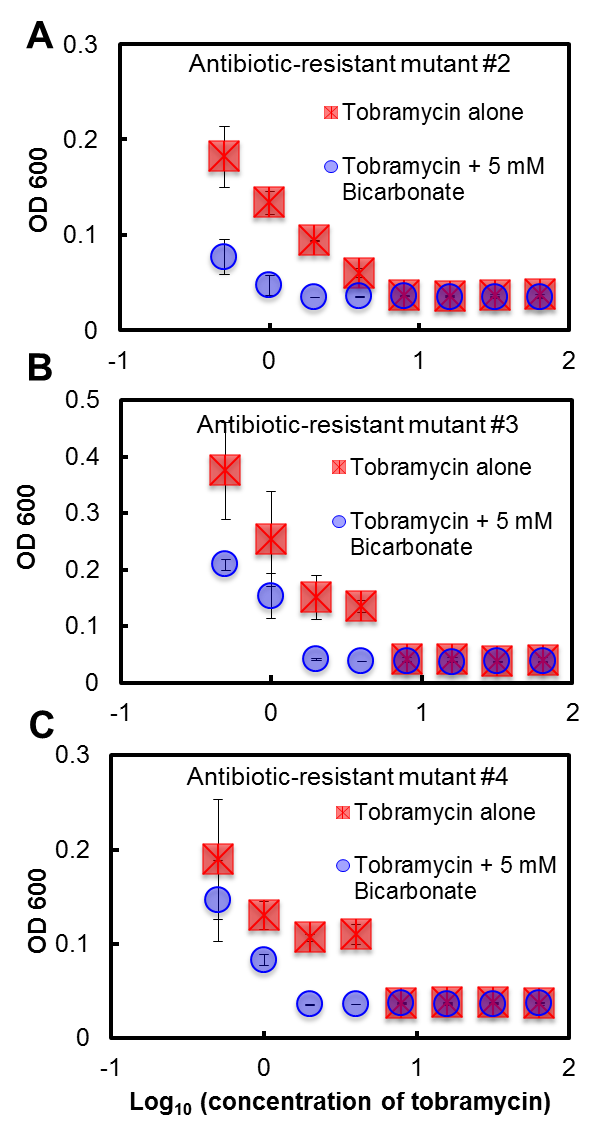

Supplement: Supplementary Figure S11 [file npjbiofilms20166-s15.tiff]

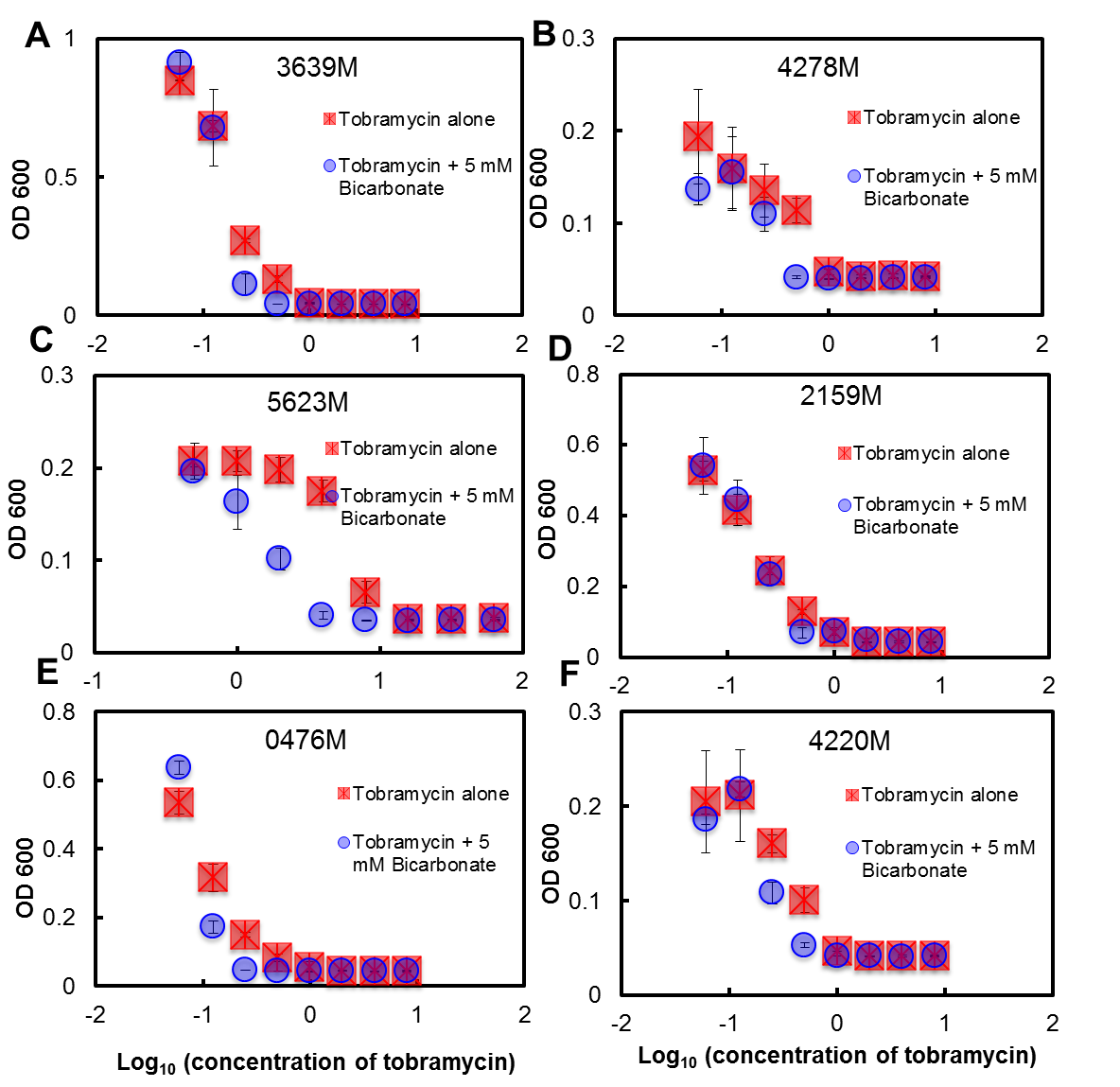

Supplement: Supplementary Figure S12 [file npjbiofilms20166-s16.tiff]

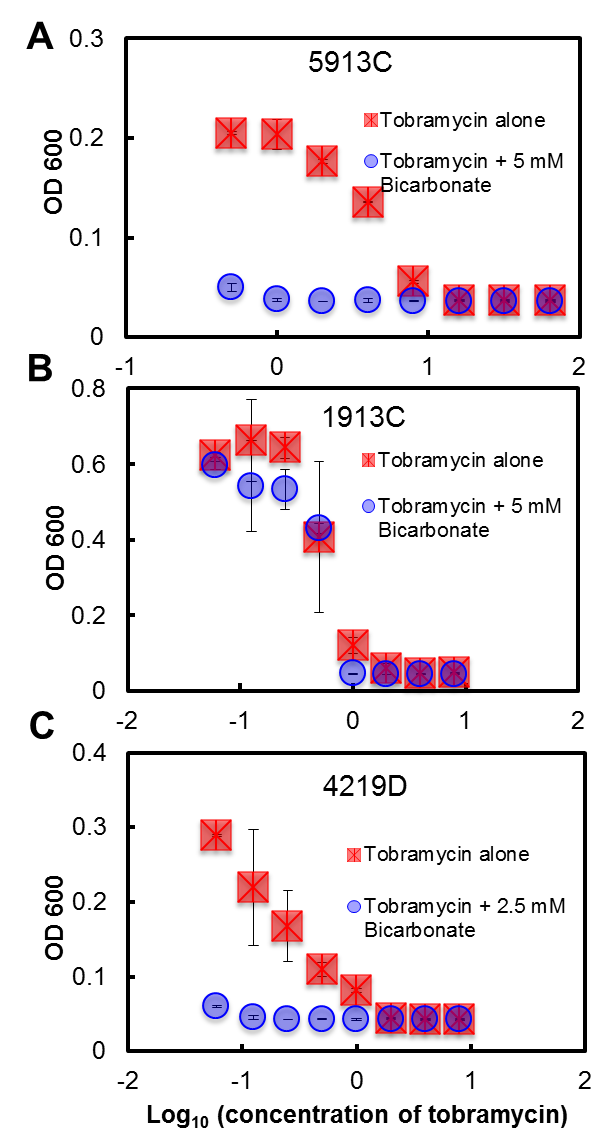

Supplement: Supplementary Figure S13 [file npjbiofilms20166-s17.tiff]

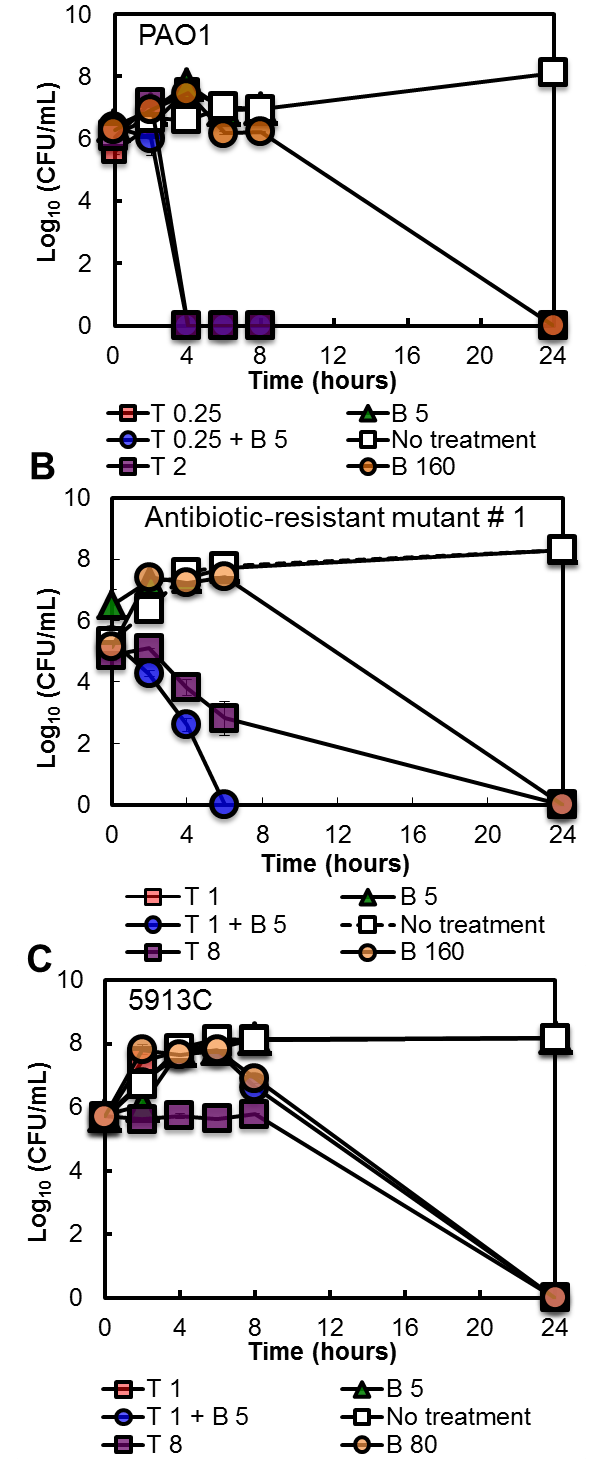

Supplement: Supplementary Figure S14 [file npjbiofilms20166-s18.tiff]

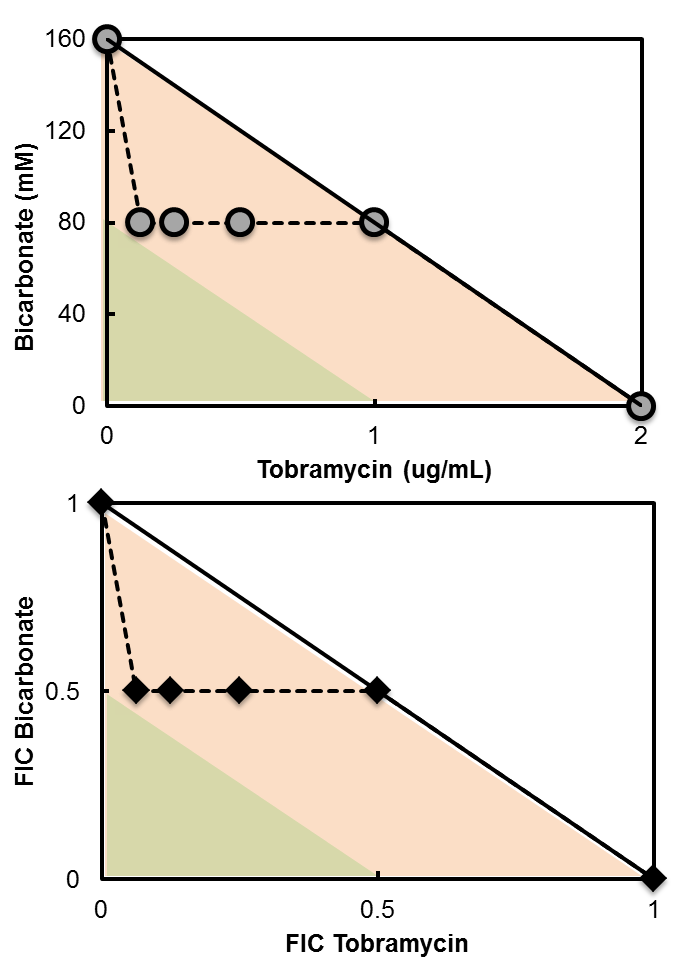

Supplement: Supplementary Figure S15 [file npjbiofilms20166-s19.tiff]

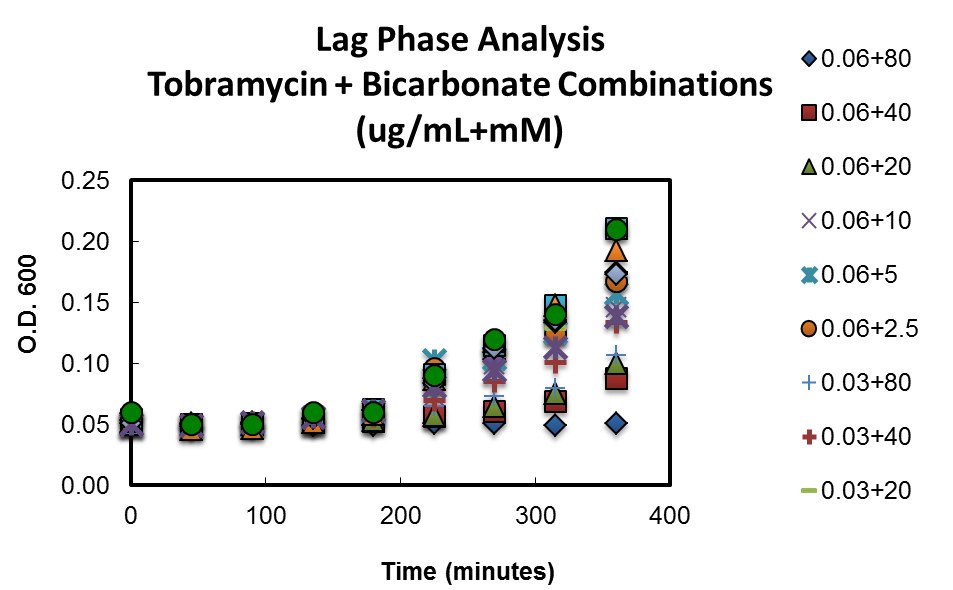

Supplement: Supplementary Figure S16 [file npjbiofilms20166-s20.tiff]

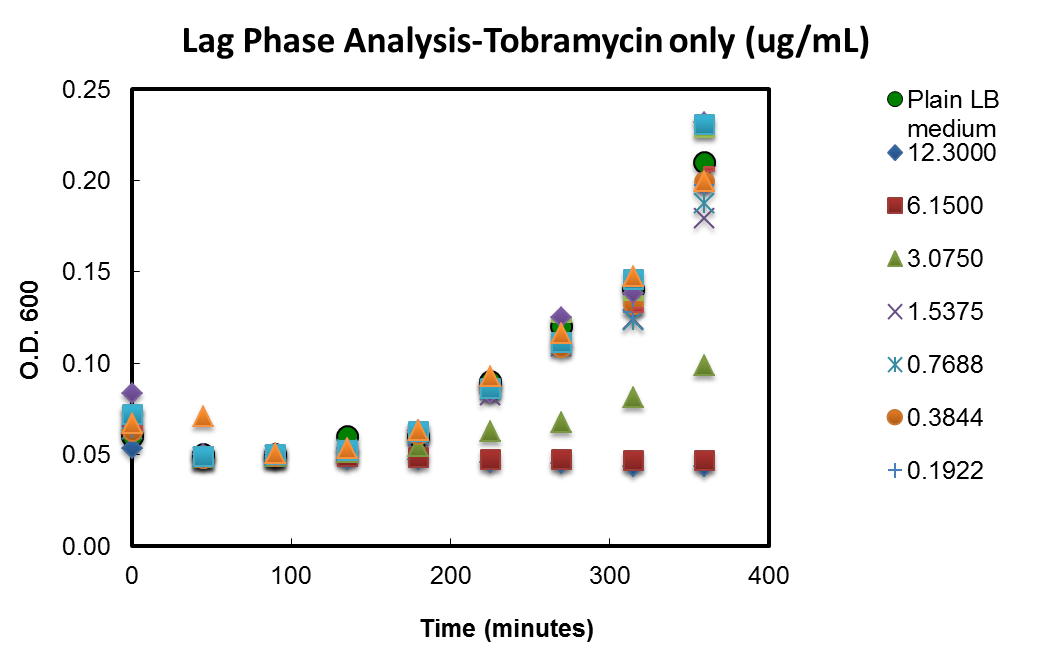

Supplement: Supplementary Figure S17 [file npjbiofilms20166-s21.tiff]

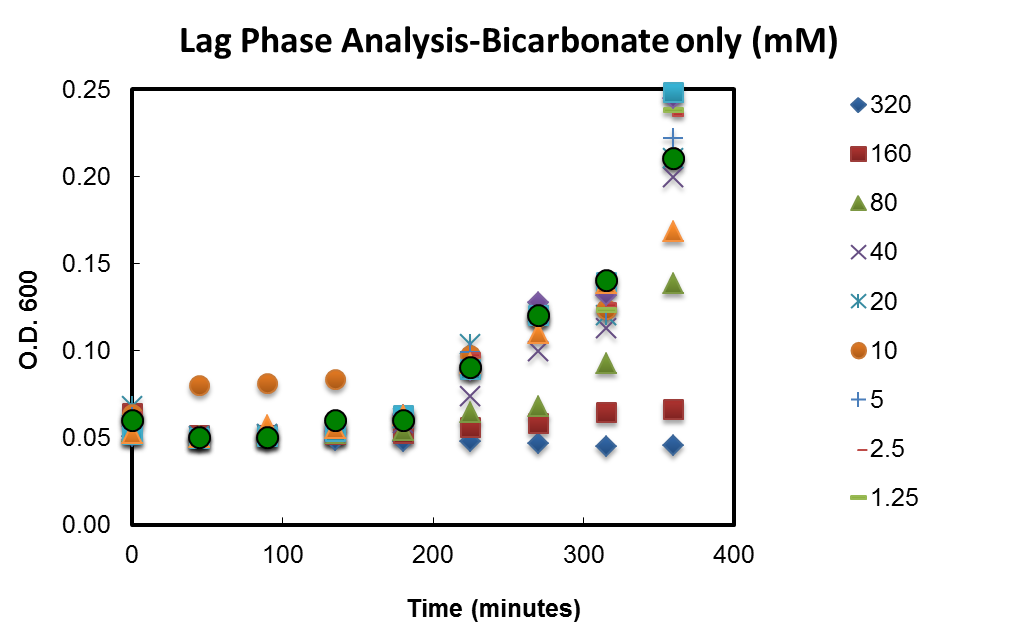

Supplement: Supplementary Figure S18 [file npjbiofilms20166-s22.tiff]

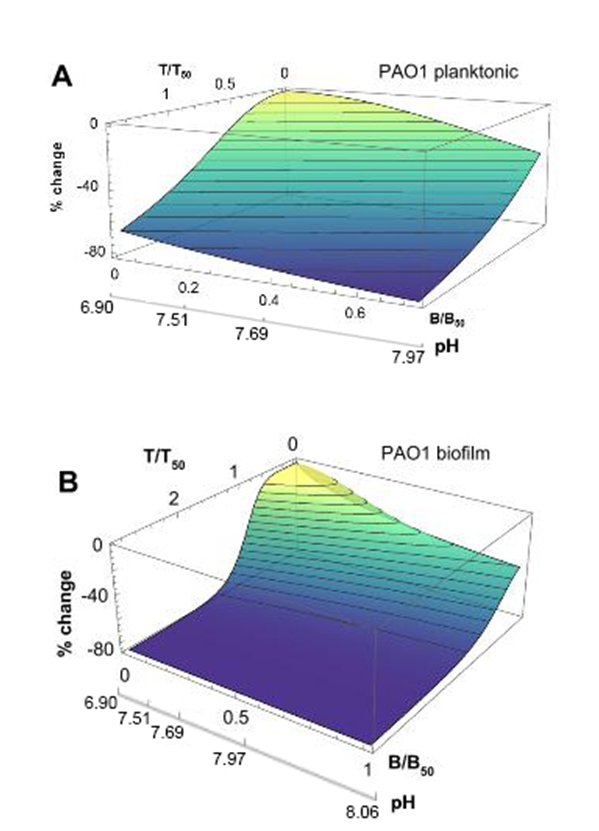

Supplement: Supplementary Figure S19 [file npjbiofilms20166-s23.tiff]

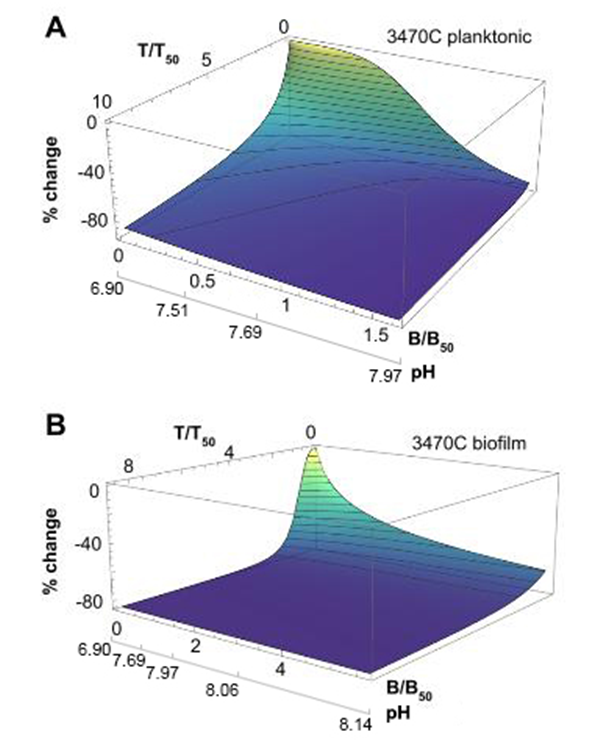

Supplement: Supplementary Figure S20 [file npjbiofilms20166-s24.tiff]

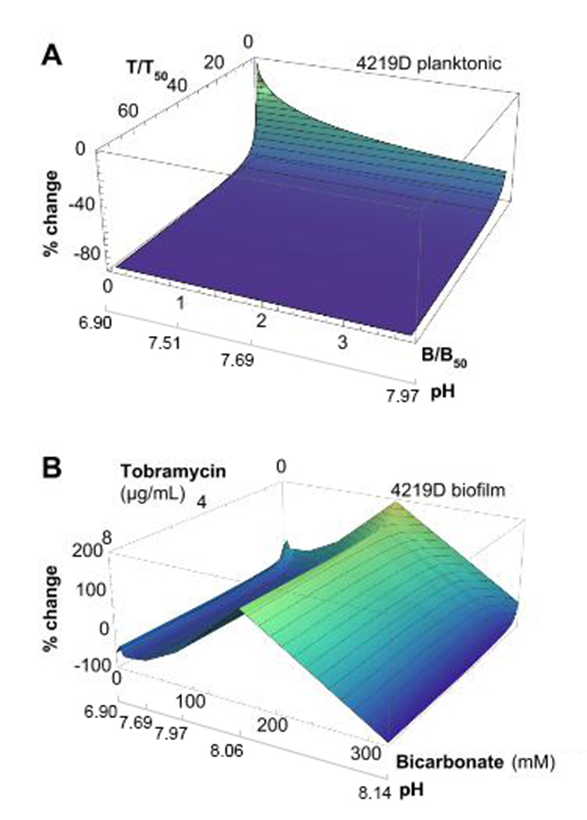

Supplement: Supplementary Figure S21 [file npjbiofilms20166-s25.tiff]

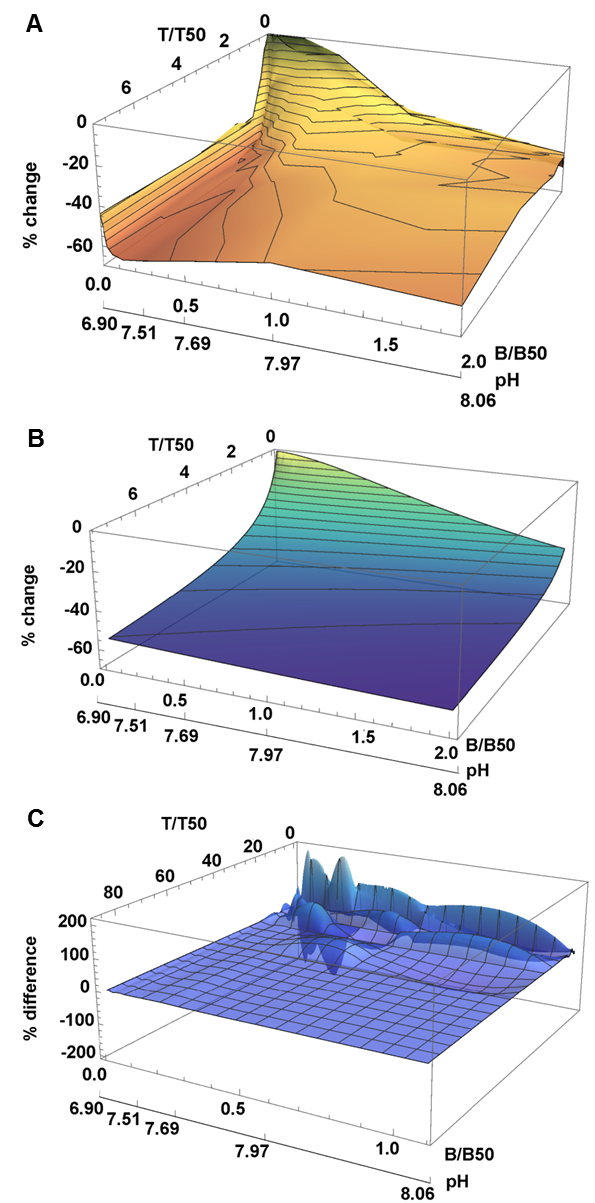

Supplement: Supplementary Figure S22 [file npjbiofilms20166-s26.tiff]
